# Analysis of the *Rickettsia africae *genome reveals that virulence acquisition in *Rickettsia *species may be explained by genome reduction

**DOI:** 10.1186/1471-2164-10-166

**Published:** 2009-04-20

**Authors:** Pierre-Edouard Fournier, Khalid El Karkouri, Quentin Leroy, Catherine Robert, Bernadette Giumelli, Patricia Renesto, Cristina Socolovschi, Philippe Parola, Stéphane Audic, Didier Raoult

**Affiliations:** 1 Unité des rickettsies, IFR 48 CNRS UMR 6020, Faculté de médecine, Université de la Méditerranée, 27 Boulevard Jean Moulin, 13385 Marseille cedex 05, France; 2 Information Génomique et Structurale, CNRS UPR2589, Institut de Biologie structurale et Microbiologie, Marseille, France

## Abstract

**Background:**

The *Rickettsia *genus includes 25 validated species, 17 of which are proven human pathogens. Among these, the pathogenicity varies greatly, from the highly virulent *R. prowazekii*, which causes epidemic typhus and kills its arthropod host, to the mild pathogen *R. africae*, the agent of African tick-bite fever, which does not affect the fitness of its tick vector.

**Results:**

We evaluated the clonality of *R. africae *in 70 patients and 155 ticks, and determined its genome sequence, which comprises a circular chromosome of 1,278,540 bp including a *tra *operon and an unstable 12,377-bp plasmid. To study the genetic characteristics associated with virulence, we compared this species to *R. prowazekii*, *R. rickettsii *and *R. conorii*. *R. africae *and *R. prowazekii *have, respectively, the less and most decayed genomes. Eighteen genes are present only in *R. africae *including one with a putative protease domain upregulated at 37°C.

**Conclusion:**

Based on these data, we speculate that a loss of regulatory genes causes an increase of virulence of rickettsial species in ticks and mammals. We also speculate that in *Rickettsia *species virulence is mostly associated with gene loss.

The genome sequence was deposited in GenBank under accession number [GenBank: NZ_AAUY01000001].

## Background

Rickettsiae are obligate intracellular Gram-negative bacteria mostly associated to arthropods, some of which causing mild to severe diseases in humans. Pathogenic species are classified into two groups based on phylogenetic analyses [1]. The typhus group (TG) includes two *Rickettsia prowazekii *(*R. prowazekii*) and *R. typhi*, and the spotted fever group (SFG) includes 15 pathogenic species and numerous species of unknown pathogenicity [2,3]. Two additional validated species, *R. bellii *and *R. canadensis*, and a variety of unvalidated species from insects or leeches are organized into the most outer outgroups of the genus *Rickettsia *[3-5]. The relatively low rate of lateral gene transfer, the continuous gene loss and the colinearity of most of their genomes make *Rickettsia *species an outstanding model for comparative genomics [4,6,7]. Indeed, genome reduction [8] paradoxically results in higher virulence in *R. prowazekii*.

The pathogenic mechanisms of rickettsiae are unclear. Within ticks, rickettsiae remain quiescent during the starvation of their vector but undergo a reversion to the virulent state, termed reactivation, following incubation at 37°C or blood meal [9]. This phenomenon is marked in *R. rickettsii *by morphological changes in the microcapsular and slime layers [9]. The precise molecular mechanisms of this change, however, are only poorly understood. During human infection, attachment to and invasion of host cells were suggested to involve the outer membrane proteins rOmpA and rOmpB and the adhesins Adr1 and Adr2 [10,11]. A phospholipase D activity was proposed to play a role in escape from phagosomes [8,12], and intracellular motility was demonstrated to rely on actin polymerization [13,14]. None of these factors nor the presence of a type IV secretion system [15], however, explain the virulence differences observed among *Rickettsia *species [6].

Over the last ten years, *R. africae *has emerged as the causative agent of African tick-bite fever [2], the most common SFG rickettsiosis both in terms of seroprevalence [16] and incidence [17-20]. Such an epidemiologic success is due to various factors, including the increase of tourism to wildlife parks in sub-Saharan Africa, the attack host-seeking behavior of its vector ticks,*Amblyomma *sp., and the elevated prevalence of *R. africae *in these ticks, with infection rates of up to 100% [21]. In addition, the bacterium has been identified in other areas with warm climates, such as the West Indies, where it was found in Guadeloupe, Martinique, St Kitts and Nevis, and Antigua islands [2]. Such a distribution, as well as the presence of *R. africae *in Reunion island, is likely to result from the transfer from Africa of cattle bearing infected ticks [2]. Tick-associated rickettsiae may infect ticks feeding on infected hosts or may be passed from one generation to the next transovarially. *R. africae *is transmitted transovarially and appears to be the most successful rickettsia in its adaptation to its vector tick, as the prevalence of tick infection is higher than that of any other rickettsia [22]. In addition, infection does not appear to alter tick fitness (P. Parola, unpublished data). These data highlight the fact that *R. africae *is an extremely successful and fit bacterium.

By comparison with *R. conorii*, the second most prevalent SFG rickettsia in Africa, whose genome has previously been sequenced [23], *R. africae *exhibits a higher prevalence in ticks [2], a lower virulence in humans [17], and a greater genetic homogeneity [24]. The genetic factors underlying these characteristics are, however, unknown. We assumed that the *R. africae *genome sequence might help understand the characteristics of this species and the genetic mechanisms associated with the difference in virulence. Here, we present the sequence of the *R. africae *genome and additional data that suggest that this species has emerged recently. In support of this hypothesis, we show that *R. africae *is a clonal population. We also present data that support the assumption that rickettsial virulence increases following gene inactivation.

## Results

### General Features of the Genome

**Figure 1:**
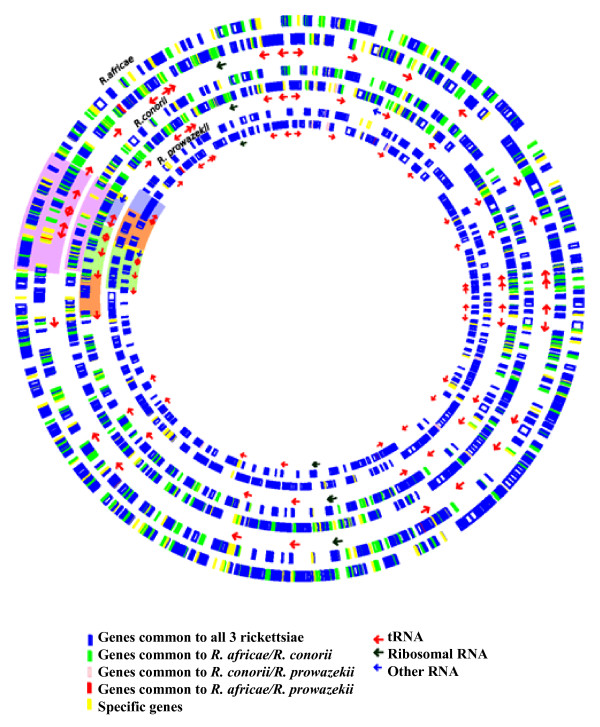
**Circular representation of the genomes of *R. africae, R. conorii*, and *R. prowazekii *based on data from GenBank entries **[GenBank: NZ_AAUY01000001], [GenBank: NC_003103] **and **[GenBank: NC_000963], **respectively**. Protein coding genes common to all species are in blue; genes common to *R. africae *and *R. conorii *are in green; genes common to *R. africae *and *R. prowazekii *are in red; genes common to *R. conorii *and *R. prowazekii *are in pink and specific genes in each genomes are in yellow. Common genes are identified using best BLAST match. The region of rearrangement of the genome between *R. africae *and *R. conorii *is colored in purple; the regions of rearrangment between *R. prowazekii *and *R. conorii *are colored in orange, light green, yellow and light blue. Also represented are transfer RNAs (red arrows), ribosomal RNAs (dark arrows) and other RNAs.

**Figure 2:**
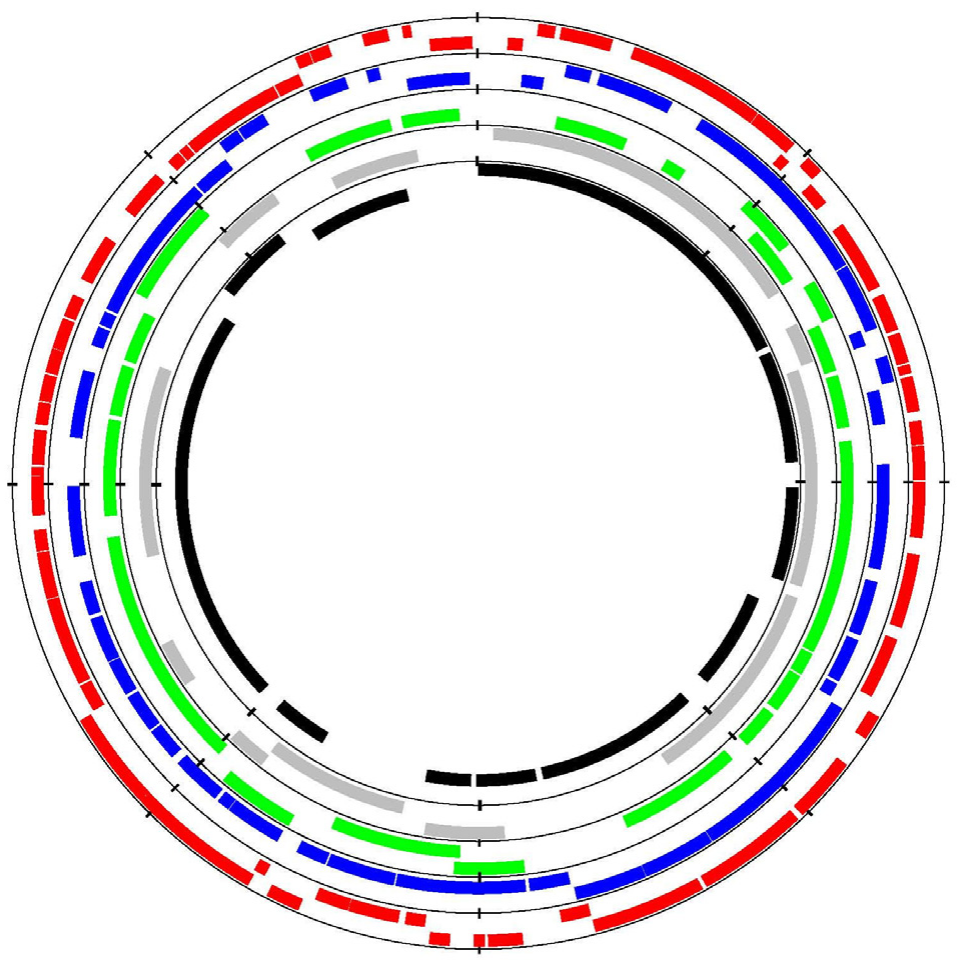
**Circular representation of *Rickettsia *plasmids**. A) The pRA plasmid: circles indicate (from the outside to the inside, on the reverse and forward strands) the GC skew, GC content, and ORFs; B) *Rickettsia *plasmids sequenced to date: circles indicate (from the outside to the inside, on the reverse and forward strands) *R. felis *pRF plasmid (red), *R. felis *pRFδ plasmid (blue), *R. monacensis *pRM plasmid (green), *R. massiliae *pRMa plasmid (grey), and *R. africae *pRA plasmid (black).

**Figure 3:**
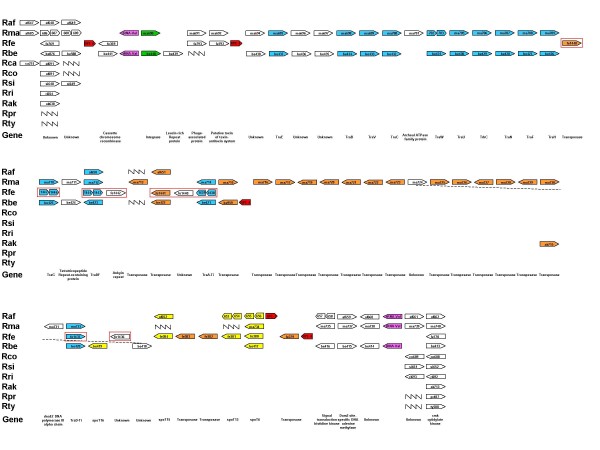
**Presence of the *tra *gene cluster in *Rickettsia *species**. Raf: *R. africae*; Rma: *R. massiliae*; Rfe: *R. felis*; Rbe: *R. bellii*; Rco: *R. conorii*; Rsi: *R. sibirica*; Rri: *R. rickettsii*; Rak: *R. akari*; Rpr: *R. prowazekii*; Rty: *R. typhi*.

The genome of *R. africae *consists of two replicons: a circular chromosome of 1,278,540 base pairs (bp) (Figure 1) and a 12,377 bp circular plasmid (Table 1, Figure 2[25,26]). We acknowledge the fact that the ESF-5 strain, first isolated in 1966 [27], may have undergone loss or rearrangement of plasmid or chromosomal genes during multiple passages in cell culture. Sequences were deposited in GenBank under accession number [GenBank: NZ_AAUY01000001]. The chromosome has a G + C content of 32.4%, in the range of other SFG rickettsial genomes (32.3 – 32.5%), whereas the plasmid has a G + C content of 33.4%, similar to those of *R. felis *(33.2 and 33.6%) [28] but higher than that of *R. massiliae *plasmids (31.4%). The predicted total complement of 1,271 open reading frames (ORFs), 1,260 chromosomal (78.26% coding sequence), and 11 plasmidic (81.3% coding sequence) ORFs [see Additional file 1], is in the range of genomes from SFG rickettsiae with the exception of *R. felis*, which exhibits a larger genome (Table 1). Of these, 1,117 (87.9%) exhibited homologs in the non-redundant database, and 1,024 (80.5%) were assigned putative functions [see Additional file 2]. Overall, the 1,260 chromosomal ORFs encoded 1,112 protein-coding genes, with 87 of these being split into 2 to 10 ORFs by the presence of one to several stop codons. By comparison with other SFG genomes, *R. africae *had fewer split genes than any other species with the exception of *R. felis *(Table 1). In addition, *R. africae *exhibited a single rRNA operon, with non-contiguous 16S and 23S rRNA genes as in other rickettsial genomes, 33 tRNAs and another three RNAs. The *R. africae *chromosome exhibited an almost perfect colinearity with the *R. conorii *genome [23], with the exception of a 88,459-bp inversion [see Additional file 3]. At both extremities of the inversion, there were repeats of the *Rickettsia *palindromic element – 6 (RPE-6) familly. In this inverted fragment, *R. africae *exhibited 20 ORFs and 10 RPEs that were absent from *R. conorii*. Among these 20 ORFs, a cluster of 11 consecutive ORFs had orthologs in the 3'-extremity of the Tra cluster previously identified in the *R. massiliae *genome [29]. These 11 ORFs included *traD*F (ORF0650), a transposase (ORF0651), *spoT*15 (ORF0652), a split *spoT*13 (ORF0653/ORF0654), a split *spoT*6 (ORF0655/ORF0656), a split signal transduction histidine kinase (ORF0657/ORF0658), *dam*2, a site-specific DNA adenine methylase (ORF0659), and ORF0660 of unknown function (Figure 3). In addition to the orthologs in *R. massiliae*, these genes had orthologs in similar clusters in *R. felis*, *R. bellii*, *R. canadensis *and *O. tsutsugamushi *but were absent from all other species. As in *R. massiliae*, *R. bellii *and *R. canadensis*, the *R. africae *cluster was bounded at its 3'-end by a tRNA-Val, but, in contrast with these three species, neither an integrase with its *attI *site nor a tRNA-Val fragment marker of integration was present at the 5' end (Figure 3). The presence of a similar gene cluster inserted at the same position in several *Rickettsia *species, with a GC content different from that of the genome (29.78% *vs *32.4%, respectively, in *R. africae*) suggests that it was acquired horizontally from a common ancestor and then transmitted vertically. In *R. africae*, an *attC *site, specific to integron-inserted gene cassettes, located at the 3'-end (coordinates: 687890–688018) of the *spoT*15 gene (ORF652), supports the role of integration in the insertion of this gene cluster. *AttC *sites were also identified in *R. massiliae *(coordinates: 743029–743145), *R. felis *(coordinates: 407889–408017), and *R. bellii *(coordinates 468143–468211). Nevertheless, the presence of transposases in all species and the fact that, in *R. felis*, nine of these genes are located in the pRF plasmid support the role of several genetic mechanisms at the origin of this cluster, possibly involving plasmids, integrons and transposons. In comparison with other species containing this gene cluster, *R. africae *had the smallest number of genes. In particular, it lacked most of the Tra cluster, with the exception of *traDF*, but retained three *spoT *genes, including two degraded to pseudogenes. In *R. bellii *and *R. massiliae*, *tra *genes were described as encoding components of a type IV secretion system (T4SS) for conjugal DNA transfer [15,29]. In terms of gene content, the *R. africae *cluster was more similar to those of *R. felis *and *R. canadensis*, with the loss of the Tra cluster, the conservation of *spoT *genes and the presence of pseudogenes, than to those of *R. massiliae *and *R. bellii*, in which the Tra cluster was intact but *spoT *genes were partially degraded. Such findings suggest that species-specific evolution of this gene cluster occurred, which likely resulted from gene excisions in *R. africae*, *R. felis *and *R. canadensis*, or gene expansion by transposase duplication in *R. massiliae*.

**Table 1:** General features of *Rickettsia *genomes.

**Species (strains)**	**Genome size (bp)**	**G+C content (%)**	**Protein-coding genes**	**RNAs**	**References**
**Spotted fever group**					
					
*R. africae *(ESF-5)	1,290,917	32.4	1,123	39	Present study
Chromosome	1,278,540	32.4	1,112	39	
Plasmid pRA	12,377	33.4	11	0	
*R. akari *(Hartford)	1,23106	32.3	1,259	35	*
*R. conorii *(Malish 7)	1,268,755	32.4	1,374	39	[23]
*R. felis *(URRWXCal2)	1,587,240	32.5	1,512	39	[28]
Chromosome	1,485,148	32.5	1,444	39	
Plasmid pRF	62,829	33.6	68	0	
Plasmid pRFδ	39,268	33.2	44	0	
*R. massiliae *(Mtu5)	1,376,184	32.5	1,192	39	[29]
Chromosome	1,360,898	32.5	1,180	39	
Plasmid pRMA	15,286	31.4	12	0	
*R. rickettsii *(Sheila Smith)	1,257,710	32.5	1,345	36	*
*R. rickettsii *(Iowa)	1,268,175	32.4	1,384	37	[25]
*R. sibirica *(246)	1,250,021	32.5	1,083	36	*
**Typhus group**					
*R. prowazekii *(Madrid E)	1,111,523	29.0	834	39	[58]
*R. typhi *(Wilmington)	1,111,496	28.9	838	39	[26]
**Third group**					
*R. bellii *(RML369-C)	1,522,076	31.7	1,429	40	[15]
*R. bellii *(OSU 85–389)	1,528,980	31.6	1,476	36	*
*R. canadensis *(McKiel)	1,159,772	31.1	1,093	36	*

* Unpublished genomes available in GenBank: *R. akari *(Hartford) [GenBank: NC_009881],*R. rickettsii *(Sheila Smith) [GenBank: NC_009882], *R. sibirica *(246) [GenBank: NZ_AABW00000000], *R. bellii *(OSU 85–389) [GenBank: NC_009883], and *R. canadensis *(McKiel) [GenBank: NC_009879].

In addition to the *traD*_*F *_gene described above, the *R. africae *chromosome retained many of the components of the type IV secretion system (T4SS) involved in both DNA transfer and effector translocation in other bacteria [30], including *virB1*, *virB2 *(ORF0232), *virB3 *(ORF0128), *virB4 *(ORF0129, ORF1109), *virB6 *(ORF0130, ORF0131, ORF0132, ORF0133, ORF0134, ORF0135), *virB8 *(ORF0359, ORF0361), *virB9 *(ORF0358, ORF0362), *virB10 *(ORF0363), *virB11*(ORF0364), and *virD4 *(ORF0365). In addition, *R. africae *possessed a *traX *(ORF0816) and a split *fimD *(ORF0592/ORF0593/ORF0594) gene but lacked other Tra cluster genes found in *R. massiliae*, *R. felis*, *R. bellii *and *O. tsutsugamushi*, such as *traC *and *traG*_*F*_[15,28,29,31]. Therefore, the Tra cluster was mostly eliminated from the *R. africae*, and, following a "use it or lose it" scheme, this species probably did not need a *tra *gene-linked conjugation system. In addition, the pRA plasmid did not contain genes encoding proteins involved in conjugation.

Six transposase-encoding genes were identified in the chromosome, including one split into two ORFs (ORF0955/ORF0956) and one present as a remnant and two in the pRA plasmid, including one present as a fragment. This contrasts with the large expansion of transposases caused by gene duplications previously detected in *R. felis *and *R. bellii *[15,28].

### Common rickettsial gene set and phylogeny

**Figure 4:**
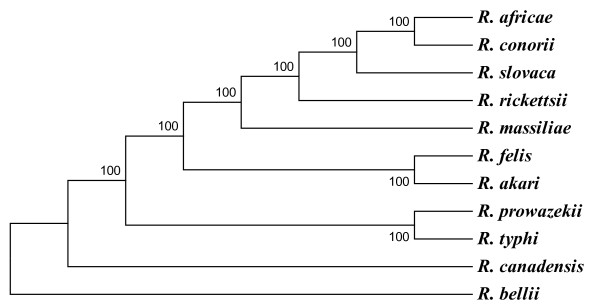
**Phylogenetic tree inferred from the comparison of 645 concatenated *Rickettsia *core protein-coding genes**. Similar organizations were obtained using both the maximum parsimony and neighbor joining methods. Bootstrap values are indicated at branch nodes.

When compared to eight other available rickettsial genomes, a total of 645 genes and 39 RNA-encoding genes of *R. africae *had orthologs in all genomes. In addition, another 32 *R. africae *genes had orthologs only in SFG rickettsiae and were either absent or remnant in TG rickettsiae. Consequently, we identified 645 genes as constituting the core gene set of all available rickettsial genomes and 700 ORFs as the core gene set of SFG rickettsiae. Following concatenation of the 645 core genes, a reliable phylogenetic organization (Figure 4) was obtained using three analysis methods that was consistent with previous phylogenetic studies of *Rickettsia *species [4,32-36].

In comparison with other *Rickettsia *genomes, *R. africae *had 242, 238 and 69 fewer genes than *R. bellii*, *R. felis *and *R massiliae*, respectively, but 279, 260, 52, 23, 17, and 15 more genes than *R. typhi*, *R. prowazekii*, *R. akari, R. rickettsii*, *R. sibirica*, and *R. conorii*, respectively. When comparing the numbers of degraded genes (split + remnants), *R. africae*, with 127 degraded genes, had a significantly less degraded genome (*P *< 10^-2^) than that of other spotted fever group rickettsiae including *R. akari *(176), *R. conorii *(196), *R. massiliae *(212), *R. rickettsii *(198) and *R. sibirica *(199) (Table 1). It had, however, significantly more degraded genes than *R. felis *(86, *P *< 10^-2^).

### Transcription of genes conserved in *R. africae *but absent from highly pathogenic species

**Figure 5:**
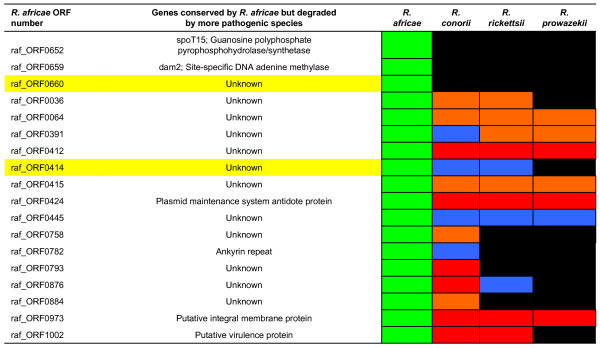
**Schematic representation of the genes conserved in *R. africae *but lost by highly pathogenic rickettsiae**. Genes highlighted in yellow are upregulated at 37°C. The state of a gene is represented by a small box colored in green (full-length), blue (pseudogene), red (fragment), orange (remnant) or black (absent). Gene numbers are indicated in the left column.

*R. africae *had 18 intact genes that were either absent or degraded in all three virulent species *R. conorii*, *R. rickettsii *and *R. prowazekii*. Of these, 12 encoded proteins of unknown functions (raf_ORF0036, raf_ORF0064, raf_ORF0391, raf_ORF0412, raf_ORF0414, raf_ORF0415, raf_ORF0445, raf_ORF0660, raf_ORF0758, raf_ORF0793, raf_ORF0876, and raf_ORF0884) (Figure 5) [see Additional file 4]. The remaining six genes encoded a plasmid maintenance system antidote protein (raf_ORF0424), the *spoT15 *gene (raf_ORF0652), a site-specific DNA adenine methylase (Dam2) (raf_ORF0659), an ankyrin repeat (raf_ORF0782), a putative integral membrane protein (raf_ORF0973), and a protein (RIG1002) exhibiting a high degree of amino acid sequence identity (>50%) with proteins of γ-proteobacteria classified within the COG3943 as putative virulence proteins. When investigating the transcription of these 18 genes in *R. africae *grown at 28, 32 and 37°C, we observed a significantly higher transcription level at 37°C than at lower temperatures for two genes, raf_ORF414 and raf_ORF660. The former gene contained a putative protease domain site, but the latter had no known function.

### The *R. africae *plasmid

The *R. africae *plasmid (Figure 2) is a new example of a plasmid in *Rickettsia *species, following those in *R. felis *[28], *R. massiliae *[29], *R. monacensis *[37], *R. helvetica, R. peacockii, R. amblyommii *and *R. hoogstraalii *[38]. This plasmid, named pRA, is smaller (12,377 bp) than those of *R. felis *(62,829 bp and 39,263 bp long, for pRF and pRFδ, respectively), *R. monacensis *(23,486 bp), and *R. massiliae *(15,286 bp). The pRA plasmid is predicted to contain 11 genes, 6 of which (54%) have homologs in public databases and are associated with functional attributes. These six genes encode for a chromosomal replication initiator DnaA-like protein (ORF1260), a site-specific recombinase (ORF1262), two contiguous transposases exhibiting 100% sequence similarity (ORF1263 and 1264) but with one (ORF1263) shorter than the other, the auto-transporter protein SCA12 (ORF1268), and a ParA-like plasmid stability protein (ORF1270). Five genes (ORFs 1260, 1263, 1264, 1269 and 1270) have orthologs in the *R. massiliae *plasmid, six have orthologs in the *R. felis *plasmids (ORF1260, 1263, 1264, 1268, 1269 and 1270), and three have orthologs in the *R. monacensis *plasmid (ORF1260, ORF1268, and ORF1270). The presence of two genes (ORF1260 and 1270) conserved in plasmids from four species suggests that these plasmids have a common origin. The presence of two almost identical successive transposases in *R. africae *matching a single gene in *R. massiliae *and *R. felis *suggests a duplication event in the former species. The pRA plasmid lacks heat shock protein-encoding genes found in other rickettsial plasmids. In contrast, ORF1262, a site-specific recombinase, is absent from other species. Its closest phylogenetic neighbour is a site-specific recombinase from *Magnetospirillum magnetotacticum*, a high G-C content α-proteobacterium living in aquatic environments [39]. The s*ca12 *gene (ORF) found intact in *R. africae *pRA was absent from the *R. massiliae *and *R. monacensis *plasmids and present but fragmented within *R. felis *pRF, but it was absent from pRFδ as well all other *Rickettsia *species.

As outlined by Baldridge *et al*. [38], the plasmid content of a *Rickettsia *species may vary according to the passage history of rickettsial strains. When estimating the prevalence of the plasmid among *R. africae *strains, we detected it in the 22 tested isolates from South Africa and in the 48 eschar biopsies from patients with ATBF contracted in the same country and in 20/32 *R. africae-*positive *Amblyomma *ticks [see Additional files 5 and 6]. Therefore, it appears from these results that, depending on the geographic location, the plasmid of *R. africae *may be unstable. Whether the plasmid has been lost by PCR-negative strains or cannot be amplified with the primers we used is as yet unknown. Such inter-strain differences in plasmid content were also observed in *R. felis *(Unpublished data).

### Stress response

Rickettsiae live intracellularly in both arthropod and mammal hosts. This implies that periods of tick starvation and feeding cause bacterial dormancy and multiplication following reactivation [40]. As a consequence, and despite their obligate intracellular location, rickettsiae may face, and thus have to adapt to, highly variable and extreme environmental conditions. Known as the stringent response, this bacterial adaptation to nutritional stress has been described to be mediated by the accumulation of guanosine nucleotides pppGpp (guanosine 3'-diphosphate 5'-triphosphate) and ppGpp (guanosine 3'-diphosphate 5'-diphosphate) [41]. Accordingly, the transcriptional analysis of *R. conorii *exposed to a nutrient deprivation was characterized by the up-regulation of *gmk *and of genes from the *spoT *family, suggesting a role for these nucleotides as effectors of the stringent response [42,43]. The *R. africae *genome exhibited eight *spoT *genes phylogenetically classified within two major clades [see Additional file 7]. The largest clade included *spoT *genes with hydrolase activity (1–10, 14, 15, 17–21), while the second included those with a synthetase domain. With eight genes, *R. africae *had more *spoT *genes than *R. rickettsii *(5 genes), *R. conorii *(4), *R. sibirica *(4), *R. akari *(7), *R. canadensis *(5), *R. typhi *(4) and *R. prowazekii *(1) but fewer genes than *R. felis *(14) and *R. bellii *(10) [see Additional file 8]. Altogether, our data suggest that *R. africae *is more regulated than more pathogenic species.

### Infection of mammal hosts

The *R. africae *genome encoded rOmpA (or Sca0) and rOmpB (or Sca5), two surface-exposed and immunodominant proteins belonging to the paralogous "surface cell antigen" (SCA) family and known in *Rickettsia *species to be responsible for antigenic differences between species [1] and to elicit an immune response in patients [44]. Experimental studies suggested that these two auto-transporter proteins could function as adhesins [10,11,45,46]. In addition, another eight SCA-encoding genes were found in the genome. These 10 genes were represented by 22 ORFs due to partial degradation of some of the paralogs [see Additional file 8]. Among the 17 SCA-encoding genes detected in *Rickettsia *species [47], *R. africae *had similar sets of conserved (*sca*0 – 2, 4 and 5), degraded (*sca*3, 8 – 10 and 13) and absent (*sca*6, 7, 11, 14 – 17) *sca *genes as *R. conorii *and *R. rickettsii*. In addition to these 10 SCA-encoding genes, *R. africae *exhibited a degraded *sca*9 gene and a complete *sca*12 gene carried by the pRA plasmid, only shared with *R. felis*, where it was also found partially degraded on the pRF plasmid. The *sca*12 genes from both species were grouped into a distinct cluster close to the *sca*1, 2 and 6 genes [see Additional file 9]. This result further supports a common origin of the pRA and pRF plasmids.

A proteomic approach recently allowed the identification of two paralogous proteins encoded by the genes RC1281-RC1282 and RP827-RP828, as putative adhesins Adr1 and Adr2. These proteins may be key actors for entry and infection in both *R. conorii *and *R. prowazekii *[11]. Both proteins are ubiquitously present within the *Rickettsia *genus [4]. Their presence within the *R. africae *genome (ORF1174 + ORF1175) [see Additional file 10] reinforces their suspected key role in rickettsial life.

**Figure 6:**
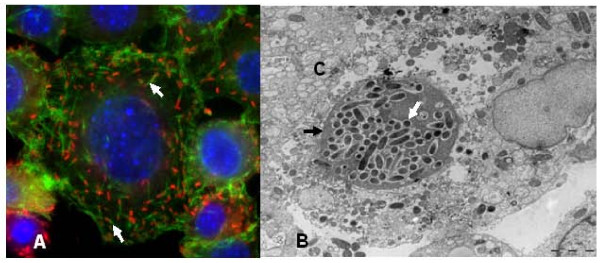
**Intracellular motility of *R. africae***. A) Actin tail formation by *R. africae*. L-929 cells were infected with *R. africae*, fixed and stained with fluorescent phalloidin (green) and a polyclonal antibody against *R. africae *and visualized using anti-rabbit-Alexa549 as a secondary antibody (red). The white arrows show actin tails. B)*R. africae *in the cytoplasm and nucleus of L-929 cells. C = cytoplasm; black arrow = nucleus; white arrows = *R. africae *bacilli. Transmission electron microscopy. Scale bar = 5 μm.

Both *pld *and *tlyC*, encoding phospholipase D [8] and hemolysin C [12], respectively, which play a role in phagosomal escape [13,48], were conserved in the *R. africae *genome (ORF1161 and ORF1039, respectively). This bacterium also exhibited genes encoding other proteins with membranolytic activity, including *tlyA *(hemolysin A) and *pat*1 (patatin-like phospholipase) [12,49]. As expected, the genome of *R. africae *has a *rickA *gene (ORF0824) orthologous to all rickettsial *rickA *genes and coding a protein activating the Arp2/3 complex, whose nucleation triggers actin polymerisation [50] [see Additional file 11]. The Rick A protein in *R. africae *is slightly different from those of other species, with a phenylalanine instead of a serine within the G-actin-binding site, an ENNIP [PS] motif repeated twice instead of four times in the central proline-rich region of the protein [see Additional file 11], and an aspartate and an isoleucine instead of an asparagine and an alanine or valine, respectively, in the carboxy-terminal region. Despite these differences, the RickA protein of *R. africae *appeared to be functional as demonstrated by its ability to polymerize actin and multiply intranuclearly (Figure 6).

Sixteen *vir *gene paralogs were found in the *R. africae *genome. Virulence genes of the *vir *family belong to the type IV secretion machinery, a system that allows the delivery of virulence factors from bacterial and eukaryotic host membranes to the cytoplasm of the host cell [51]. All 16 genes were found to be intact and common to all *Rickettsia *genomes with the exception of *virB*6-2 in *R. africae *and *virB*6-5 in *R. massiliae *[see Additional file 8]. In both species, these genes were split into two ORFs. Phylogenetic analysis of the *virB*6-2 gene distinguished clearly the SFG and TG and showed that the *R. africae *VirB6-2 protein is phylogenetically closer to that of *R. sibirica *[see Additional file 12].

### Clonality of *R. africae*

Of the 155 *Amblyomma *ticks tested, 139 (89.6%) were PCR-positive for *R. africae *[see Additional file 5]. Therefore, infection rates of *Amblyomma *ticks with *R. africae *may be higher than previously described [21,22,52], which suggests an extreme fitness of this rickettsia for its vector. In addition, such infection rates are the highest among *Rickettsia *species [see Additional file 13].

Using MST, PCR products of the expected sizes were obtained from the *dksA-xerC*, *mppA-purC *and *rpmE-*tRNA^fMet ^intergenic spacers from all tested specimens. Sequences obtained from these amplicons were in all cases identical to those previously obtained for *R. africae *[GenBank: DQ008280], [GenBank: DQ008301], and [GenBank: DQ008246], for the *dksA-xerC*, *mppA-purC *and *rpmE-*tRNA^fMet ^spacers, respectively). This is the first rickettsia demonstrated to be clonal. Other tested *Rickettsia *species, including *R. conorii *(31 MST genotypes out of 39 strains tested [53]), *R. massiliae *(2/7 [24]), *R. sibirica *(3/3 [24]), and *R. felis *(3/6 [24]), were significantly more genetically variable than *R. africae *(*p *< 10^-2 ^in all cases).

## Discussion

Using a comparative study of rickettsial genomes, we found that virulence in *Rickettsia *species is not correlated with acquisition of foreign DNA but may rather result from a reduction in regulation due to genome decay [6,23]. Comparative genomics sheds light on a much wider spectrum of virulence acquisition mechanisms in bacteria than initially thought [54]. Based on the examples of enterobacteria and staphylococci, gain in pathogenicity in bacteria was mainly thought to result from horizontal gene transfer, either directly or through mobile genetic elements [55,56]. However, a recent study of *Rickettsia *species associated with arthropods, insects, leeches and protists clearly demonstrated that horizontal gene transfer was a rare event within this genus [5]. In addition, genomic studies demonstrated that rickettsiae are undergoing genome decay, affecting in priority horizontally-acquired genes [57], and that there is no association between pathogenicity and acquisition of virulence markers [6]. In fact, the genome of the most virulent species, *R. prowazekii *[58], is a subset of the less pathogenic species *R. conorii *[23], thus highlighting a paradoxical relationship between smaller genome size and higher pathogenicity. Careful comparison of the *R. prowazekii *and *R. typhi *genomes also demonstrated that the former species, more pathogenic than the latter, had a more decayed genome despite a 12-kb insertion that likely resulted from a single genetic event [59].

When investigating the genomic characteristics associated with the milder virulence of *R. africae*, we first ruled out a potential role of the plasmid by the fact that it is unstable in this species. Then, we compared the gene contents of *R. africae *with *R. conorii*, *R. rickettsii*, and *R. prowazekii*, which exhibit a higher pathogenicity in humans and their arthropod hosts. We observed that *R. africae *showed no gene loss but had 18 genes fully conserved that were either absent or degraded in the other species (Figure 5). We speculated that, because *R. africae *had more intact genes than more virulent species, some of these genes may be involved in maintaining a low virulence level. Such a behavior may not be unique to rickettsiae. It was found that gene knockout resulted in increased virulence in *Mycoplasma*, *Streptococcus pyogenes*, and *Vibrio cholerae *[60-62]. In *M. ulcerans*, genome reduction was also linked to gain in virulence [63]. It emerges as a concept that virulence may be increased by gene loss [54]. We assume that a similar phenomenon may happen in rickettsiae, and that inactivation of some genes may deregulate the control of bacterial multiplication, in particular during the reactivation phenomenon following warming, thus enhancing pathogenesis.

Among the 18 putative candidate genes unique to *R. africae*, we identified only two genes (raf_ORF414 and raf_ORF660) that were significantly more transcribed at 37°C than at lower temperatures. Of these, one (raf_ORF414) encoded a protein that had a putative protease domain. A protease was previously shown in *Vibrio cholerae *to be a virulence repressor [60]. However, whether this differentially-transcribed protease plays a role in virulence repression in *R. africae *is as yet unknown. In contrast, the *spoT*15 gene (raf_ORF652) unique to *R. africae *was not upregulated, and this species retained another two *spoT *pseudogenes (raf_ORF653–654 and raf_ORF655–656) that were completely lost by other species. *SpoT *genes, effectors of the stringent response, were shown to play a major role in adaptation to stress in *R. conorii*, in particular when subjected to abrupt temperature variations similar to those occurring during a tick blood meal [42]. *R. africae*, however, has more *spoT *genes than *R. conorii *or *R. rickettsii *and does not show any modification of expression of its specific *spoT*15 gene during temperature variations. We speculate that higher regulation ability in *R. africae *is linked to lower pathogenicity.

In addition, when compared to other tick-borne *Rickettsia *species, *R. africae *exhibited several unique characteristics. First, this species is extremely successful and fit: it is highly adapted and harmless to its tick host, being efficiently transmitted both transtadially and transovarially in *Amblyomma *sp. ticks, which consequently act as efficient reservoirs [64]. In contrast, *R. rickettsii *[65,66] and *R. conorii *[67] have a negative effect on their tick vectors in experimental models. As a result, the prevalence of *R. africae *in its host ticks is higher than that of most other rickettsiae. Similarly, *R. africae *is less pathogenic for humans than other SFG species such as *R. conorii *and *R. rickettsii*, in particular because the infection is never lethal [17]. This observation was later supported by the demonstration that inoculation eschars in ATBF were histologically different from those in MSF [68]. In particular, in contrast with other SFG rickettsioses where eschars are characterized by perivascular infiltration of T cells and macrophages, with some B lymphocytes and few polymorphonuclears, the vasculitis in ATBF is made of a large infiltrate of neutrophils causing an extensive cutaneous inflammation and necrosis [see Additional file 14] [68]. Such a local reaction, in addition to the few *R. africae *cells detected in eschars [68], suggests that the bacterium replicated poorly in human tissues. Second, *R. africae *has significantly fewer degraded genes than other SFG species (*p *< 10^-2^), except *R. felis*. Specifically, this characteristic suggests that *R. africae *is undergoing a slower degradation process than other rickettsiae. Third, the identification of a single MST genotype among 102 strains suggested that *R. africae *was clonal [24,69]. This contrasted with the variable plasmid content of this species. Originally thought to be absent in *Rickettsia *species, plasmids have been detected in eight species to date [28,29,37,38], and their plasmid content may exhibit intraspecies variability. In *R. felis*, two plasmid forms have been sequenced [28], and Baldridge *et al*. found two plasmids in both *R. peacockii *and *R. amblyommii *[38]. In addition, these authors showed that *R. peacockii *lost its plasmids during long-term serial passages in cell culture [38]. In *R. africae*, the pRA plasmid may also be unstable, as shown by the absence of plasmid detection in 12/32 *Amblyomma *ticks tested. This plasmid encodes 11 ORFs, two of which are common to *R. felis*, *R. massiliae *and *R. monacensis *plasmids [see Additional file 1], which strongly suggests a common source for these mobile elements. We suspect that rickettsial plasmids and Tra clusters are vertically inherited but are apparently unstable and are currently degrading.

## Conclusion

Based on its genome and lifestyle, we suspect that the clonal *R. africae *is more regulated and more specifically adapted to its host and warm environment than other tick-associated rickettsiae. We speculate that losing this regulation, as observed in several intracellular pathogens, is a critical cause of virulence [6]. Further transcriptomic analysis of *R. africae *and other *Rickettsia *species grown at various temperatures is currently ongoing to identify putative other candidate genes involved in stress response.

## Methods

### Genome Sequencing

#### Bacterial purification and DNA extraction

In this study, we used *R. africae *ESF-5 strain, CSUR R15 (Collection de souches de l'Unité des Rickettsies, Marseille, France), which was isolated in an *Amblyomma variegatum *tick collected from cattle in the Shulu province of Ethiopia in 1966 [27]. *R. africae *was cultivated in Vero cells growing in MEM with 4% fetal bovine serum supplemented with 5 mM L-glutamine. Bacterial purification, DNA extraction and pulsed-filed gel electrophoresis were performed as described in Additional file 15 [see Additional file 15].

#### Shotgun sequencing of *R. africae *genome

Three shotgun genomic libraries were made by mechanical shearing of the DNA using a Hydroshear device (GeneMachine, San Carlos, CA, USA). Sequence blunt ends, to which the BstXI adaptator was linked, were obtained using the T4 DNA polymerase (New England Biolabs). Fragments of 3, 5, and 10 kb were separated on a preparative agarose gel (FMC, Rockland, ME, USA), extracted using the Qiaquick kit (Qiagen, Hilden, Germany), and ligated into a high copy-number vector pCDNA2.1 (Invitrogen, Carlsbad, CA, USA) for the two smaller inserts and into the low copy-number vector pCNS [28] for the largest inserts. Further details are available in Additional file 15 [see Additional file 15].

#### Annotation

We predicted protein-coding genes (ORFs) using SelfID as previously described [15]. Functional assignments for the ORFs were based on database searches using BLAST [70] against UniProt [71], NCBI/CDD [72], and SMART [73] databases. In most cases, we applied an E-value threshold of 0.001 for the database searches to retrieve homologues. Detailed analyses using multiple sequence alignments and phylogenetic reconstructions were carried out to assign putative functions to the ORFs, when needed. Orthologous gene relationships between *R. africae *and other *Rickettsia *species were approximated using the best reciprocal BLAST match criterion. The numbers of transposases, ankyrin/tetratricopeptide repeat-containing genes, and integrases were computed using RPS-BLAST with NCBI/CDD entries related to those domains with a 10^-5 ^E-value threshold. tRNA genes were identified using tRNAscan-SE [74]. To identify *Rickettsia *palindromic elements, we used hidden Markov models [75] based on the previously identified *Rickettsia *palindromic element sequences. ClustalW [76], T-coffee [77], and MUSCLE [78] were used to construct multiple sequence alignments. Toxin-antitoxin genes were identified using the Rasta-Bacteria software .

### Phylogenetic analysis

We based our analysis on the 645 complete orthologous genes found by Blast programmes in all *Rickettsia *genomes [70]. Subsequently, the amino acid sequences of these 645 proteins were concatenated for each genome and multiple alignment was performed using the Mafft software [79]. Gapped positions were removed. The maximum parsimony and neighbor joining trees were constructed using the MEGA 3.1 software [80].

### Clonal origin of *R. africae*

We examined *R. africae *within 155 *Amblyomma *sp. ticks and eggs from various geographical origins [see Additional file 5]. These included 80 adults (40 male and 40 female), 40 larvae, 15 nymphs and 20 eggs. PCR amplification of the *traD *gene was performed using the *R. africae*-specific primer pair traD-F (5'-caatgcttgatctatttggtag-3') and traD-R (5'-cttccttttctctaagctatgc-3') and the probe traD-probe (5'-FAM-ttatggtgctaactccatgcgtgatg-TAMRA-3'). The presence of the plasmid was estimated using the primer pair 1267F (5'-ccagccattaccgtaatcac-3') and 1267R (5'-tagtgccttatactcaagttc-3') and the probe 1267-probe (5'-FAM-gcagaaagtgattaaggcgatcagctg-TAMRA-3') that is able to detect ORF 1267 encoding a protein of unknown function specific to the plasmid. The presence of the plasmid was examined in 22 strains obtained from patients who contracted the disease in South Africa and maintained in the CSUR [see Additional file 6], in PCR-positive eschar biopsies from another 48 patients who developed ATBF following a trip to South Africa, and in 32 *Amblyomma *sp. ticks found positive for *R. africae*, using the above-described PCR assay [see Additional file 5]. To evaluate the genetic diversity of *R. africae*, we used the multi-spacer typing (MST) method as previously described [53]. This method has been described as the most discriminatory genotyping tool at the intraspecies level in *Rickettsia *sp. [53]. We applied this method to the aforementioned 22 human *R. africae *strains, 48 eschar biopsies, and 32 *Amblyomma *sp. ticks from Sudan (3), Madagascar (3), Mali (3), Niger (6), Central African Republic (6), Ivory Coast (3), Guadeloupe (4), Martinique (2), and S^t ^Kitts and Nevis (2) [see Additional file 5]. The obtained sequences were compared to those available in GenBank, and the MST genotypes were determined as previously described [53].

### Transcription of genes conserved in *R. africae *but absent from highly pathogenic species

To evaluate the transcription of the 18 genes conserved by *R. africae *and degraded in highly pathogenic species, we designed specific primer pairs and probes for each gene and tested the transcription of these genes by RT-PCR on RNA extracted from *R. africae*- infected Vero cells cultivated at 32 and then at 37°C and in XTC cells at 28 and 32°C. Experimental protocols are detailed in Additional file 15 [see Additional file 15].

## Authors' contributions

PEF and DR designed the study, drafted the manuscript, and gave final approval of the submitted version; KE, QL, CR, BG, PR, CR, PP, and SA performed experiments, drafted the manuscript and gave final approval of the submitted version.

## Supplementary Material

Additional file 1 **Gene content of the *R. africae *plasmid. **GenBank accession number sare indicated in square brackets. The Table includes a comparison of rickettsial plasmid contents. Click here for file

Additional file 2 ***R. africae *gene content**. The Table includes the gene content of the *R. africae *genome. Click here for file

Additional file 3 **Inversion observed by alignment of the *R. africae *(up) and *R. conorii *(down) genomes**. The Figure shows an alignment of the *R. conorii *and *R. africae *genomes. Click here for file

Additional file 4 **Schematic representation of the genes diversely conserved in *R. africae *in comparison with highly pathogenic rickettsiae. **The state of a gene is represented by a small box colored in green (full-length), blue (pseudogene), red (fragment), orange (remnant) or black (absent).Gene numbers are indicated in the left column. The Figure shows the gene distribution in *R. africae *by comparison with highly pathogenic rickettsiae. Click here for file

Additional file 5 **PCR-detection of *R. africae *and in *Amblyomma *ticks. **Results are indicated as number of ticks positive/number tested. The Table includes the results from PCR detection of the *R. africae *chromosome and plasmid in ticks. Click here for file

Additional file 6 ***Rickettsia africae *strains used in this study**. The table lists all *R. africae *strains used in this study. Click here for file

Additional file 7 **Phylogenetic tree showing the organization of *spoT *genes in *Rickettsia *species. **Phylogenetic relationships were inferred from aligned sequences using the Mega3.1 software with the Neighbor-Joining method. Bootstrap values are indicated at the nodes. The Figure is a phylogenetic tree showing the organization of *spoT *genes in *Rickettsia *species. Click here for file

Additional file 8 ***R. africae *ORFs compared to other available *Rickettsia *genomes**. The table details the distribution of *R. africae *ORF in other rickettsial genomes. Click here for file

Additional file 9 **Phylogenetic tree showing the organization of *sca *genes in *Rickettsia *species. **Phylogenetic relationships were inferred from aligned sequences using the Mega3.1 software with the Neighbor-Joining method. Bootstrap values are indicated at the nodes. The Figure is a phylogenetic tree showing the organization of *sca *genes in *Rickettsia *species. Click here for file

Additional file 10 **Phylogenetic tree showing the organization of *adr *genes in *Rickettsia *species. **Phylogenetic relationships were inferred from aligned sequences using the Mega3.1 software with the Neighbor-Joining method. Bootstrap values are indicated at the nodes. The Figure is a phylogenetic tree showing the organization of *adr *genes in *Rickettsia *species. Click here for file

Additional file 11 **Features of RickA repeat proline-rich motif in *R. africae *and other SFG rickettsiae. **The motif " [EDGKQG]- [NS]-N- [IV]- [PSLTR](0,28)" was used to extract these repeats using a PatternMatchingtool. The table details RickA repeat proline-rich motifs in *R. africae *and other SFG rickettsiae. Click here for file

Additional file 12 **Phylogenetic tree showing the organization of *virB*6-2 genes in *Rickettsia *species. **Phylogenetic relationships were inferred from aligned sequences using the Mega3.1 software with the Neighbor-Joining method. Bootstrap values are indicated at the nodes. The Figure is a phylogenetic tree showing the organization of *virB*6-2 genes in *Rickettsia *species. Click here for file

Additional file 13 **Comparison of epidemiological and clinical characteristics of *Rickettsia *species**. The table includes data about the epidemiological and clinical characteristics of *Rickettsia *species. Click here for file

Additional file 14 **Immunohistochemical detection of *R. africae *(arrows) in the inoculation eschar of a patient with ATBF (monoclonal rabbit anti-*R. africae *antibody used at a dilution of 1:1,000 and hematoxylin counterstain; original magnification ×250)**. The Figure shows the presence of *R. africae *in the inoculation eschar of a patient with ATBF, revealed by immunohistochemistry. Click here for file

Additional file 15 **Supplementary material and methods**. The data provided include detailed material and methods that were used for the genome sequencing and sequence analysis of *R. africae*. Click here for file
