# Supplementary material for: Analysis of the Rickettsia africae genome reveals that virulence acquisition in Rickettsia species may be explained by genome reduction
Source: BMC Genomics. 2009 Apr 20;10:166. doi: 10.1186/1471-2164-10-166 (PMC2694212; doi:10.1186/1471-2164-10-166)
Supplement: Additional file 1 — Gene content of the R. africae plasmid. GenBank accession number sare indicated in square brackets. The Table includes a comparison of rickettsial plasmid contents. [file 1471-2164-10-166-S1.doc]

| ***R. africae* ORF number** | **Gene**  **name** | **Functional annotation** | ***R. felis***  **pRF**  [GenBank:NC_007110] | ***R. massiliae***  **pRMA**  [GenBank:NC_009897] | | R. monacensis **pRM**  [GenBank:EF564599] |
| --- | --- | --- | --- | --- | --- | --- |
|  |  |  |
| **RAF_ORF1260** |  | Unknown  (DnaA-like) | pRF05 |  | + | pRM16 |
| **RAF_ORF1261** |  | Unknown | - |  | - | - |
| **RAF_ORF1262** |  | Specific-site recombinase,  DNA invertase Pin-like protein | - |  | - | - |
| **RAF_ORF1263** | *tnp* | Transposase and inactivated derivative | pRF61 |  | RMA-p06 | - |
| **RAF_ORF1264** | *tnp* | Transposase and inactivated derivative | pRF61 |  | RMA-p06 | - |
| **RAF_ORF1265** |  | Unknown | - |  | - | - |
| **RAF_ORF1266** |  | Unknown | - |  | - | - |
| **RAF_ORF1267** |  | Unknown | - |  | - | - |
| **RAF_ORF1268** | ***sca12*** | Cell surface antigen protein | pRF25 |  | - | pRM21 |
| **RAF_ORF1269** |  | Unknown | pRF29 |  | + | - |
| **Raf_ORF1270** | ***parA2*** | Plasmid stability protein ParA | pRF23 |  | RMA-p13 | pRM18 |
