# Supplementary material for: Analysis of the Rickettsia africae genome reveals that virulence acquisition in Rickettsia species may be explained by genome reduction
Source: BMC Genomics. 2009 Apr 20;10:166. doi: 10.1186/1471-2164-10-166 (PMC2694212; doi:10.1186/1471-2164-10-166)
Supplement: Additional file 2 — R. africae gene content. The Table includes the gene content of the R. africae genome. [file 1471-2164-10-166-S2.doc]

| **Functional**  **category** | **Gene name** | **Product** | **Intact** | **Split** | | | **Fragment** |
| --- | --- | --- | --- | --- | --- | --- | --- |
|  |  |  |  | **Nbr1.** | | **Nbr2.** |  |
|  |  | (Di)nucleoside polyphosphate hydrolase-like protein | 1 |  |  | |  |
|  |  | 5-Formyltetrahydrofolate cyclo-ligase [EC:6.3.3.2] | 1 |  |  | |  |
|  |  | AAA+ superfamily protein |  |  |  | | 1 |
|  | *aas* | 2-acylglycerophosphoethanolamine acyltransferase [EC:6.2.1.20] | 1 |  |  | |  |
|  |  | aatA; Aspartate aminotransferase A [EC:2.6.1.1] | 1 |  |  | |  |
|  |  | ABC transporter ATP-binding protein | 1 |  |  | | 2 |
|  |  | ABC transporter permease protein | 2 |  |  | |  |
|  |  | ABC transporter substrate binding protein | 1 |  |  | |  |
|  |  | ABC-type transport system, periplasmic component | 2 |  |  | |  |
|  |  | ABC-type transporter related to toluene tolerance | 1 |  |  | |  |
|  | *abcT* | ABC transporter ATP-binding protein | 2 |  |  | |  |
|  | *abcT3* | Multidrug resistance ABC transporter ATP-binding protein | 1 |  |  | |  |
|  | *accC* | Acetyl-CoA carboxylase, biotin carboxylase [EC:6.4.1.2] | 1 |  |  | |  |
|  |  | Acetyltransferases | 1 | 1 | 2 | | 1 |
|  |  | Acetyltransferase GNAT family protein | 1 |  |  | |  |
|  | *ackA* | Acetate kinase [EC:2.7.2.1] |  | 1 | 2 | |  |
|  | *acnA* | Aconitate hydratase [EC:4.2.1.3] | 1 |  |  | |  |
|  | *aco1* | Acyl-CoA desaturase 1 | 1 |  |  | |  |
|  | *acpP* | Acyl carrier protein | 1 |  |  | |  |
|  | *acpS* | Holo-[acyl carrier protein] synthase [EC:2.7.8.7] | 1 |  |  | |  |
|  | *acrF* | Hydrophobe/amphiphile efflux-1 (HAE1) family protein | 1 |  |  | |  |
|  |  | Acylamino-acid-releasing enzyme | 1 |  |  | |  |
|  | *addA* | Erythrocyte adducin alpha subunit | 1 |  |  | |  |
|  | *adk* | Adenylate kinase [EC:2.7.4.3] | 1 |  |  | |  |
|  | *alaS* | Alanyl-tRNA synthetase [EC:6.1.1.7] | 1 |  |  | |  |
|  |  | Alpha-(1,3)-fucosyltransferase |  | 1 | 2 | |  |
|  | *alr* | Alanine racemase [EC:5.1.1.1] | 1 |  |  | |  |
|  |  | Amino acid permeases |  |  |  | | 1 |
|  |  | Aminodeoxychorismate lyase | 1 |  |  | |  |
|  | *ampD* | Negative regulator of beta-lactamase expression | 2 |  |  | |  |
|  | *ampG* | AmpG | 4 |  |  | |  |
|  |  | Ankyrin repeat | 4 | 1 | 2 | | 4 |
|  |  | Antitoxin of toxin-antitoxin (TA) system | 4 |  |  | |  |
|  | *aprD* | Alkaline protease secretion ATP-binding protein AprD | 1 |  |  | |  |
|  | *argB* | Acetylglutamate kinase [EC:2.7.2.8] | 1 |  |  | |  |
|  | *argS* | Arginyl-tRNA synthetase [EC:6.1.1.19] | 1 |  |  | |  |
|  | *asd* | Aspartate-semialdehyde dehydrogenase [EC:1.2.1.11] | 1 |  |  | |  |
|  | *asmA* | Outer membrane assembly protein | 1 |  |  | |  |
|  | *aspS* | Aspartyl-tRNA synthetase [EC:6.1.1.12] | 1 |  |  | |  |
|  | *atm1* | Multidrug resistance protein |  | 1 | 3 | |  |
|  |  | ATP-dependent helicase | 1 |  |  | |  |
|  | *atp* | ATP synthase A, B, C, D, E, F, G, H, X | 9 |  |  | |  |
|  | *atrC1* | Cationic amino acid transporter-1 | 1 |  |  | |  |
|  |  | AtsE-like protein | 1 |  |  | |  |
|  | *barA* | Histidine kinase sensor protein [EC:2.7.3.-] | 1 |  |  | |  |
|  | *bcp* | Bacterioferritin comigratory protein | 1 |  |  | |  |
|  | *bcr* | MFS-type bicyclomycin resistance protein | 1 | 1 | 3 | |  |
|  |  | Beta-glucosidase [EC:3.2.1.21] | 1 |  |  | |  |
|  | *bioY2* | BioY family protein | 1 |  |  | |  |
|  | *birA* | Biotin-(acetyl-CoA carboxylase) ligase [EC:6.3.4.15] | 1 |  |  | |  |
|  | *blaD* | Class D beta-lactamase |  | 1 | 2 | |  |
|  | *bolA* | BolA-like protein | 2 |  |  | |  |
|  | *bpl1* | Biotin-protein ligase [EC:6.3.4.-] | 1 |  |  | |  |
|  |  | Capsular polysaccharide biosynthesis protein |  | 1 | 2 | |  |
|  | *ccm* | Heme exporter protein A, B, C | 3 |  |  | |  |
|  | *ccm* | Cytochrome c-type biogenesis protein ccmE, ccmF | 2 |  |  | |  |
|  | *cdsA* | Phosphatidate cytidylyltransferase [EC:2.7.7.41] | 1 |  |  | |  |
|  |  | Cell filamentation protein Fic | 1 |  |  | |  |
|  | *chaB* | Cation transport regulator ChaB |  |  |  | | 1 |
|  |  | Chromosome partitioning protein-like protein | 1 |  |  | |  |
|  | *clpB* | ClpB | 1 |  |  | |  |
|  | *clp* | ATP-dependent Clp protease clpP, clpX | 2 |  |  | |  |
|  | *cmcI* | Cephalosporin hydroxylase |  |  |  | | 1 |
|  | *cmk* | Cytidylate kinase [EC:2.7.4.14] | 1 |  |  | |  |
|  | *coaE* | Dephospho-CoA kinase [EC:2.7.1.24] | 1 |  |  | |  |
|  |  | ComEC/Rec2 family protein |  | 1 | 2 | | 1 |
|  | *comF* | Competence protein F comF1, comF2 | 2 |  |  | |  |
|  | *comJ* | Protein involved in catabolism of external DNA (SPLIT GENE) |  | 1 | 2 | |  |
|  | *comL* | DNA uptake lipoprotein | 1 |  |  | |  |
|  | *coq7* | Ubiquinone biosynthesis protein coq7 | 1 |  |  | |  |
|  | *corA* | Magnesium and cobalt transport protein CorA | 1 |  |  | |  |
|  | *cox* | Cytochrome c oxidase cox11coxA, coxB, coxC, coxW | 5 |  |  | |  |
|  | *cspA* | Cold shock-like protein | 1 |  |  | |  |
|  | *ctp* | Carboxyl-terminal protease [EC:3.4.21.-] | 1 |  |  | |  |
|  | *cutA* | Periplasmic divalent cation tolerance protein | 1 |  |  | |  |
|  | *cutE* | Apolipoprotein N-acyltransferase [EC:2.3.1.-] | 1 |  |  | |  |
|  | *cyaY* | CyaY | 1 |  |  | |  |
|  | *cycM* | Cytochrome c | 1 |  |  | |  |
|  | *cyd* | Cytochrome d ubiquinol oxidase cydA, cydB | 2 |  |  | |  |
|  | *cyoB* | ctaB; Protoheme IX farnesyltransferase [EC:2.5.1.-] | 1 |  |  | |  |
|  | *cysQ* | 3'(2'),5'-bisphosphate nucleotidase [EC:3.1.3.7] | 1 |  |  | |  |
|  | *cysS* | Cysteinyl-tRNA synthetase [EC:6.1.1.16] | 1 |  |  | |  |
|  | *cytB* | Cytochrome b561 family protein (SPLIT GENE) |  | 1 | 2 | |  |
|  |  | Cytosine deaminase [EC:3.5.4.1] | 1 |  |  | |  |
|  | *czcR* | Transcriptional activator protein CzcR | 1 |  |  | |  |
|  |  | D-alanyl-D-alanine dipeptidase |  |  |  | | 1 |
|  | *dacF* | Penicillin-binding protein dacF precursor [EC:3.4.16.4] | 1 |  |  | |  |
|  | *dam* | Site-specific DNA methylase dam1, dam2 | 2 |  |  | |  |
|  |  | DAP2 dipeptidyl aminopeptidase/acylaminoacyl-peptidase-like protein | 1 |  |  | |  |
|  | *dap* | Dihydrodipicolinate synthase dapA, dapB | 2 |  |  | |  |
|  | *dapD* | 2,3,4,5-tetrahydropyridine-2-carboxylate N-succinyltransferase [EC:2.3.1.117] | 1 |  |  | |  |
|  | *dapE* | Succinyl-diaminopimelate desuccinylase [EC:3.5.1.18] | 1 |  |  | |  |
|  | *dapF* | Diaminopimelate epimerase [EC:5.1.1.7] | 1 |  |  | |  |
|  | *dat* | Methylated-DNA--protein-cysteine methyltransferase [EC:2.1.1.63] | 1 |  |  | |  |
|  | *dcd* | Deoxycytidine triphosphate deaminase [EC:3.5.4.13] | 1 |  |  | |  |
|  | *ddlB* | D-alanine--D-alanine ligase [EC:6.3.2.4] | 1 |  |  | |  |
|  | *def* | Polypeptide deformylase def1, def2, def3 | 3 |  |  | |  |
|  | *dgt* | Deoxyguanosinetriphosphate triphosphohydrolase [EC:3.1.5.1] | 1 |  |  | |  |
|  | *dinJ* | DNA-damage-inducible protein J | 1 |  |  | |  |
|  |  | dksA; DnaK suppressor-like protein |  |  |  | |  |
|  |  | DnaA-like protein |  |  |  | |  |
|  |  | dnaA; Chromosomal replication initiator protein DnaA |  |  |  | |  |
|  |  | dnaB; Replicative DNA helicase [EC:3.6.1.-] |  |  |  | |  |
|  |  | dnaE; DNA polymerase III alpha chain [EC:2.7.7.7] |  |  |  | |  |
|  |  | dnaG; DNA primase [EC:2.7.7.-] |  |  |  | |  |
|  |  | dnaJ; DnaJ |  |  |  | |  |
|  |  | dnaK; DnaK |  |  |  | |  |
|  |  | dnaN; DNA polymerase III beta chain [EC:2.7.7.7] |  |  |  | |  |
|  |  | dnaQ; DNA polymerase III epsilon chain [EC:2.7.7.7] |  |  |  | |  |
|  |  | dnaX; DNA polymerase III gamma and tau chains [EC:2.7.7.7] |  |  |  | |  |
|  | *dsb* | Thiol:disulfide interchange protein dsbA, dsbB, dsbG | 3 |  |  | |  |
|  |  | dTDP-4-dehydrorhamnose reductase [EC:1.1.1.133] | 1 |  |  | |  |
|  | *dut* | Deoxyuridine 5'-triphosphate nucleotidohydrolase [EC:3.6.1.23] | 1 |  |  | |  |
|  |  | EAL domain containing protein | 1 |  |  | |  |
|  | *ecoT* | Ecotin precursor | 1 |  |  | |  |
|  |  | Efflux transporter, RND family, MFP subunit | 1 |  |  | |  |
|  | *efp* | Translation elongation factor EF-P | 1 |  |  | |  |
|  | *emr* | Multidrug resistance proteins emrA, emrB | 2 |  |  | |  |
|  | *envZ* | Osmolarity sensor protein EnvZ [EC:2.7.3.-] | 1 |  |  | |  |
|  | *era* | GTP-binding protein Era | 1 |  |  | |  |
|  | *exoC* | Phosphomannomutase [EC:5.4.2.8] | 1 |  |  | |  |
|  | *exsB* | Trans-regulatory protein ExsB | 1 |  |  | |  |
|  |  | fabD; Malonyl CoA-acyl carrier protein transacylase [EC:2.3.1.39] |  |  |  | |  |
|  |  | fabF; 3-oxoacyl-[acyl carrier protein] synthase II [EC:2.3.1.41] |  |  |  | |  |
|  |  | fabG; 3-oxoacyl reductase [EC:1.1.1.100] |  |  |  | |  |
|  |  | fabH; 3-oxoacyl-[acyl carrier protein] synthase III [EC:2.3.1.41] |  |  |  | |  |
|  |  | fabI; Enoyl-[acyl carrier protein] reductase [EC:1.3.1.9] |  |  |  | |  |
|  |  | fabZ; (3R)-hydroxymyristoyl-[acyl carrier protein] dehydratase [EC:4.2.1.-] |  |  |  | |  |
|  | *fadB* | 3-hydroxyacyl-CoA dehydrogenase FadB |  | 1 | 5 | |  |
|  | *fbcH* | Cytochrome c1, heme protein precursor | 1 |  |  | |  |
|  | *fdx* | Ferredoxin, fdxA, fdxB | 2 |  |  | |  |
|  | *ffh* | Signal recognition particle protein | 1 |  |  | |  |
|  | *fimD* | papC; P pilus assembly, fimbrial Usher protein |  | 1 | 3 | |  |
|  |  | Flavoprotein oxygenase DIM6/NTAB family protein | 1 |  |  | |  |
|  | *fmt* | Methionyl-tRNA formyltransferase [EC:2.1.2.9] | 1 |  |  | |  |
|  | *fni* | Isopentenyl-diphosphate delta-isomerase [EC:5.3.3.2] | 1 |  |  | |  |
|  |  | folA; Dihydrofolate reductase [EC:1.5.1.3] |  |  |  | |  |
|  |  | folC; Folylpolyglutamate synthase [EC:6.3.2.17] |  |  |  | |  |
|  |  | folD; Methylenetetrahydrofolate dehydrogenase [EC:1.5.1.5 3.5.4.9] |  |  |  | |  |
|  |  | folE; GTP cyclohydrolase I [EC:3.5.4.16] |  |  |  | |  |
|  |  | folKP; Folate synthesis bifunctional protein [EC:2.7.6.3] [EC:2.5.1.15] (SPLIT GENE) |  |  |  | |  |
|  |  | folKP; Folate synthesis bifunctional protein [EC:2.7.6.3] [EC:2.5.1.15] (SPLIT GENE) |  |  |  | |  |
|  |  | folKP; Folate synthesis bifunctional protein [EC:2.7.6.3] [EC:2.5.1.15] (SPLIT GENE) |  |  |  | |  |
|  |  | FTR1 family protein | 1 |  |  | |  |
|  |  | ftsA; Cell division protein ftsA |  |  |  | |  |
|  |  | ftsH; ATP-dependent metalloprotease FtsH |  |  |  | |  |
|  |  | ftsK; Cell division protein FtsK |  |  |  | |  |
|  |  | ftsL; Cell division protein FtsL |  |  |  | |  |
|  |  | ftsQ; Cell division protein ftsQ |  |  |  | |  |
|  |  | ftsW; Cell division protein ftsW |  |  |  | |  |
|  |  | ftsY; Signal recognition particle-docking protein FtsY |  |  |  | |  |
|  |  | ftsZ; Cell division protein ftsZ |  |  |  | |  |
|  | *fumC* | Fumarate hydratase [EC:4.2.1.2] | 1 |  |  | |  |
|  | *fusA* | Elongation factor EF-G | 1 |  |  | |  |
|  | *gabD* | Succinate semialdehyde dehydrogenase [EC:1.2.1.16] | 1 |  |  | |  |
|  | *gat* | Glutamyl-tRNA(Gln) amidotransferase gatA, gatB, gatC | 3 |  |  | |  |
|  | *gcp* | Sialoglycoprotease [EC:3.4.24.57] | 1 |  |  | |  |
|  | *gidA* | Glucose-inhibited division protein A | 1 |  |  | |  |
|  | *glmU* | UDP-N-acetylglucosamine pyrophosphorylase [EC:2.7.7.23] | 1 |  |  | |  |
|  | *glnA* | Glutamine synthetase [EC:6.3.1.2] | 1 |  |  | |  |
|  | *glnQ* | Glutamine ABC transporter ATP-binding protein | 1 |  |  | |  |
|  | *gltA* | Citrate synthase I [EC:4.1.3.7] | 1 |  |  | |  |
|  | *gltD* | NADPH-dependent glutamate synthase beta chain and related oxidoreductases | 1 |  |  | |  |
|  | *gltP* | Na+/H+-dicarboxylate symporters | 1 |  |  | |  |
|  | *gltX* | Glutamyl-tRNA synthetase [EC:6.1.1.17] gltX1 gltX2 | 2 |  |  | |  |
|  |  | Glutathione S-transferase [EC:2.5.1.18] | 1 |  |  | |  |
|  | *glyA* | Glycine/serine hydroxymethyltransferase [EC:2.1.2.1] | 1 |  |  | |  |
|  |  | Glycosyltransferase | 2 | 1 | 3 | |  |
|  |  | Glycosyltransferase involved in LPS biosynthesis (FRAGMENT) | 1 |  |  | |  |
|  |  | Glycosyltransferase [EC:2.4.1.-], two domains | 1 |  |  | |  |
|  | *gly* | Glycyl-tRNA synthetase [EC:6.1.1.14] glyQ, glyS | 2 |  |  | |  |
|  | *gmk* | Guanylate kinase [EC:2.7.4.8] | 1 |  |  | |  |
|  | *gppA* | Guanosine pentaphosphate phosphohydrolase [EC:3.6.1.11 3.6.1.40] | 1 |  |  | |  |
|  | *gpsA* | Glycerol-3-phosphate dehydrogenase [EC:1.1.1.94] | 1 |  |  | |  |
|  | *greA* | Transcription elongation factor GreA | 1 |  |  | |  |
|  | *groE* | chaperonin groEL (60 kD), groES (10 kD) | 2 |  |  | |  |
|  | *grpE* | GrpE protein | 1 |  |  | |  |
|  | *grxC1*  *grxC2* | Glutaredoxin, GrxC family  Glutaredoxin-like protein grla | 2 |  |  | |  |
|  |  |  |  |  |  |  |  |
|  |  | GTP cyclohydrolase I | 1 |  |  | |  |
|  |  | GTP-binding protein | 1 |  |  | |  |
|  | *gyrA*  *gyrB* | DNA gyrase subunit A [EC:5.99.1.3]  DNA gyrase subunit B [EC:5.99.1.3] | 2 |  |  | |  |
|  |  |  |  |  |  |  |  |
|  |  | HAD-superfamily subfamily IIA hydrolase | 1 |  |  | |  |
|  |  | Heat shock protease [EC:3.4.21.-] | 1 |  |  | |  |
|  | *hemA*  *hemB*  *hemC*  *hemE*  *hemF*  *hemK*  *HemY* | 5-aminolevulinic acid synthase [EC:2.3.1.37]  Delta-aminolevulinic acid dehydratase [EC:4.2.1.24]  Porphobilinogen deaminase [EC:4.3.1.8]  Uroporphyrinogen decarboxylase [EC:4.1.1.37]  Coproporphyrinogen III oxidase precursor [EC:1.3.3.3]  Methylase of polypeptide chain release factors  Hemolysin-like protein  HemY-like protein | 8 |  |  | |  |
|  |  |  |  |  |  |  |  |
|  |  |  |  |  |  |  |  |
|  |  |  |  |  |  |  |  |
|  |  |  |  |  |  |  |  |
|  |  |  |  |  |  |  |  |
|  |  |  |  |  |  |  |  |
|  |  |  |  |  |  |  |  |
|  | *hflC1*  *hflC2*  *hflK* | Membrane protease subunit, stomatin/prohibitin-like protein  Membrane protease subunit, stomatin/prohibitin-like protein  Protease activity modulator HflK | 3 |  |  | |  |
|  |  |  |  |  |  |  |  |
|  |  |  |  |  |  |  |  |
|  |  | HicB-like protein | 1 |  |  | |  |
|  | *himA*  *himD* | Integration host factor alpha-subunit  Integration host factor beta-subunit | 2 |  |  | |  |
|  |  |  |  |  |  |  |  |
|  | *hisS* | Histidyl-tRNA synthetase [EC:6.1.1.21] | 1 |  |  | |  |
|  | *hlpA* | Outer membrane protein | 1 |  |  | |  |
|  |  | HlyD family secretion protein | 1 |  |  | |  |
|  | *holA*  *holB*  *holC* | DNA polymerase III, delta subunit  DNA polymerase III delta subunit [EC:2.7.7.7]  DNA polymerase III chi subunit HolC | 3 |  |  | |  |
|  |  |  |  |  |  |  |  |
|  |  |  |  |  |  |  |  |
|  | *hscA*  *hscB* | Heat shock protein hscA  Co-chaperone Hsc20 | 2 |  |  | |  |
|  |  |  |  |  |  |  |  |
|  | *hslU*  *hslV* | Heat shock protein HslVU, ATPase subunit HslU  Heat shock protein HslV [EC:3.4.99.-] | 2 |  |  | |  |
|  |  |  |  |  |  |  |  |
|  | *hspC1* | Small heat shock protein | 1 |  |  | |  |
|  | *htpG* | Heat shock protein htpG | 1 |  |  | |  |
|  | *htrA* | Periplasmic serine protease [EC:3.4.21.-] | 1 |  |  | |  |
|  | *htrB* | Lipid-A biosynthesis lauroyl acyltransferase [EC:2.3.1.-] | 1 |  |  | |  |
|  | *hupA* | DNA-binding protein HU |  |  |  | |  |
|  | *icd* | Isocitrate dehydrogenase, NADP-dependent [EC:1.1.1.42] | 1 |  |  | |  |
|  | *ileS* | Isoleucyl-tRNA synthetase [EC:6.1.1.5] | 1 |  |  | |  |
|  | *ilvE* | Branched-chain amino acid aminotransferase [EC:2.6.1.42] | 1 |  |  | |  |
|  | *imp* | TRAP-type uncharacterized transport system, periplasmic component | 1 |  |  | |  |
|  | *infA*  *infB*  *infC* | Translation initiation factor IF-1  Translation initiation factor IF-2  Translation initiation factor IF-3 | 3 |  |  | |  |
|  |  |  |  |  |  |  |  |
|  |  |  |  |  |  |  |  |
|  |  | Integral membrane protein, interacts with FtsH | 1 |  |  | |  |
|  |  | Iojap-related protein | 1 |  |  | |  |
|  | *iscA1*  *iscA2*  *iscR*  *iscS*  *iscU* | Iron-sulfur cluster assembly accessory protein  Iron-sulfur cluster assembly accessory protein  Iron-sulfur cluster assembly transcription factor IscR  Cysteine desulfurase IscS [EC:4.4.1.-]  FeS cluster assembly scaffold IscU | 5 |  |  | |  |
|  | *ispB*  *ispZ* | Octaprenyl-diphosphate synthase [EC:2.5.1.-]  Intracellular septation protein A | 2 |  |  | |  |
|  | *kdsA*  *kdsB* | 3-deoxy-8-phosphooctulonate synthase [EC:2.5.1.55]  3-deoxy-manno-octulosonate cytidylyltransferase [EC:2.7.7.38] | 2 |  |  | |  |
|  |  |  |  |  |  |  |  |
|  | *kdtA* | 3-deoxy-D-manno-octulosonic-acid transferase [EC:2.-.-.-] | 1 |  |  | |  |
|  | *kefB* | Glutathione-regulated potassium-efflux system protein KefB | 1 |  |  | |  |
|  | *kpsF* | KpsF | 1 |  |  | |  |
|  | *ksgA* | Dimethyladenosine transferase [EC:2.1.1.-] | 1 |  |  | |  |
|  | *lacA* | Ribose-5-phosphate isomerase [EC:5.3.1.6] | 1 |  |  | |  |
|  |  | Large extracellular alpha-helical protein | 1 |  |  | |  |
|  | *lepA*  *lepB* | GTP-binding protein LepA  Signal peptidase I [EC:3.4.21.89] | 2 |  |  | |  |
|  |  |  |  |  |  |  |  |
|  |  | Leucine-rich repeats (LRRs), ribonuclease inhibitor (RI)-like subfamily protein |  |  |  | | 1 |
|  | *leuS* | Leucyl-tRNA synthetase [EC:6.1.1.4] | 1 |  |  | |  |
|  | *lgt* | Prolipoprotein diacylglyceryl transferase [EC:2.4.99.-] | 1 |  |  | |  |
|  | *lgtD*  *lgtF* | Glycosyl transferase  Beta 1,4 glucosyltransferase | 2 |  |  | |  |
|  |  |  |  |  |  |  |  |
|  | *ligA* | DNA ligase, NAD-dependent [EC:6.5.1.2] | 1 |  |  | |  |
|  | *lipA* | Lipoic acid synthetase | 1 |  |  | |  |
|  |  | Lipoprotein releasing system, transmembrane protein, LolC/E family protein |  |  |  | |  |
|  | *lolA*  *lolD* | Outer membrane lipoprotein-sorting protein LolA  Lipoprotein releasing system ATP-binding protein LolD | 2 |  |  | |  |
|  |  |  |  |  |  |  |  |
|  | *lon1* | ATP-dependent protease La [EC:3.4.21.53] | 1 |  |  | |  |
|  | *lpdA1*  *lpdA2* | Dihydrolipoamide dehydrogenase [EC:1.8.1.4]  Dihydrolipoamide dehydrogenase [EC:1.8.1.4] | 2 |  |  | |  |
|  |  |  |  |  |  |  |  |
|  |  | LPS biosynthesis protein  LPS biosynthesis protein | 2 |  |  | |  |
|  |  |  |  |  |  |  |  |
|  | *lpxA*  *lpxB*  *lpxC*  *lpxD*  *lpxK* | Acyl-[acyl carrier protein]--UDP-N- acetylglucosamine O-acyltransferase [EC:2.3.1.129]  Lipid-A-disaccharide synthase [EC:2.4.1.182]  UDP-3-O-[3-hydroxymyristoyl] N-acetylglucosamine deacetylase [EC:3.5.1.-]  UDP-3-O-[3-hydroxymyristoyl] glucosamine N-acyltransferase [EC:2.3.1.-]  Tetraacyldisaccharide 4'-kinase [EC:2.7.1.130] | 5 |  |  | |  |
|  |  |  |  |  |  |  |  |
|  |  |  |  |  |  |  |  |
|  |  |  |  |  |  |  |  |
|  |  |  |  |  |  |  |  |
|  | *lspA* | Lipoprotein signal peptidase [EC:3.4.23.36] | 1 |  |  | |  |
|  | *lysC*  *lysE* | Aspartokinase [EC:2.7.2.4]  Lysine efflux permease | 2 |  |  | |  |
|  |  |  |  |  |  |  |  |
|  |  | Lysozyme  Lysozyme | 1 |  |  | | 1 |
|  |  |  |  |  |  |  |  |
|  | *lysS* | Lysyl-tRNA synthetase [EC:6.1.1.6] | 1 |  |  | |  |
|  | *maf* | Nucleotide-binding protein implicated in inhibition of septum formation | 1 |  |  | |  |
|  | *manC*  *manC*  *manC* | Mannose-1-phosphate guanylyltransferase (SPLIT GENE) |  | 1 | 3 | |  |
|  |  | Mannose-1-phosphate guanylyltransferase (SPLIT GENE) |  |  |  |  |  |
|  |  | Mannose-1-phosphate guanylyltransferase (SPLIT GENE) |  |  |  |  |  |
|  | *map* | Methionine aminopeptidase type I [EC:3.4.11.18] | 1 |  |  | |  |
|  | *mazG* | MazG-like protein | 1 |  |  | |  |
|  | *mccF2*  *mccF* | Microcin C7 resistance protein  Microcin C7 self-immunity protein | 2 |  |  | |  |
|  |  |  |  |  |  |  |  |
|  | *mdh* | Malate dehydrogenase [EC:1.1.1.37] | 1 |  |  | |  |
|  | *mdlB*  *mdlB* | ABC-type multidrug transport system, ATPase and permease components (SPLIT GENE)  ABC-type multidrug transport system, ATPase and permease components (SPLIT GENE) |  | 1 | 2 | |  |
|  |  |  |  |  |  |  |  |
|  |  | Membrane protein implicated in regulation of membrane protease activity | 1 |  |  | |  |
|  | *metG* | Methionyl-tRNA synthetase [EC:6.1.1.10] | 1 |  |  | |  |
|  |  | Methylated-DNA--protein-cysteine methyltransferase [EC:2.1.1.63] (SPLIT GENE)  Methylated-DNA--protein-cysteine methyltransferase [EC:2.1.1.63] (SPLIT GENE) |  | 1 | 2 | |  |
|  |  |  |  |  |  |  |  |
|  | *metK*  *metK*  *metK* | S-adenosylmethionine synthetase (SPLIT GENE)  S-adenosylmethionine synthetase (SPLIT GENE)  S-adenosylmethionine synthetase (SPLIT GENE) |  | 1 | 3 | |  |
|  |  |  |  |  |  |  |  |
|  |  |  |  |  |  |  |  |
|  | *mfd* | Transcription-repair coupling factor | 1 |  |  | |  |
|  | *mfp*  *mfp* | Membrane-fusion protein component of the RND family transporter (SPLIT GENE)  Membrane-fusion protein component of the RND family transporter (SPLIT GENE) |  | 1 | 2 | |  |
|  |  |  |  |  |  |  |  |
|  |  | MFS-type sugar transporter (FRAGMENT)  MFS-type sugar transporter (FRAGMENT) |  |  |  | | 2 |
|  |  |  |  |  |  |  |  |
|  |  | Mg chelatase-related protein (SPLIT GENE)  Mg chelatase-related protein (SPLIT GENE)  Mg chelatase-related protein (SPLIT GENE) |  | 1 | 3 | |  |
|  |  |  |  |  |  |  |  |
|  |  |  |  |  |  |  |  |
|  | *mgtE* | Magnesium transporter | 1 |  |  | |  |
|  | *miaA* | tRNA delta(2)-isopentenylpyrophosphate transferase [EC:2.5.1.8] | 1 |  |  | |  |
|  | *MiaB*  *miaB* | MiaB-like tRNA modifying enzyme  tRNA-i(6)A37 thiotransferase enzyme MiaB | 2 |  |  | |  |
|  |  |  |  |  |  |  |  |
|  | *mkl* | Ribonucleotide ABC transporter ATP-binding protein | 1 |  |  | |  |
|  | *mltE1*  *mltE2* | Soluble lytic murein transglycosylase precursor  Soluble lytic murein transglycosylase and related regulatory proteins | 2 |  |  | |  |
|  |  |  |  |  |  |  |  |
|  | *mnhB*  *mnhC*  *mnhE* | Multisubunit Na+/H+ antiporter, MnhB subunit  Multisubunit Na+/H+ antiporter, MnhC subunit  Multisubunit Na+/H+ antiporter, MnhE subunit  Monovalent cation/proton antiporter, MnhG/PhaG subunit  Multisubunit Na+/H+ antiporter, MnhF subunit | 5 |  |  | |  |
|  |  |  |  |  |  |  |  |
|  |  |  |  |  |  |  |  |
|  |  |  |  |  |  |  |  |
|  |  |  |  |  |  |  |  |
|  | *mpg* | DNA-3-methyladenine glycosidase [EC:3.2.2.21] | 1 |  |  | |  |
|  | *mpp* | Mitochondrial protease [EC:3.4.-.-] | 1 |  |  | |  |
|  | *mraW*  *mraY1*  *mraY2*  *mraZ* | S-adenosyl-methyltransferase MraW [EC:2.1.1.-]  Phospho-N-acetylmuramoyl-pentapeptide-transferase [EC:2.7.8.13]  Undecaprenyl-phosphate alpha-N-acetylglucosaminyltransferase [EC:2.4.1.-]  MraZ protein | 4 |  |  | |  |
|  |  |  |  |  |  |  |  |
|  |  |  |  |  |  |  |  |
|  |  |  |  |  |  |  |  |
|  | *mrcA* | Penicillin-binding protein 1A | 1 |  |  | |  |
|  | *mreB*  *mreC* | Rod shape-determining protein MreB  Rod shape-determining protein MreC | 2 |  |  | |  |
|  |  |  |  |  |  |  |  |
|  | *mrp* | Mrp |  |  |  | |  |
|  | *msbA1*  *msbA2* | Multidrug resistance protein  Multidrug resistance protein  Multidrug resistance transporter protein (SPLIT GENE)  Multidrug resistance transporter protein (SPLIT GENE) | 2 | 1 | 2 | |  |
|  |  |  |  |  |  |  |  |
|  |  |  |  |  |  |  |  |
|  |  |  |  |  |  |  |  |
|  | *murA*  *murB*  *murC*  *murD*  *murE*  *murF*  *murG* | UDP-N-acetylglucosamine 1-carboxyvinyltransferase [EC:2.5.1.7]  UDP-N-acetylenolpyruvoylglucosamine reductase [EC:1.1.1.158]  UDP-N-acetylmuramate--alanine ligase [EC:6.3.2.8]  UDP-N-acetylmuramoylalanine--D-glutamate ligase [EC:6.3.2.9]  UDP-N-acetylmuramoylalanyl-D-glutamate-- 2,6-diaminopimelate ligase [EC:6.3.2.13]  UDP-N-acetylmuramoylalanyl-D-glutamyl- 2,6-diaminopimelate--D-alanyl-D-alanyl ligase [EC:6.3.2  UDP-N-acetylglucosamine--N-acetylmuramyl-(pentapeptide) pyrophosphoryl-undecaprenol N-acetylgl | 7 |  |  | |  |
|  |  |  |  |  |  |  |  |
|  |  |  |  |  |  |  |  |
|  |  |  |  |  |  |  |  |
|  |  |  |  |  |  |  |  |
|  |  |  |  |  |  |  |  |
|  |  |  |  |  |  |  |  |
|  | *mutL*  *mutM*  *mutS*  *mutT* | DNA mismatch repair protein MutL  Formamidopyrimidine-DNA glycosidase [EC:3.2.2.23]  DNA mismatch repair protein MutS  ADP-ribose pyrophosphatase MutT [EC:3.6.1.-] | 4 |  |  | |  |
|  |  |  |  |  |  |  |  |
|  |  |  |  |  |  |  |  |
|  |  |  |  |  |  |  |  |
|  | *mviN* | Integral membrane protein MviN | 1 |  |  | |  |
|  |  | N6-adenine-specific methylase | 1 |  |  | |  |
|  |  | NACHT family NTPase (FRAGMENT)  NACHT family NTPase (SPLIT GENE)  NACHT family NTPase (SPLIT GENE) |  | 1 | 2 | | 1 |
|  |  |  |  |  |  |  |  |
|  |  |  |  |  |  |  |  |
|  |  | NAD-specific glutamate dehydrogenase | 1 |  |  | |  |
|  |  | NADH:ubiquinone oxidoreductase 17.2 kD subunit | 1 |  |  | |  |
|  | *ndk* | Nucleoside diphosphate kinase [EC:2.7.4.6] | 1 |  |  | |  |
|  | *nhaA* | Na+/H+ antiporter NhaA | 1 |  |  | |  |
|  |  | NifU-like protein | 1 |  |  | |  |
|  | *nlpD1*  *nlpD2* | Membrane-bound metallopeptidase  Membrane-bound metallopeptidase | 2 |  |  | |  |
|  |  |  |  |  |  |  |  |
|  | *nrdA*  *nrdB*  *nrdG* | Ribonucleoside-diphosphate reductase alpha chain [EC:1.17.4.1]  Ribonucleoside-diphosphate reductase beta chain [EC:1.17.4.1]  Organic radical activating enzymes | 3 |  |  | |  |
|  |  |  |  |  |  |  |  |
|  |  |  |  |  |  |  |  |
|  |  | NT (nucleotidyltransferase) domain and HEPN (higher eukarytoes and prokaryotes nucleotide-binding) d  NT (nucleotidyltransferase) domain and HEPN (higher eukarytoes and prokaryotes nucleotide-binding) d  NT (nucleotidyltransferase) domain and HEPN (higher eukarytoes and prokaryotes nucleotide-binding) d  NT (nucleotidyltransferase) domain and HEPN (higher eukarytoes and prokaryotes nucleotide-binding) d | 4 |  |  | |  |
|  |  |  |  |  |  |  |  |
|  |  |  |  |  |  |  |  |
|  |  |  |  |  |  |  |  |
|  | *nth* | Endonuclease III [EC:4.2.99.18] | 1 |  |  | |  |
|  | *ntrX*  *ntrY* | Nitrogen assimilation regulatory protein NtrX  Nitrogen regulation protein NtrY | 2 |  |  | |  |
|  |  |  |  |  |  |  |  |
|  |  | Nucleotidyltransferase substrate binding protein | 1 |  |  | |  |
|  | *nudH* | (Di)nucleoside polyphosphate hydrolase [EC:3.6.1.-] | 1 |  |  | |  |
|  | *nuoA*  *nuoB*  *nuoC*  *nuoD*  *nuoE*  *nuoF*  *nuoG*  *nuoH*  *nuoI*  *nuoJ*  *nuoK*  *nuoL1*  *nuoL2*  *nuoL3*  *nuoM*  *nuoN1*  *nuoN2* | NADH dehydrogenase I chain A [EC:1.6.5.3]  NADH dehydrogenase I chain B [EC:1.6.5.3]  NADH dehydrogenase I chain C [EC:1.6.5.3]  NADH dehydrogenase I chain D [EC:1.6.5.3]  NADH dehydrogenase I chain E [EC:1.6.5.3]  NADH dehydrogenase I chain F [EC:1.6.5.3]  NADH dehydrogenase I chain G [EC:1.6.5.3]  NADH dehydrogenase I chain H [EC:1.6.5.3]  NADH dehydrogenase I chain I [EC:1.6.5.3]  NADH dehydrogenase I chain J [EC:1.6.5.3]  NADH dehydrogenase I chain K [EC:1.6.5.3]  NADH dehydrogenase I chain L [EC:1.6.5.3]  NADH dehydrogenase I chain L [EC:1.6.5.3]  NADH dehydrogenase I chain N [EC:1.6.5.3]  NADH dehydrogenase I chain M [EC:1.6.5.3]  NADH:ubiquinone oxidoreductase subunit 2 (chain N) [EC:1.6.5.3]  NADH:ubiquinone oxidoreductase subunit 2 (chain N) [EC:1.6.5.3] | 17 |  |  | |  |
|  |  |  |  |  |  |  |  |
|  |  |  |  |  |  |  |  |
|  |  |  |  |  |  |  |  |
|  |  |  |  |  |  |  |  |
|  |  |  |  |  |  |  |  |
|  |  |  |  |  |  |  |  |
|  |  |  |  |  |  |  |  |
|  |  |  |  |  |  |  |  |
|  |  |  |  |  |  |  |  |
|  |  |  |  |  |  |  |  |
|  |  |  |  |  |  |  |  |
|  |  |  |  |  |  |  |  |
|  |  |  |  |  |  |  |  |
|  |  |  |  |  |  |  |  |
|  |  |  |  |  |  |  |  |
|  |  |  |  |  |  |  |  |
|  | *nusA*  *nusB*  *nusG* | N utilization substance protein A, transcription termination factor NusA  N utilization substance protein B  Transcription antitermination protein NusG | 3 |  |  | |  |
|  |  |  |  |  |  |  |  |
|  |  |  |  |  |  |  |  |
|  | *obg* | GTP-binding protein | 1 |  |  | |  |
|  |  | Oligoketide cyclase/lipid transport protein | 1 |  |  | |  |
|  | *omp1*  *omp*  *ompA ; sca0*  *ompB ; sca5*  *ompR*  *ompW* | Outer membrane protein omp1  17 kD surface antigen precursor  Outer memberane protein rOmpA  Outer membrane protein rOmpB  PetR  OmpW family outer-membrane protein | 6 |  |  | |  |
|  |  |  |  |  |  |  |  |
|  |  |  |  |  |  |  |  |
|  |  |  |  |  |  |  |  |
|  |  |  |  |  |  |  |  |
|  |  |  |  |  |  |  |  |
|  | *ostA* | Organic solvent tolerance protein-like protein | 1 |  |  | |  |
|  |  | Oxidoreductase (SPLIT GENE)  Oxidoreductase (SPLIT GENE) |  | 1 | 2 | |  |
|  |  |  |  |  |  |  |  |
|  | *paaJ*  *paaY* | Acetyl-CoA acetyltransferase [EC:2.3.1.9]  Carbonic anhydrases/acetyltransferases, isoleucine patch superfamily protein | 2 |  |  | |  |
|  |  |  |  |  |  |  |  |
|  | *pal* | Peptidoglycan-associated lipoprotein precursor |  |  |  | |  |
|  | *panF* | Sodium/pantothenate symporter | 1 |  |  | |  |
|  |  | ParB-like partition proteins | 1 |  |  | |  |
|  | *parC*  *parE* | Topoisomerase IV subunit A [EC:5.99.1.-]  DNA topoisomerase IV, B subunit [EC:5.99.1.-] | 2 |  |  | |  |
|  |  |  |  |  |  |  |  |
|  | *pat1* | Patatin-like phospholipase | 1 |  |  | |  |
|  | *pbpA1*  *pbpA2*  *pbpC*  *pbpE* | Penicillin-binding protein  Penicillin-binding protein  Bifunctional penicillin-binding protein 1C  Penicillin-binding protein 4* | 4 |  |  | |  |
|  |  |  |  |  |  |  |  |
|  |  |  |  |  |  |  |  |
|  |  |  |  |  |  |  |  |
|  | *pccB* | Propionyl-CoA carboxylase beta chain precursor [EC:6.4.1.3] | 1 |  |  | |  |
|  | *pcnB* | Poly(A) polymerase [EC:2.7.7.19] | 1 |  |  | |  |
|  | *pdhA*  *pdhB*  *pdhC* | Pyruvate dehydrogenase e1 component, alpha subunit precursor [EC:1.2.4.1]  Pyruvate dehydrogenase E1 component, beta subunit precursor [EC:1.2.4.1]  Pyruvate dehydrogenase complex dihydrolipoamide acetyltransferase [EC:2.3.1.12] | 3 |  |  | |  |
|  |  |  |  |  |  |  |  |
|  |  |  |  |  |  |  |  |
|  |  | PemK-like growth inhibitor | 1 |  |  | |  |
|  | *pepA*  *pepP* | Aminopeptidase A [EC:3.4.11.1]  Aminopeptidase P [EC:3.4.11.9] | 2 |  |  | |  |
|  |  |  |  |  |  |  |  |
|  |  | Periplasmic protein  Periplasmic protein TonB, links inner and outer membranes | 2 |  |  | |  |
|  |  |  |  |  |  |  |  |
|  | *perM* | Permease PerM-like protein | 1 |  |  | |  |
|  | *petA*  *petB* | Ubiquinol-cytochrome c reductase, iron-sulfur subunit [EC:1.10.2.2]  Cytochrome b | 2 |  |  | |  |
|  |  |  |  |  |  |  |  |
|  | *pgpA* | Phosphatidylglycerophosphatase A [EC:3.1.3.27] | 1 |  |  | |  |
|  | *pgsA* | CDP-diacylglycerol--glycerol-3-phosphate 3-phosphatidyltransferase [EC:2.7.8.5] | 1 |  |  | |  |
|  | *phaC*  *phaC* | Poly(3-hydroxyalkanoate) synthetase (SPLIT GENE)  Poly(3-hydroxyalkanoate) synthetase (SPLIT GENE) |  | 1 | 2 | |  |
|  |  |  |  |  |  |  |  |
|  |  | Phage portal protein (FRAGMENT) |  |  |  | | 1 |
|  |  | Phasin family protein | 1 |  |  | |  |
|  | *phbB*  *phbC*  *phbC*  *phbC* | Acetoacetyl-CoA reductase [EC:1.1.1.100]  Poly-beta-hydroxybutyrate polymerase (SPLIT GENE)  Poly-beta-hydroxybutyrate polymerase (SPLIT GENE)  Poly-beta-hydroxybutyrate polymerase (SPLIT GENE) | 1 | 1 | 3 | |  |
|  |  |  |  |  |  |  |  |
|  |  |  |  |  |  |  |  |
|  |  |  |  |  |  |  |  |
|  | *pheS*  *pheT* | Phenylalanyl-tRNA synthetase alpha chain [EC:6.1.1.20]  Phenylalanyl-tRNA synthetase beta chain [EC:6.1.1.20] | 2 |  |  | |  |
|  |  |  |  |  |  |  |  |
|  | *phnP* | Metal-dependent hydrolases of the beta-lactamase superfamily I | 1 |  |  | |  |
|  |  | Phosphatidylethanolamine-binding protein PEBP | 1 |  |  | |  |
|  | *pkcI* | Protein kinase C inhibitor 1 | 1 |  |  | |  |
|  |  | Plasmid maintenance system antidote protein  Plasmid maintenance system antidote protein | 2 |  |  | |  |
|  |  |  |  |  |  |  |  |
|  | *pldA* | Phospholipase D superfamily protein PLD | 1 |  |  | |  |
|  | *plsC* | 1-acyl-sn-glycerol-3-phosphate acyltransferase [EC:2.3.1.51] | 1 |  |  | |  |
|  | *pnp* | Polyribonucleotide nucleotidyltransferase [EC:2.7.7.8] | 1 |  |  | |  |
|  | *pntA1*  *pntA2*  *pntB* | NAD(P) transhydrogenase subunit alpha [EC:1.6.1.1]  NAD(P) transhydrogenase subunit alpha [EC:1.6.1.1]  NAD(p) transhydrogenase subunit beta [EC:1.6.1.1] | 3 |  |  | |  |
|  |  |  |  |  |  |  |  |
|  |  |  |  |  |  |  |  |
|  | *polA* | DNA polymerase I [EC:2.7.7.7] | 1 |  |  | |  |
|  |  | Poly-beta-hydroxyalkanoate depolymerase (SPLIT GENE)  Poly-beta-hydroxyalkanoate depolymerase (SPLIT GENE) |  | 1 | 2 | |  |
|  |  |  |  |  |  |  |  |
|  | *potE* | Putrescine-ornithine antiporter | 1 |  |  | |  |
|  | *ppa* | Inorganic pyrophosphatase [EC:3.6.1.1] | 1 |  |  | |  |
|  | *ppcE*  *ppcE*  *ppcE*  *ppcE*  *ppcE* | Prolyl endopeptidase precursor [EC:3.4.21.26] (SPLIT GENE)  Prolyl endopeptidase precursor [EC:3.4.21.26] (SPLIT GENE)  Prolyl endopeptidase precursor [EC:3.4.21.26] (SPLIT GENE)  Prolyl endopeptidase precursor [EC:3.4.21.26] (SPLIT GENE)  Prolyl endopeptidase precursor [EC:3.4.21.26] (SPLIT GENE) |  | 1 | 5 | |  |
|  |  |  |  |  |  |  |  |
|  |  |  |  |  |  |  |  |
|  |  |  |  |  |  |  |  |
|  |  |  |  |  |  |  |  |
|  | *ppdK* | Pyruvate,phosphate dikinase precursor [EC:2.7.9.1] | 1 |  |  | |  |
|  | *prfA*  *prfB* | Peptide chain release factor RF-1  Peptide chain release factor RF-2 | 2 |  |  | |  |
|  |  |  |  |  |  |  |  |
|  | *priA* | Primosomal protein N' | 1 |  |  | |  |
|  | *proP10*  *proP10*  *proP10*  *proP1*  *proP2*  *proP3*  *proP4*  *proP5*  *proP6*  *proP6*  *proP6*  *proP7*  *proP8*  *proP9*  *proP9_2*  *proP9_2* | Proline/betaine transporter (SPLIT GENE)  Proline/betaine transporter (SPLIT GENE)  Proline/betaine transporter (SPLIT GENE)  Proline/betaine transporter  Proline/betaine transporter  Proline/betaine transporter  Proline/betaine transporter  Proline/betaine transporter  Proline/betaine transporter (SPLIT GENE)  Proline/betaine transporter (SPLIT GENE)  Proline/betaine transporter (SPLIT GENE)  Proline/betaine transporter  Proline/betaine transporter (FRAGMENT)  Proline/betaine transporter  Proline/betaine transporter (SPLIT GENE)  Proline/betaine transporter (SPLIT GENE) | 7 | 3 | 8 | | 1 |
|  |  |  |  |  |  |  |  |
|  |  |  |  |  |  |  |  |
|  |  |  |  |  |  |  |  |
|  |  |  |  |  |  |  |  |
|  |  |  |  |  |  |  |  |
|  |  |  |  |  |  |  |  |
|  |  |  |  |  |  |  |  |
|  |  |  |  |  |  |  |  |
|  |  |  |  |  |  |  |  |
|  |  |  |  |  |  |  |  |
|  |  |  |  |  |  |  |  |
|  |  |  |  |  |  |  |  |
|  |  |  |  |  |  |  |  |
|  |  |  |  |  |  |  |  |
|  |  |  |  |  |  |  |  |
|  |  | Prophage antirepressor (SPLIT GENE)  Prophage antirepressor (SPLIT GENE) |  | 1 | 2 | |  |
|  |  |  |  |  |  |  |  |
|  | *proS* | Prolyl-tRNA synthetase [EC:6.1.1.15] | 1 |  |  | |  |
|  |  | Protocatechuate-3,4-dioxygenase, beta subunit [EC:1.13.11.3] | 1 |  |  | |  |
|  | *prs*  *prs* | Ribose-phosphate pyrophosphokinase [EC:2.7.6.1] (SPLIT GENE)  Ribose-phosphate pyrophosphokinase [EC:2.7.6.1] (SPLIT GENE) |  | 1 | 2 | |  |
|  |  |  |  |  |  |  |  |
|  | *prsA* | Protein export protein prsA precursor | 1 |  |  | |  |
|  | *psd* | Phosphatidylserine decarboxylase [EC:4.1.1.65] | 1 |  |  | |  |
|  | *pspE* | Rhodanese-related sulfurtransferase | 1 |  |  | |  |
|  | *pssA* | CDP-diacylglycerol--serine O-phosphatidyltransferase [EC:2.7.8.8] | 1 |  |  | |  |
|  | *pta*  *pta* | Phosphate acetyltransferase Pta [EC:2.3.1.8] (SPLIT GENE)  Phosphate acetyltransferase Pta [EC:2.3.1.8] (SPLIT GENE) |  | 1 | 2 | |  |
|  |  |  |  |  |  |  |  |
|  | *pth* | Peptidyl-tRNA hydrolase [EC:3.1.1.29] | 1 |  |  | |  |
|  | *ptrB* | Protease II [EC:3.4.21.83] | 1 |  |  | |  |
|  | *purC* | Phosphoribosylaminoimidazole-succinocarboxamide synthase [EC:6.3.2.6] | 1 |  |  | |  |
|  | *putP* | Na+/proline symporter and signal transduction histidine kinase | 1 |  |  | |  |
|  | *pyrG*  *pyrH* | CTP synthase [EC:6.3.4.2]  Uridylate kinase [EC:2.7.4.-] | 2 |  |  | |  |
|  |  |  |  |  |  |  |  |
|  | *queA* | S-adenosylmethionine:tRNA ribosyltransferase-isomerase [EC:5.-.-.-] | 1 |  |  | |  |
|  | *radA*  *radC*  *radC* | DNA repair protein RadA  DNA repair protein RadC (SPLIT GENE)  DNA repair protein RadC (SPLIT GENE) | 1 | 1 | 2 | |  |
|  |  |  |  |  |  |  |  |
|  |  |  |  |  |  |  |  |
|  | *rbfA* | Ribosome-binding factor A | 1 |  |  | |  |
|  |  | RDD family protein | 1 |  |  | |  |
|  | *recA*  *recB*  *recF*  *recG*  *recJ*  *recN*  *recO*  *recR* | RecA  family exonuclease  DNA replication and repair protein RecF  ATP-dependent DNA helicase RecG [EC:3.6.1.-]  Single-stranded-DNA-specific exonuclease RecJ [EC:3.1.-.-]  DNA repair protein RecN  DNA repair protein RecO  Recombination protein RecR | 8 |  |  | |  |
|  |  |  |  |  |  |  |  |
|  |  |  |  |  |  |  |  |
|  |  |  |  |  |  |  |  |
|  |  |  |  |  |  |  |  |
|  |  |  |  |  |  |  |  |
|  |  |  |  |  |  |  |  |
|  |  |  |  |  |  |  |  |
|  |  | Regulatory components of sensory transduction system | 1 |  |  | |  |
|  | *relB2* | DNA-damage-inducible protein J | 1 |  |  | |  |
|  |  | Response regulator (FRAGMENT) |  |  |  | | 1 |
|  | *rfaJ*  *rfaJ* | Lipopolysaccharide 1,2-glucosyltransferase RfaJ (SPLIT GENE)  Lipopolysaccharide 1,2-glucosyltransferase RfaJ (SPLIT GENE) |  | 1 | 2 | |  |
|  |  |  |  |  |  |  |  |
|  | *rfbA* | O-antigen export system permease protein RfbA | 2 |  |  | |  |
|  | *rfbE* | O-antigen export system ATP-binding protein RfbE |  |  |  | |  |
|  | *rffE* | UDP-N-acetylglucosamine 2-epimerase [EC:5.1.3.14] | 1 |  |  | |  |
|  | *rhlE* | ATP-dependent RNA helicase RhlE | 1 |  |  | |  |
|  | *rho* | Transcription termination factor | 1 |  |  | |  |
|  |  | Ribosomal large subunit pseudouridine synthases RluD subfamily protein [EC:4.2.1.70] |  |  |  | |  |
|  | *rickA* | Actin polymerization protein RickA | 1 |  |  | |  |
|  | *rimJ*  *rimM* | Ribosomal-protein-alanine acetyltransferase [EC:2.3.1.128]  16S rRNA processing protein RimM | 2 |  |  | |  |
|  |  |  |  |  |  |  |  |
|  | *rlpA* | Rare lipoprotein A precursor | 1 |  |  | |  |
|  | *rluA1*  *rluA2* | Ribosomal large subunit pseudouridine synthase [EC:4.2.1.70]  Ribosomal large subunit pseudouridine synthase [EC:4.2.1.70] | 2 |  |  | |  |
|  |  |  |  |  |  |  |  |
|  |  | RmuC family protein | 1 |  |  | |  |
|  | *rnc* | Ribonuclease III [EC:3.1.26.3] | 1 |  |  | |  |
|  |  | RND efflux system, outer membrane protein (SPLIT GENE)  RND efflux system, outer membrane protein (SPLIT GENE)  RND family efflux transporter (SPLIT GENE)  RND family efflux transporter (SPLIT GENE)  RND family efflux transporter (SPLIT GENE)  RND family efflux transporter (SPLIT GENE)  RND family efflux transporter (SPLIT GENE)  RND family efflux transporter (SPLIT GENE) |  | 2 | 8 | |  |
|  |  |  |  |  |  |  |  |
|  |  |  |  |  |  |  |  |
|  |  |  |  |  |  |  |  |
|  |  |  |  |  |  |  |  |
|  |  |  |  |  |  |  |  |
|  |  |  |  |  |  |  |  |
|  |  |  |  |  |  |  |  |
|  | *rnd2*  *rnd*  *rne*  *rnhA*  *rnhB*  *rnpA* | Ribonuclease D [EC:3.1.26.3]  Ribonuclease D [EC:3.1.26.3]  Ribonuclease E [EC:3.1.4.-]  Ribonuclease H [EC:3.1.26.4]  Ribonuclease HII [EC:3.1.26.4]  Ribonuclease P [EC:3.1.26.5] | 6 |  |  | |  |
|  |  |  |  |  |  |  |  |
|  |  |  |  |  |  |  |  |
|  |  |  |  |  |  |  |  |
|  |  |  |  |  |  |  |  |
|  |  |  |  |  |  |  |  |
|  | *rodA* | Rod shape-determining protein rodA | 1 |  |  | |  |
|  | *rph* | Ribonuclease PH [EC:2.7.7.56] | 1 |  |  | |  |
|  | *rplA*  *rplB*  *rplC*  *rplD*  *rplE*  *rplF*  *rplI*  *rplJ*  *rplK*  *rplL*  *rplM*  *rplN*  *rplO*  *rplP*  *rplQ*  *rplR*  *rplS*  *rplT*  *rplU*  *rplV*  *rplW*  *rplX*  *rplY* | 50S ribosomal protein L1  50S ribosomal protein L2  50S ribosomal protein L3  50S ribosomal protein L4  50S ribosomal protein L5  50S ribosomal protein L6  50S ribosomal protein L9  50S ribosomal protein L10  50S ribosomal protein L11  50S ribosomal protein L7/L12  50S ribosomal protein L13  50S ribosomal protein L14  50S ribosomal protein L15  50S ribosomal protein L16  50S ribosomal protein L17  50S ribosomal protein L18  50S ribosomal protein L19  50S ribosomal protein L20  50S ribosomal protein L21  50S ribosomal protein L22  50S ribosomal protein L23  50S ribosomal protein L24  50S ribosomal protein L25 | 23 |  |  | |  |
|  |  |  |  |  |  |  |  |
|  |  |  |  |  |  |  |  |
|  |  |  |  |  |  |  |  |
|  |  |  |  |  |  |  |  |
|  |  |  |  |  |  |  |  |
|  |  |  |  |  |  |  |  |
|  |  |  |  |  |  |  |  |
|  |  |  |  |  |  |  |  |
|  |  |  |  |  |  |  |  |
|  |  |  |  |  |  |  |  |
|  |  |  |  |  |  |  |  |
|  |  |  |  |  |  |  |  |
|  |  |  |  |  |  |  |  |
|  |  |  |  |  |  |  |  |
|  |  |  |  |  |  |  |  |
|  |  |  |  |  |  |  |  |
|  |  |  |  |  |  |  |  |
|  |  |  |  |  |  |  |  |
|  |  |  |  |  |  |  |  |
|  |  |  |  |  |  |  |  |
|  |  |  |  |  |  |  |  |
|  |  |  |  |  |  |  |  |
|  | *rpmA*  *rpmB*  *rpmC*  *rpmD*  *rpmE*  *rpmF*  *rpmG*  *rpmH*  *rpmI*  *rpmJ* | 50S ribosomal protein L27  50S ribosomal protein L28  50S ribosomal protein L29  50S ribosomal protein L30  50S ribosomal protein L31  50S ribosomal protein L32  50S ribosomal protein L33  50S ribosomal protein L34  50S ribosomal protein L35  50S ribosomal protein L36 | 10 |  |  | |  |
|  |  |  |  |  |  |  |  |
|  |  |  |  |  |  |  |  |
|  |  |  |  |  |  |  |  |
|  |  |  |  |  |  |  |  |
|  |  |  |  |  |  |  |  |
|  |  |  |  |  |  |  |  |
|  |  |  |  |  |  |  |  |
|  |  |  |  |  |  |  |  |
|  |  |  |  |  |  |  |  |
|  | *rpoA*  *rpoB*  *rpoC*  *rpoD*  *rpoH*  *rpoZ* | DNA-directed RNA polymerase alpha chain [EC:2.7.7.6]  DNA-directed RNA polymerase beta chain [EC:2.7.7.6]  DNA-directed RNA polymerase beta prime chain [EC:2.7.7.6]  RNA polymerase sigma factor  RNA polymerase sigma-32 factor  DNA-directed RNA polymerase omega chain [EC:2.7.7.6] | 6 |  |  | |  |
|  |  |  |  |  |  |  |  |
|  |  |  |  |  |  |  |  |
|  |  |  |  |  |  |  |  |
|  |  |  |  |  |  |  |  |
|  |  |  |  |  |  |  |  |
|  | *rpsA*  *rpsB*  *rpsC*  *rpsD*  *rpsE*  *rpsF*  *rpsG*  *rpsH*  *rpsI*  *rpsJ*  *rpsK*  *rpsL*  *rpsM*  *rpsN*  *rpsO*  *rpsP*  *rpsQ*  *rpsR*  *rpsS*  *rpsT*  *rpsU* | 30S ribosomal protein S1  30S ribosomal protein S2  30S ribosomal protein S3  30S ribosomal protein S4  30S ribosomal protein S5  30S ribosomal protein S6  30S ribosomal protein S7  30S ribosomal protein S8  30S ribosomal protein S9  30S ribosomal protein S10  30S ribosomal protein S11  30S ribosomal protein S12  30S ribosomal protein S13  30S ribosomal protein S14  30S ribosomal protein S15  30S ribosomal protein S16  30S ribosomal protein S17  30S ribosomal protein S18  30S ribosomal protein S19  30S ribosomal protein S20  30S ribosomal protein S21 | 21 |  |  | |  |
|  |  |  |  |  |  |  |  |
|  |  |  |  |  |  |  |  |
|  |  |  |  |  |  |  |  |
|  |  |  |  |  |  |  |  |
|  |  |  |  |  |  |  |  |
|  |  |  |  |  |  |  |  |
|  |  |  |  |  |  |  |  |
|  |  |  |  |  |  |  |  |
|  |  |  |  |  |  |  |  |
|  |  |  |  |  |  |  |  |
|  |  |  |  |  |  |  |  |
|  |  |  |  |  |  |  |  |
|  |  |  |  |  |  |  |  |
|  |  |  |  |  |  |  |  |
|  |  |  |  |  |  |  |  |
|  |  |  |  |  |  |  |  |
|  |  |  |  |  |  |  |  |
|  |  |  |  |  |  |  |  |
|  |  |  |  |  |  |  |  |
|  |  |  |  |  |  |  |  |
|  | *rrf* | Ribosome recycling factor | 1 |  |  | |  |
|  | *rrmJ* | Ribosomal RNA large subunit methyltransferase J | 1 |  |  | |  |
|  | *ruvA*  *ruvB*  *ruvC* | Holliday junction DNA helicase RuvA  Holliday junction DNA helicase RuvB  Crossover junction endodeoxyribonuclease RuvC [EC:3.1.22.4] | 3 |  |  | |  |
|  |  |  |  |  |  |  |  |
|  |  |  |  |  |  |  |  |
|  | *sam* | S-adenosylmethionine transporter | 1 |  |  | |  |
|  | *sca10*  *sca10*  *sca13*  *sca13*  *sca13*  *sca13*  *sca13*  *sca13*  *sca13*  *sca13*  *sca13*  *sca13*  *sca1*  *sca2*  *sca3*  *sca4*  *sca8*  *sca8*  *sca8*  *sca9* | Cell surface antigen Sca10 (SPLIT GENE)  Cell surface antigen Sca10 (SPLIT GENE)  Cell surface antigen Sca13 (SPLIT GENE)  Cell surface antigen Sca13 (SPLIT GENE)  Cell surface antigen Sca13 (SPLIT GENE)  Cell surface antigen Sca13 (SPLIT GENE)  Cell surface antigen Sca13 (SPLIT GENE)  Cell surface antigen Sca13 (SPLIT GENE)  Cell surface antigen Sca13 (SPLIT GENE)  Cell surface antigen Sca13 (SPLIT GENE)  Cell surface antigen Sca13 (SPLIT GENE)  Cell surface antigen Sca13 (SPLIT GENE)  Cell surface antigen Sca1  Cell surface antigen Sca2  Cell surface antigen Sca3 (FRAGMENT)  Cell surface antigen Sca4  Cell surface antigen Sca8 (SPLIT GENE)  Cell surface antigen Sca8 (SPLIT GENE)  Cell surface antigen Sca8 (SPLIT GENE)  Cell surface antigen Sca9 (FRAGMENT) | 3 | 3 | 15 | | 2 |
|  |  |  |  |  |  |  |  |
|  |  |  |  |  |  |  |  |
|  |  |  |  |  |  |  |  |
|  |  |  |  |  |  |  |  |
|  |  |  |  |  |  |  |  |
|  |  |  |  |  |  |  |  |
|  |  |  |  |  |  |  |  |
|  |  |  |  |  |  |  |  |
|  |  |  |  |  |  |  |  |
|  |  |  |  |  |  |  |  |
|  |  |  |  |  |  |  |  |
|  |  |  |  |  |  |  |  |
|  |  |  |  |  |  |  |  |
|  |  |  |  |  |  |  |  |
|  |  |  |  |  |  |  |  |
|  |  |  |  |  |  |  |  |
|  |  |  |  |  |  |  |  |
|  |  |  |  |  |  |  |  |
|  |  |  |  |  |  |  |  |
|  |  | Sco2 protein precursor  Sco2 protein precursor | 2 |  |  | |  |
|  |  |  |  |  |  |  |  |
|  | *scoA*  *scoB* | Succinyl-CoA:3-ketoacid-coenzyme A transferase  Succinyl-CoA:3-ketoacid-coenzyme A transferase subunit B [EC:2.8.3.5] | 2 |  |  | |  |
|  |  |  |  |  |  |  |  |
|  | *sdhA*  *sdhB*  *sdhC*  *sdhD* | Succinate dehydrogenase flavoprotein subunit [EC:1.3.99.1]  Succinate dehydrogenase iron-sulfur protein [EC:1.3.99.1]  Succinate dehydrogenase cytochrome b-556 subunit [EC:1.3.99.1]  Succinate dehydrogenase hydrophobic membrane anchor protein [EC:1.3.99.1] | 4 |  |  | |  |
|  |  |  |  |  |  |  |  |
|  |  |  |  |  |  |  |  |
|  |  |  |  |  |  |  |  |
|  | *sec59* | Dolichol kinase | 1 |  |  | |  |
|  | *secA*  *secB*  *secD*  *secE*  *secF*  *secG*  *secY* | Preprotein translocase secA subunit  Protein-export protein secB  Protein-export membrane protein secD  Preprotein translocase SecE subunit  Protein-export membrane protein secF  Protein-export membrane protein secG  Preprotein translocase secY subunit | 7 |  |  | |  |
|  |  |  |  |  |  |  |  |
|  |  |  |  |  |  |  |  |
|  |  |  |  |  |  |  |  |
|  |  |  |  |  |  |  |  |
|  |  |  |  |  |  |  |  |
|  |  |  |  |  |  |  |  |
|  |  | Septum formation initiator | 1 |  |  | |  |
|  |  | Serine esterase | 1 |  |  | |  |
|  | *serS* | Seryl-tRNA synthetase [EC:6.1.1.11] | 1 |  |  | |  |
|  |  | Signal transduction histidine kinase (SPLIT GENE)  Signal transduction histidine kinase (SPLIT GENE) |  | 1 | 2 | |  |
|  |  |  |  |  |  |  |  |
|  |  | Site-specific DNA methylase | 1 |  |  | |  |
|  | *smpA*  *smpB* | tmRNA-binding protein  tmRNA-binding protein | 2 |  |  | |  |
|  |  |  |  |  |  |  |  |
|  | *sodB* | Superoxide dismutase [EC:1.15.1.1] | 1 |  |  | |  |
|  | *soj* | ATPase involved in chromosome partitioning | 1 |  |  | |  |
|  | *spl1* | NifS-like protein | 1 |  |  | |  |
|  | *spoT11, spoTd*  *spoT13*  *spoT13*  *spoT15*  *spoT1*  *spoT2, spoTa*  *spoT3*  *spoT4*  *spoT4*  *spoT6*  *spoT6* | Guanosine polyphosphate pyrophosphohydrolase/synthetase  Guanosine polyphosphate pyrophosphohydrolase/synthetase containing ankyrin repeat (SPLIT GEN  Guanosine polyphosphate pyrophosphohydrolase/synthetase containing ankyrin repeat (SPLIT GEN  Guanosine polyphosphate pyrophosphohydrolase/synthetase  Guanosine polyphosphate pyrophosphohydrolase/synthetase (FRAGMENT)  Guanosine polyphosphate pyrophosphohydrolase/synthetase  Guanosine polyphosphate pyrophosphohydrolase/synthetase (FRAGMENT)  Guanosine polyphosphate pyrophosphohydrolase/synthetase (SPLIT GENE)  Guanosine polyphosphate pyrophosphohydrolase/synthetase (SPLIT GENE)  Guanosine polyphosphate pyrophosphohydrolase/synthetase (SPLIT GENE)  Guanosine polyphosphate pyrophosphohydrolase/synthetase (SPLIT GENE) | 5 | 3 | 6 | |  |
|  |  |  |  |  |  |  |  |
|  |  |  |  |  |  |  |  |
|  |  |  |  |  |  |  |  |
|  |  |  |  |  |  |  |  |
|  |  |  |  |  |  |  |  |
|  |  |  |  |  |  |  |  |
|  |  |  |  |  |  |  |  |
|  |  |  |  |  |  |  |  |
|  |  |  |  |  |  |  |  |
|  |  |  |  |  |  |  |  |
|  | *sppA1*  *sppA2* | Signal peptide peptidase SppA, 36K type  Signal peptide peptidase SppA, 36K type [EC:3.4.-.-] | 2 |  |  | |  |
|  |  |  |  |  |  |  |  |
|  | *ssb* | Single-stranded DNA-binding protein | 1 |  |  | |  |
|  |  | Strees induced DNA-binding Dps family protein | 1 |  |  | |  |
|  | *sucA*  *sucB*  *sucC*  *sucD* | 2-oxoglutarate dehydrogenase E1 component [EC:1.2.4.2]  Dihydrolipoamide acetyltransferase component [EC:2.3.1.61]  Succinyl-CoA synthetase beta chain [EC:6.2.1.5]  Succinyl-CoA synthetase alpha chain [EC:6.2.1.5] | 4 |  |  | |  |
|  |  |  |  |  |  |  |  |
|  |  |  |  |  |  |  |  |
|  |  |  |  |  |  |  |  |
|  | *suhB* | Extragenic suppressor protein suhB | 1 |  |  | |  |
|  |  | Superfamily I DNA and RNA helicases (SPLIT GENE)  Superfamily I DNA and RNA helicases (SPLIT GENE)  Superfamily I DNA and RNA helicases (SPLIT GENE)  Superfamily I DNA and RNA helicases (SPLIT GENE)  Superfamily I DNA and RNA helicases (SPLIT GENE)  Superfamily I DNA and RNA helicases (SPLIT GENE) |  | 1 | 6 | |  |
|  |  |  |  |  |  |  |  |
|  |  |  |  |  |  |  |  |
|  |  |  |  |  |  |  |  |
|  |  |  |  |  |  |  |  |
|  |  |  |  |  |  |  |  |
|  | *surA* | Parvulin-like peptidyl-prolyl isomerase | 1 |  |  | |  |
|  | *surf1* | Surfeit locus protein 1 | 1 |  |  | |  |
|  | *tagD* | Glycerol-3-phosphate cytidyltransferase TagD | 1 |  |  | |  |
|  | *tatA*  *tatC* | Twin-arginine translocation protein TatA  Sec-independent protein translocase protein TatC | 2 |  |  | |  |
|  |  |  |  |  |  |  |  |
|  | *tdcB* | Threonine dehydratase [EC:4.2.1.16] | 1 |  |  | |  |
|  | *tdpX1* | Thioredoxin peroxidase 1 [EC:1.6.4.-] | 1 |  |  | |  |
|  |  | Tellurite resistance protein-related protein (FRAGMENT) |  |  |  | | 1 |
|  |  | terC; Tellurium resistance protein TerC |  |  |  | |  |
|  |  | Tetratricopeptide repeat-containing protein | 1 |  |  | |  |
|  |  | Tetratricopeptide repeat-containing protein (FRAGMENT) |  |  |  | | 1 |
|  |  | Tetratricopeptide repeat-containing protein (SPLIT GENE)  Tetratricopeptide repeat-containing protein (SPLIT GENE) |  | 1 | 2 | |  |
|  |  |  |  |  |  |  |  |
|  | *tfoX* | Regulator of competence-specific genes TfoX | 1 |  |  | |  |
|  | *tgt* | Queuine tRNA-ribosyltransferase [EC:2.4.2.29] | 1 |  |  | |  |
|  |  | Thermostable carboxypeptidase | 1 |  |  | |  |
|  | *thrS* | Threonyl-tRNA synthetase [EC:6.1.1.3] | 1 |  |  | |  |
|  | *thyX* | Thymidylate synthase, flavin-dependent [EC:2.1.1.148] | 1 |  |  | |  |
|  | *tig* | Trigger factor | 1 |  |  | |  |
|  |  | tilS, mesJ; tRNA(Ile)-lysidine synthetase |  |  |  | |  |
|  | *tlc1*  *tlc2*  *tlc3*  *tlc4*  *tlc5* | ATP/ADP translocase  ATP/ADP translocase  ATP/ADP translocase  ATP/ADP translocase  ATP/ADP translocase | 5 |  |  | |  |
|  |  |  |  |  |  |  |  |
|  |  |  |  |  |  |  |  |
|  |  |  |  |  |  |  |  |
|  |  |  |  |  |  |  |  |
|  | *tlpA* | Thiol:disulfide interchange protein tlpA | 1 |  |  | |  |
|  | *tlyA*  *tlyC* | Hemolysin A  Hemolysin C | 2 |  |  | |  |
|  |  |  |  |  |  |  |  |
|  | *tme* | Malate oxidoreductase [EC:1.1.1.38] and phosphate acetyltransferase [EC:2.3.1.8] | 1 |  |  | |  |
|  | *tmk1* | Thymidylate kinase [EC:2.7.4.9] | 1 |  |  | |  |
|  | *tolB*  *tolC*  *tolQ*  *tolR* | TolB protein precursor  Type I secretion outer membrane protein TolC  TolQ  TolR | 4 |  |  | |  |
|  |  |  |  |  |  |  |  |
|  |  |  |  |  |  |  |  |
|  |  |  |  |  |  |  |  |
|  | *topA* | DNA topoisomerase I [EC:5.99.1.2] | 1 |  |  | |  |
|  |  | Toxin of toxin-antitoxin (TA) system, containing PIN domain for nucleic acid binding  Toxin of toxin-antitoxin (TA) system, containing PIN domain for nucleic acid binding | 2 |  |  | |  |
|  |  |  |  |  |  |  |  |
|  |  | Transcriptional regulator | 1 |  |  | |  |
|  |  | Transcriptional regulator, AbrB family  Transcriptional regulator, AbrB family | 2 |  |  | |  |
|  |  |  |  |  |  |  |  |
|  |  | Transporter (SPLIT GENE)  Transporter (SPLIT GENE) |  | 1 | 2 | |  |
|  |  |  |  |  |  |  |  |
|  |  | Transposase and inactivated derivative  Transposase and inactivated derivative (FRAGMENT)  Transposase and inactivated derivative (SPLIT GENE)  Transposase and inactivated derivative (SPLIT GENE) | 1 | 1 | 2 | | 1 |
|  |  |  |  |  |  |  |  |
|  |  |  |  |  |  |  |  |
|  |  |  |  |  |  |  |  |
|  | *traX* | F pilin acetylation protein TraX | 1 |  |  | |  |
|  | *trmD*  *trmE*  *trmU* | tRNA (guanine-n1)-methyltransferase [EC:2.1.1.31]  tRNA modification GTPase TrmE  tRNA (5-methylaminomethyl-2-thiouridylate)-methyltransferase [EC:2.1.1.61] | 3 |  |  | |  |
|  |  |  |  |  |  |  |  |
|  |  |  |  |  |  |  |  |
|  |  | tRNA/rRNA methyltransferase [EC:2.1.1.-]  tRNA/rRNA methyltransferase [EC:2.1.1.-] | 2 |  |  | |  |
|  |  |  |  |  |  |  |  |
|  | *trpS* | Tryptophanyl-tRNA synthetase [EC:6.1.1.2] | 1 |  |  | |  |
|  | *truA*  *truB* | tRNA pseudouridine synthase A [EC:4.2.1.70]  tRNA pseudouridine synthase B | 2 |  |  | |  |
|  |  |  |  |  |  |  |  |
|  | *trxA*  *trxB1*  *trxB2* | Thioredoxin  Thioredoxin reductase [EC:1.6.4.5]  Thioredoxin reductase [EC:1.6.4.5] | 3 |  |  | |  |
|  |  |  |  |  |  |  |  |
|  |  |  |  |  |  |  |  |
|  |  | Tryptophan repressor binding protein (SPLIT GENE)  Tryptophan repressor binding protein (SPLIT GENE) |  | 1 | 2 | |  |
|  |  |  |  |  |  |  |  |
|  | *tsf*  *tuf* | Elongation factor EF-Ts  Elongation factor EF-Tu | 2 |  |  | |  |
|  |  |  |  |  |  |  |  |
|  | *typA* | GTP-binding protein TypA | 1 |  |  | |  |
|  |  | Type I restriction-modification system methyltransferase subunit (SPLIT GENE)  Type I restriction-modification system methyltransferase subunit (SPLIT GENE)  Type I restriction-modification system methyltransferase subunit (SPLIT GENE)  Type I restriction/modification enzyme endonuclease S subunit  Type I site-specific restriction-modification system, R (restriction) subunit (SPLIT GENE)  Type I site-specific restriction-modification system, R (restriction) subunit (SPLIT GENE)  Type I site-specific restriction-modification system, R (restriction) subunit (SPLIT GENE) |  | 1 | 6 | |  |
|  |  |  |  |  |  |  |  |
|  |  |  |  |  |  |  |  |
|  |  |  |  |  |  |  |  |
|  |  |  |  |  |  |  |  |
|  |  |  |  |  |  |  |  |
|  |  |  |  |  |  |  |  |
|  | *tyrS* | Tyrosyl-tRNA synthetase [EC:6.1.1.1] | 1 |  |  | |  |
|  | *ubiA*  *ubiB*  *ubiD*  *ubiE*  *ubiG*  *ubiH*  *ubiX* | 4-hydroxybenzoate octaprenyltransferase [EC:2.5.1.-]  2-polyprenylphenol 6-hydroxylase [EC:1.14.13.-]  UbiD family decarboxylases  Ubiquinone/menaquinone biosynthesis methlytransferase UbiE [EC:2.1.1.-]  Ubiquinone biosynthesis O-methyltransferase [EC:2.1.1.-]  2-polyprenyl-6-methoxyphenol 4-hydroxylase [EC:1.14.13.-] | 7 |  |  | |  |
|  |  |  |  |  |  |  |  |
|  |  |  |  |  |  |  |  |
|  |  |  |  |  |  |  |  |
|  |  |  |  |  |  |  |  |
|  |  |  |  |  |  |  |  |
|  |  | 3-octaprenyl-4-hydroxybenzoate carboxy-lyase [EC:4.1.1.-] | 1 |  |  | |  |
|  | *udg* | UDP-glucose 6-dehydrogenase | 1 |  |  | |  |
|  | *uhpC* | Sugar phosphate permease | 1 |  |  | |  |
|  | *uppS* | Undecaprenyl pyrophosphate synthetase [EC:2.5.1.31] | 1 |  |  | |  |
|  |  | Uracil-DNA glycosylase, family 4 [EC:3.2.2.-] | 1 |  |  | |  |
|  | *uspA* | Universal stress protein UspA and related nucleotide-binding proteins | 1 |  |  | |  |
|  | *uup* | ABC transporter ATP-binding protein Uup | 1 |  |  | |  |
|  | *uvrA*  *uvrB*  *uvrC*  *uvrD* | Excinuclease ABC subunit A  Excinuclease ABC subunit B  Excinuclease ABC subunit C  DNA helicase II [EC:3.6.1.-] | 4 |  |  | |  |
|  |  |  |  |  |  |  |  |
|  |  |  |  |  |  |  |  |
|  |  |  |  |  |  |  |  |
|  | *vacJ* | VacJ lipoprotein precursor | 1 |  |  | |  |
|  | *valS* | Valyl-tRNA synthetase [EC:6.1.1.9] | 1 |  |  | |  |
|  | *vapB1*  *vapC3* | Antitoxin of toxin-antitoxin (TA) system VapB  Toxin of toxin-antitoxin (TA) system VapC, containing PIN domain for nucleic acid binding | 2 |  |  | |  |
|  |  |  |  |  |  |  |  |
|  |  | Variable membrane protein-like protein (SPLIT GENE)  Variable membrane protein-like protein (SPLIT GENE)  Variable membrane protein-like protein (SPLIT GENE) |  | 1 | 3 | |  |
|  |  |  |  |  |  |  |  |
|  |  |  |  |  |  |  |  |
|  | *virB10*  *virB11*  *virB2*  *virB3*  *virB4-1*  *virB4-2*  *virB6-1*  *virB6-2*  *virB6-2*  *virB6-3*  *virB6-4*  *virB6-5*  *virB8-1*  *virB8-2*  *virB9-1*  *virB9-2*  *virD4* | VirB10  VirB11  VirB2-like protein  VirB3  VirB4  VirB4  VirB6  VirB6 (SPLIT GENE)  VirB6 (SPLIT GENE)  VirB6  VirB6  VirB6  VirB8  VirB8  VirB9  VirB9  VirD4 | 15 | 1 | 2 | |  |
|  |  |  |  |  |  |  |  |
|  |  |  |  |  |  |  |  |
|  |  |  |  |  |  |  |  |
|  |  |  |  |  |  |  |  |
|  |  |  |  |  |  |  |  |
|  |  |  |  |  |  |  |  |
|  |  |  |  |  |  |  |  |
|  |  |  |  |  |  |  |  |
|  |  |  |  |  |  |  |  |
|  |  |  |  |  |  |  |  |
|  |  |  |  |  |  |  |  |
|  |  |  |  |  |  |  |  |
|  |  |  |  |  |  |  |  |
|  |  |  |  |  |  |  |  |
|  |  |  |  |  |  |  |  |
|  |  |  |  |  |  |  |  |
|  |  | WD40-like repeat | 1 |  |  | |  |
|  | *xerC*  *xerD* | Tyrosine recombinase XerC  Tyrosine recombinase XerD | 2 |  |  | |  |
|  |  |  |  |  |  |  |  |
|  | *xseA*  *xseB* | Exodeoxyribonuclease VII, large subunit [EC:3.1.11.6]  Exodeoxyribonuclease VII small subunit [EC:3.1.11.6] | 2 |  |  | |  |
|  |  |  |  |  |  |  |  |
|  | *xth1*  *xth2* | Exodeoxyribonuclease III [EC:3.1.11.2]  Exodeoxyribonuclease III [EC:3.1.11.2] | 2 |  |  | |  |
|  |  |  |  |  |  |  |  |
|  | *yajC* | Preprotein translocase YajC subunit | 1 |  |  | |  |
|  | *ychF* | GTP-binding protein YchF | 1 |  |  | |  |
|  | *yidC* | Preprotein translocase subunit YidC | 1 |  |  | |  |
|  | *yqiX*  *yqiY* | Amino acid ABC transporter substrate binding protein  Amino acid ABC transporter permease protein | 2 |  |  | |  |
|  |  |  |  |  |  |  |  |
|  | *znuA*  *znuB*  *znuC* | Zinc/manganese ABC transporter substrate binding protein  Zinc/manganese ABC transporter permease protein  Zinc ABC transporter ATP-binding protein | 3 |  |  | |  |
|  |  |  |  |  |  |  |  |
|  |  |  |  |  |  |  |  |
|  |  | Hypothetical GTP-binding protein | 1 |  |  | |  |
|  | *capD* | nucleoside-diphosphate sugar epimerase CapD, Putative | 1 |  |  | |  |
|  | *cvpA* | colicin V production membrane protein, Putative | 1 |  |  | |  |
|  | *dprA*  *dprA* | DNA processing protein DprA (SPLIT GENE), Putative  DNA processing protein DprA (SPLIT GENE), Putative |  | 1 | 2 | |  |
|  |  |  |  |  |  |  |  |
|  | *dus* | dihydrouridine synthase Dus, Putative | 1 |  |  | |  |
|  | *gidB* | S-adenosylmethionine-dependent methyltransferase GidB [EC:2.1.-.-], Putative | 1 |  |  | |  |
|  | *hemH*  *hemN* | ferrochelatase [EC:4.99.1.1], Putative  oxygen-independent coproporphyrinogen III oxidase [EC:1.-.-.-], Putative | 2 |  |  | |  |
|  |  |  |  |  |  |  |  |
|  | *hipB* | Putative transcriptional regulator | 1 |  |  | |  |
|  | *lipB* | Putative lipoate-protein ligase B [EC:6.-.-.-] | 1 |  |  | |  |
|  | *n2B* | Putative ATPase n2B | 1 |  |  | |  |
|  | *osmY* | Putative periplasmic or secreted lipoprotein | 1 |  |  | |  |
|  | *ppnK* | Putative inorganic polyphosphate/ATP-NAD kinase [EC:2.7.1.23] | 1 |  |  | |  |
|  |  | Putative 6-pyruvoyl tetrahydropterin synthase [EC:4.2.3.12] | 1 |  |  | |  |
|  |  | AAA+ superfamily ATPase, Putative  AAA+ superfamily ATPase (SPLIT GENE), Putative  AAA+ superfamily ATPase (SPLIT GENE), Putative  AAA+ superfamily ATPase (SPLIT GENE), Putative  AAA+ superfamily ATPase (SPLIT GENE), Putative  AAA+ superfamily ATPase (SPLIT GENE), Putative | 1 | 1 | 5 | |  |
|  |  |  |  |  |  |  |  |
|  |  |  |  |  |  |  |  |
|  |  |  |  |  |  |  |  |
|  |  |  |  |  |  |  |  |
|  |  |  |  |  |  |  |  |
|  |  | acetyltransferase , | 1 |  |  | |  |
|  |  | Acyltransferase, Putative | 1 |  |  | |  |
|  |  | aminomethyltransferase related to GcvT, Putative | 1 |  |  | |  |
|  |  | antitoxin of toxin-antitoxin (TA) system, Putative | 1 |  |  | |  |
|  |  | aspartyl protease, Putative | 1 |  |  | |  |
|  |  | DNA alkylation repair enzyme, Putative | 1 |  |  | |  |
|  |  | endonuclease involved in recombination, Putative | 1 |  |  | |  |
|  |  | glutamine amidotransferase, Putative | 1 |  |  | |  |
|  |  | glycoprotein endopeptidase, Putative | 1 |  |  | |  |
|  |  | hydrolase of the alpha/beta superfamily, Putative | 1 |  |  | |  |
|  |  | hydrolase of the metallo-beta-lactamase superfamily, Putative | 1 |  |  | |  |
|  |  | hydrolase/acyltransferase, Putative  hydrolase/acyltransferase, Putative | 2 |  |  | |  |
|  |  | integral membrane protein, Putative | 1 |  |  | |  |
|  |  | Lyase, Putative | 1 |  |  | |  |
|  |  | membrane protein, Putative  membrane protein, Putative  membrane protein, Putative  membrane protein, Putative  membrane protein, Putative | 5 |  |  | |  |
|  |  |  |  |  |  |  |  |
|  |  |  |  |  |  |  |  |
|  |  |  |  |  |  |  |  |
|  |  |  |  |  |  |  |  |
|  |  | membrane-associated metal-dependent hydrolase, Putative | 1 |  |  | |  |
|  |  | membrane-associated zinc metalloprotease, Putative | 1 |  |  | |  |
|  |  | metal-dependent hydrolase, Putative  metal-dependent hydrolase, Putative | 2 |  |  | |  |
|  |  |  |  |  |  |  |  |
|  |  | Methyltransferase, Putative  Methyltransferase, Putative  Methyltransferase, Putative  Methyltransferase, Putative | 4 |  |  | |  |
|  |  |  |  |  |  |  |  |
|  |  |  |  |  |  |  |  |
|  |  |  |  |  |  |  |  |
|  |  | nucleic-acid-binding protein, containing PIN domain, Putative | 1 |  |  | |  |
|  |  | nucleoside-diphosphate-sugar epimerase, Putative | 1 |  |  | |  |
|  |  | outer surface protein, Putative | 1 |  |  | |  |
|  |  | oxidoreductase protein (SPLIT GENE), Putative  oxidoreductase protein (SPLIT GENE), Putative |  | 1 | 2 | |  |
|  |  |  |  |  |  |  |  |
|  |  | P-loop hydrolase, Putative | 1 |  |  | |  |
|  |  | Permease, Putative  Permease, Putative  Permeases, Putative  Permeases, Putative | 4 |  |  | |  |
|  |  |  |  |  |  |  |  |
|  |  |  |  |  |  |  |  |
|  |  |  |  |  |  |  |  |
|  |  | phage terminase protein (SPLIT GENE), Putative  phage terminase protein (SPLIT GENE), Putative  phage terminase protein (SPLIT GENE), Putative  phage terminase protein (SPLIT GENE), Putative |  | 1 | 4 | |  |
|  |  |  |  |  |  |  |  |
|  |  |  |  |  |  |  |  |
|  |  |  |  |  |  |  |  |
|  |  | Sulfurtransferase, Putative | 1 |  |  | |  |
|  |  | toxin of toxin-antitoxin (TA) system, Putative | 1 |  |  | |  |
|  |  | transcriptional regulator, Putative  transcriptional regulator, Putative  transcriptional regulator, Putative | 3 |  |  | |  |
|  |  |  |  |  |  |  |  |
|  |  |  |  |  |  |  |  |
|  |  | virulence protein, Putative | 1 |  |  | |  |
|  |  | Zn-dependent hydrolases of the beta-lactamase fold (SPLIT GENE), Putative  Zn-dependent hydrolases of the beta-lactamase fold (SPLIT GENE), Putative |  | 1 | 2 | |  |
|  |  |  |  |  |  |  |  |
|  | *rbn* | ribonuclease BN [EC:3.1.-.-], Putative | 1 |  |  | |  |
|  | *rfaL* | lipid A core - O-antigen ligase, Putative |  |  |  | |  |
|  | *rssA*  *rssA*  *rssA*  *rssA* | esterase of the alpha/beta hydrolase superfamily protein (SPLIT GENE), Putative  esterase of the alpha/beta hydrolase superfamily protein (SPLIT GENE), Putative  esterase of the alpha/beta hydrolase superfamily protein (SPLIT GENE), Putative  esterase of the alpha/beta hydrolase superfamily protein (SPLIT GENE), Putative |  | 1 | 4 | |  |
|  |  | Sua5/YciO/YrdC/YwlC family translation factor protein, putative | 1 |  |  | |  |
|  | *tatD* | deoxyribonuclease TatD [EC:3.1.21.-], Putative | 1 |  |  | |  |
|  | *traD_F* | conjugative transfer protein TraD (FRAGMENT), Putative |  |  |  | | 1 |
|  | *yhbH* | sigma(54) modulation protein, Putative | 1 |  |  | |  |
|  |  | Unknown proteins | 303 |  |  | |  |
| **Total** |  |  | 1,112 |  |  | |  |
