# Supplementary figures and images for: Analysis of the Rickettsia africae genome reveals that virulence acquisition in Rickettsia species may be explained by genome reduction
Source: BMC Genomics. 2009 Apr 20;10:166. doi: 10.1186/1471-2164-10-166 (PMC2694212; doi:10.1186/1471-2164-10-166)

## Slide 1
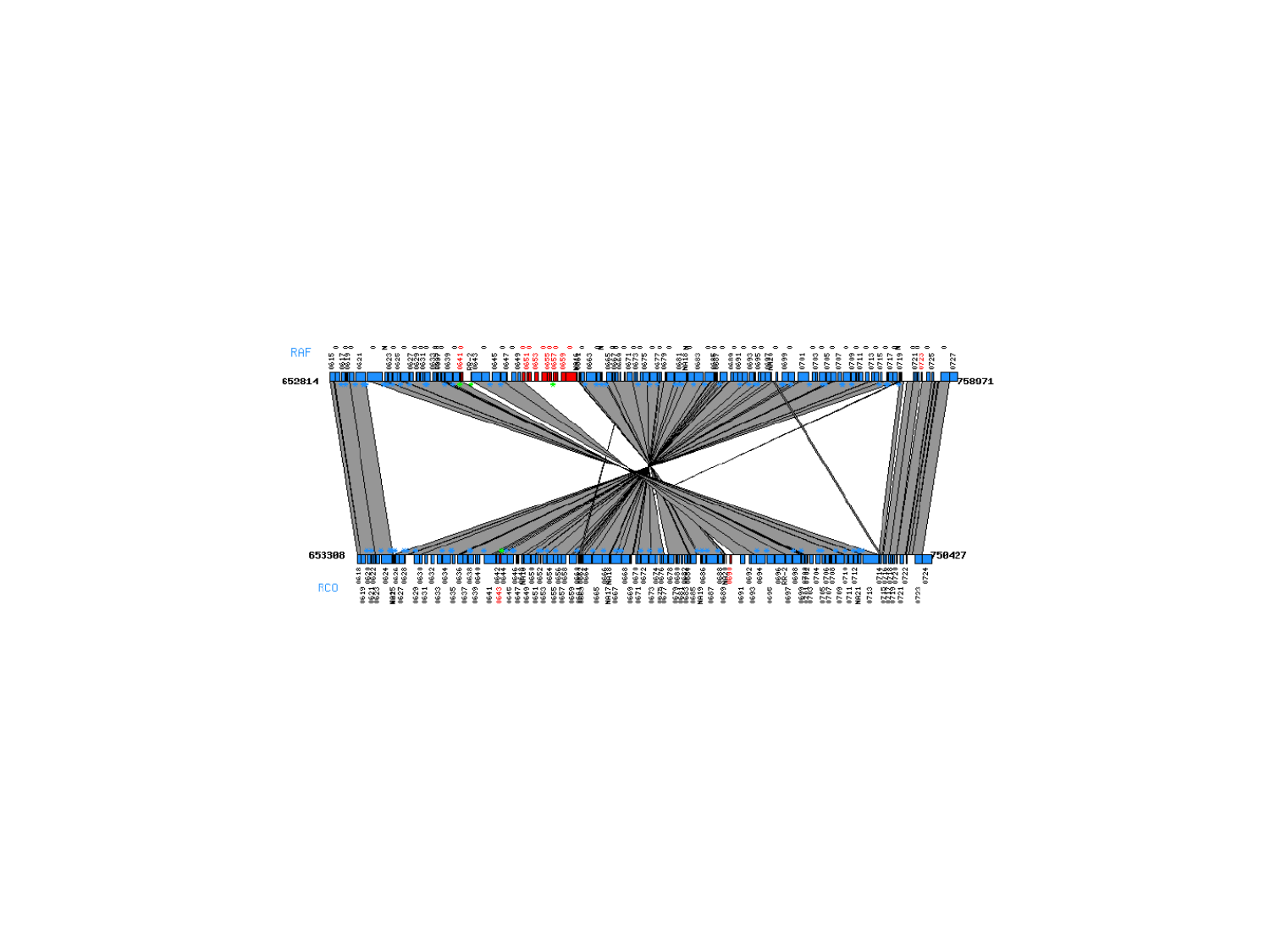

Supplement: Additional file 3 — Inversion observed by alignment of the R. africae (up) and R. conorii (down) genomes. The Figure shows an alignment of the R. conorii and R. africae genomes. [file 1471-2164-10-166-S3.ppt]

## Slide 1
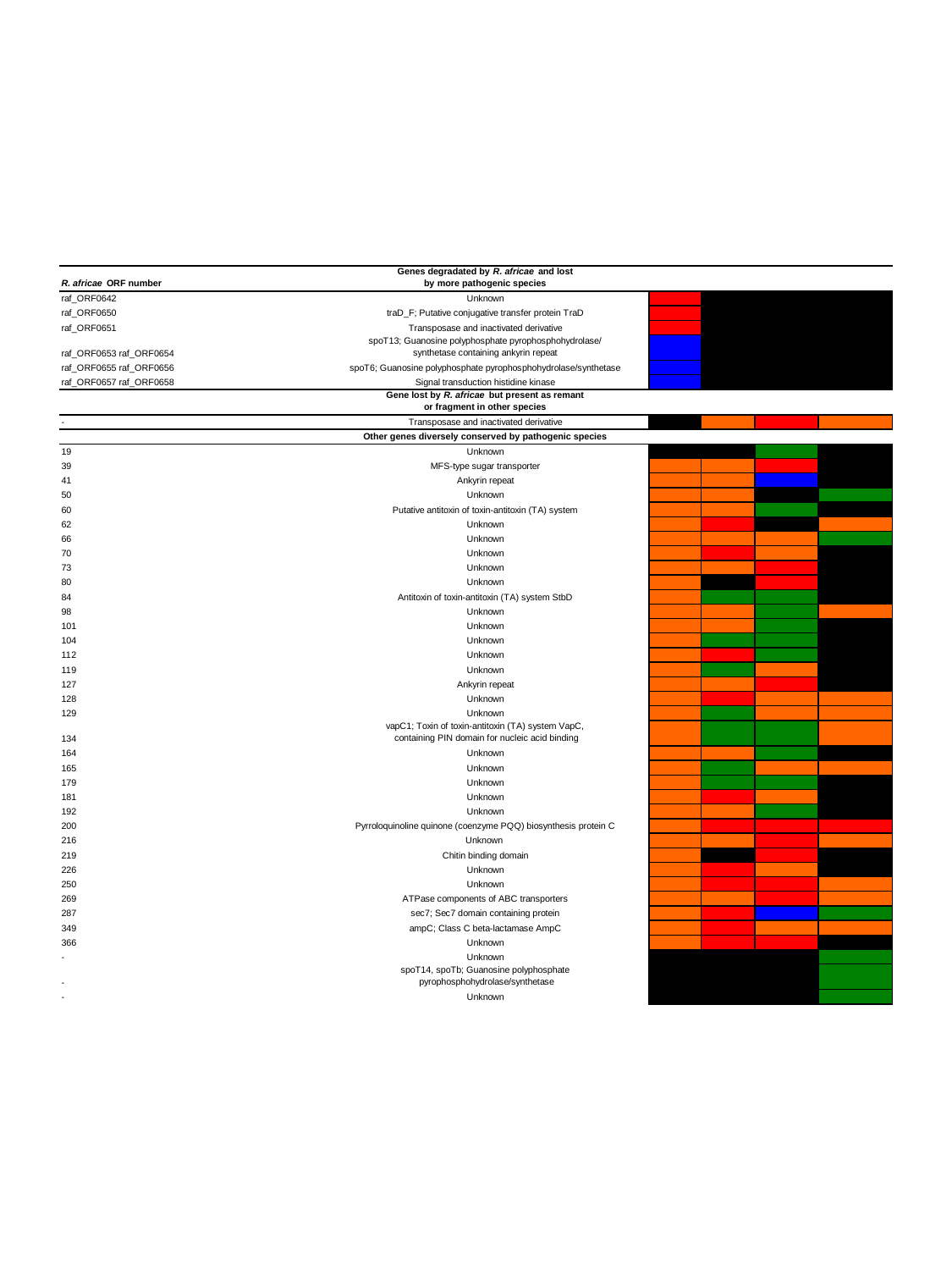

## Slide 2
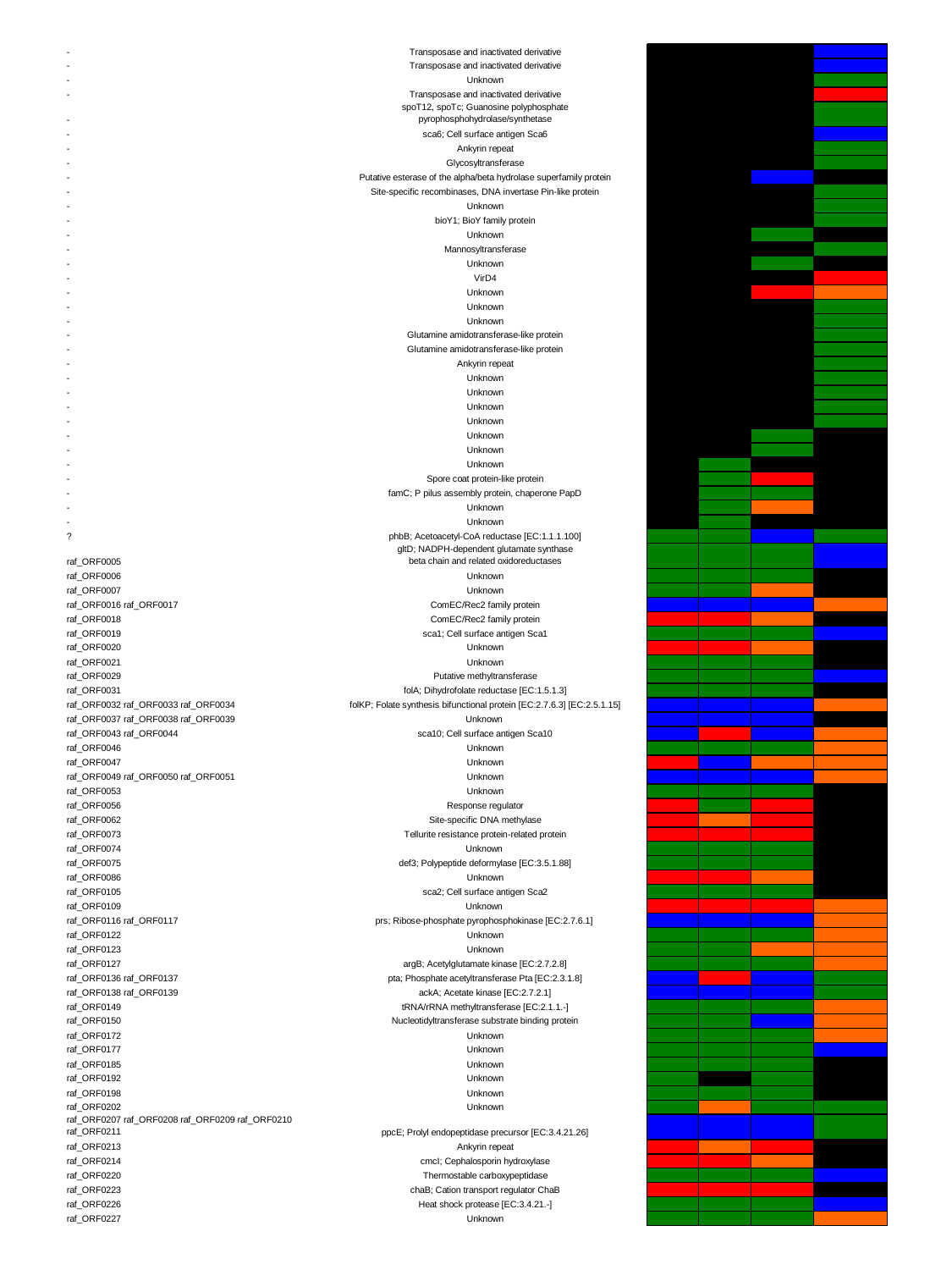

## Slide 3
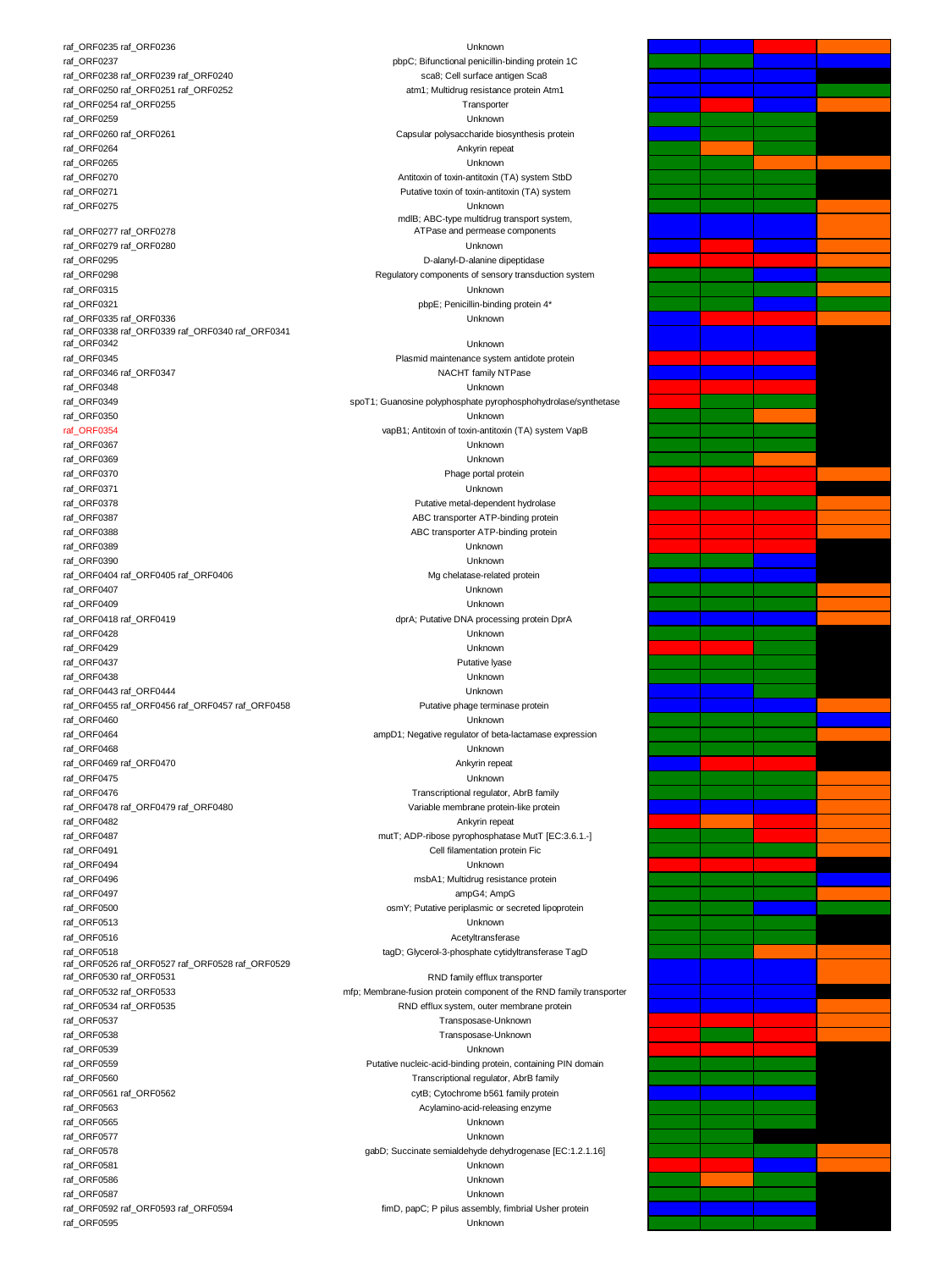

## Slide 4
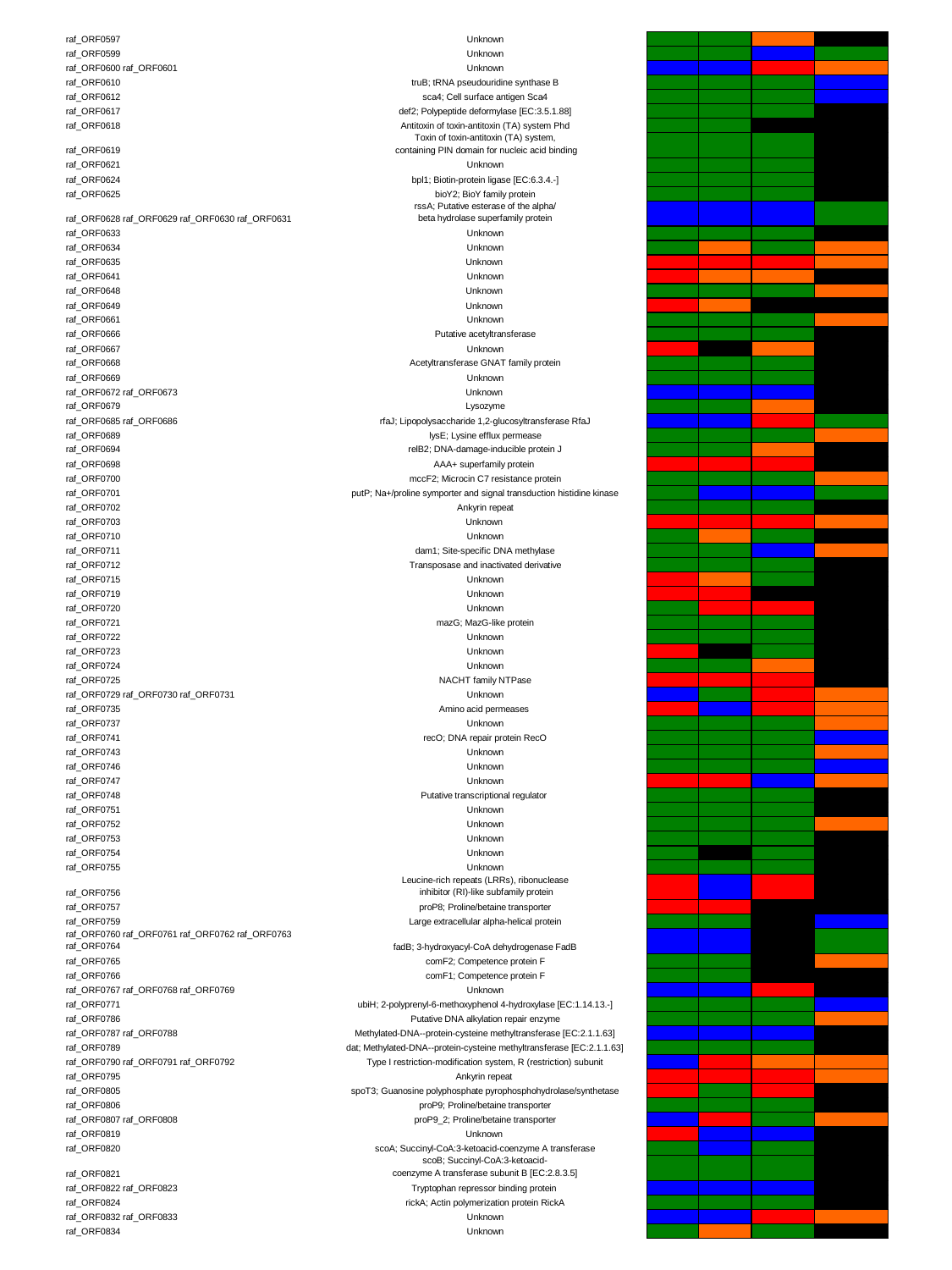

## Slide 5
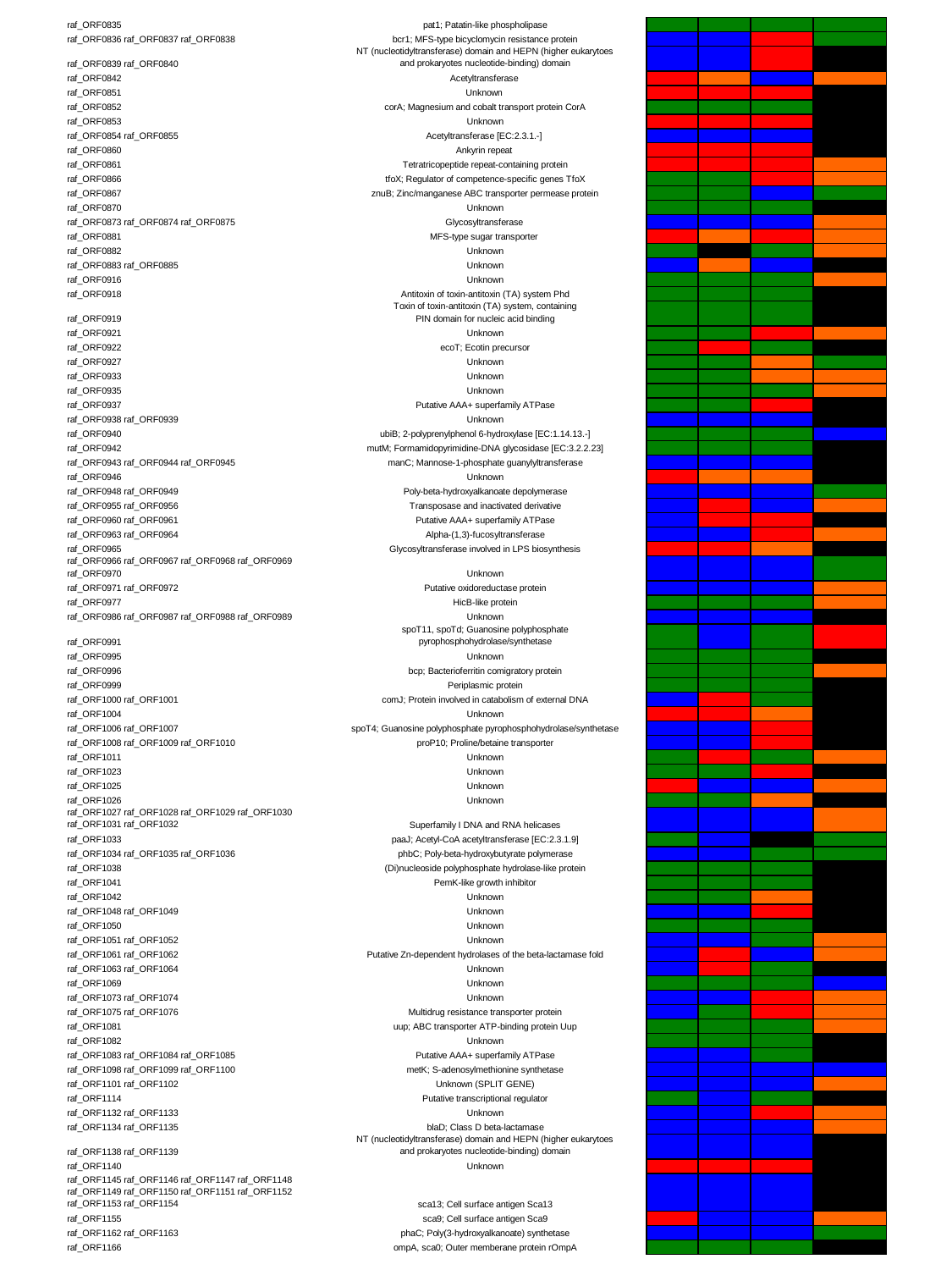

## Slide 6
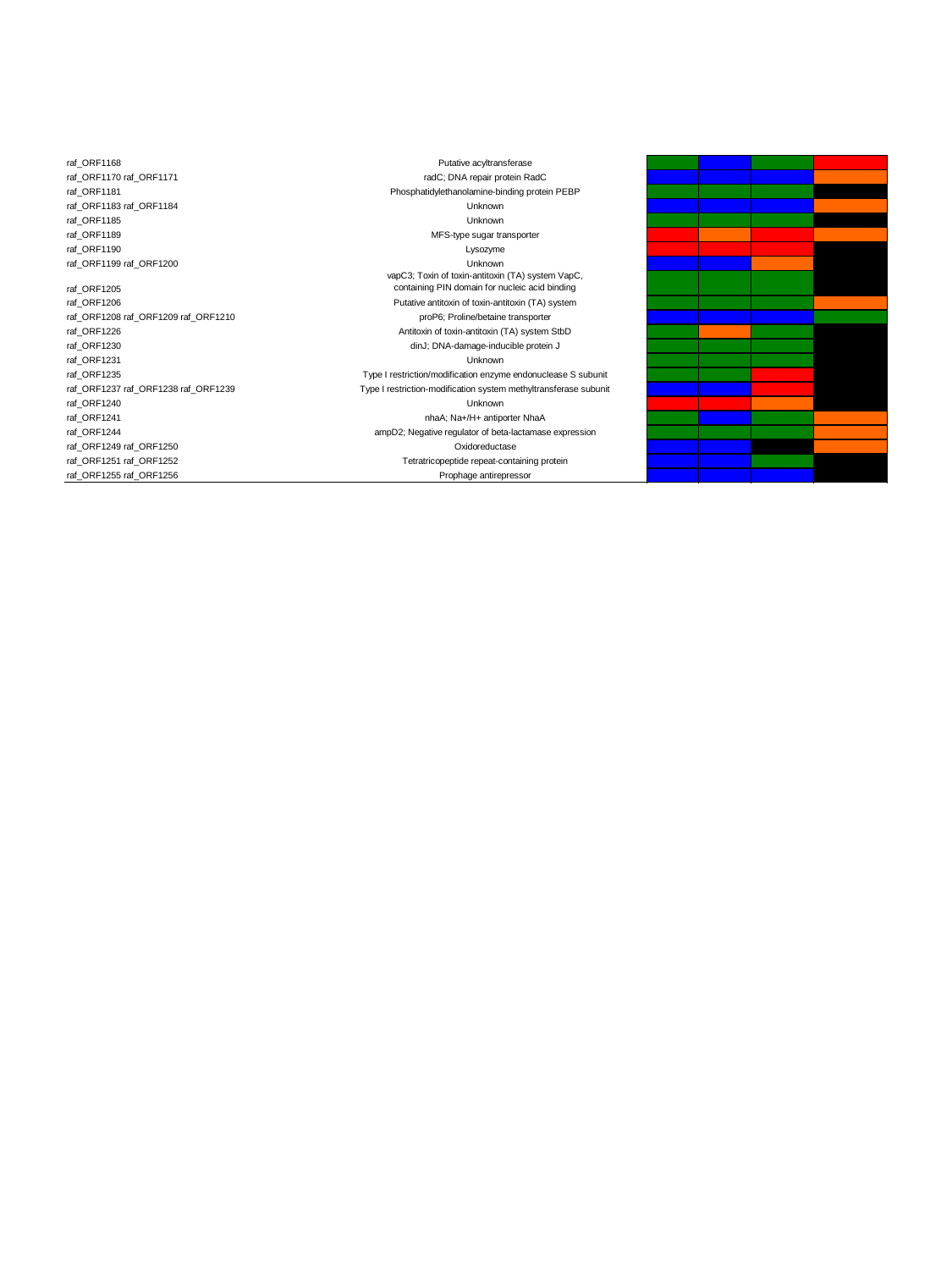

Supplement: Additional file 4 — Schematic representation of the genes diversely conserved in R. africae in comparison with highly pathogenic rickettsiae. The state of a gene is represented by a small box colored in green (full-length), blue (pseudogene), red (fragment), orange (remnant) or black (absent).Gene numbers are indicated in the left column. The Figure shows the gene distribution in R. africae by comparison with highly pathogenic rickettsiae. [file 1471-2164-10-166-S4.ppt]

## Slide 1
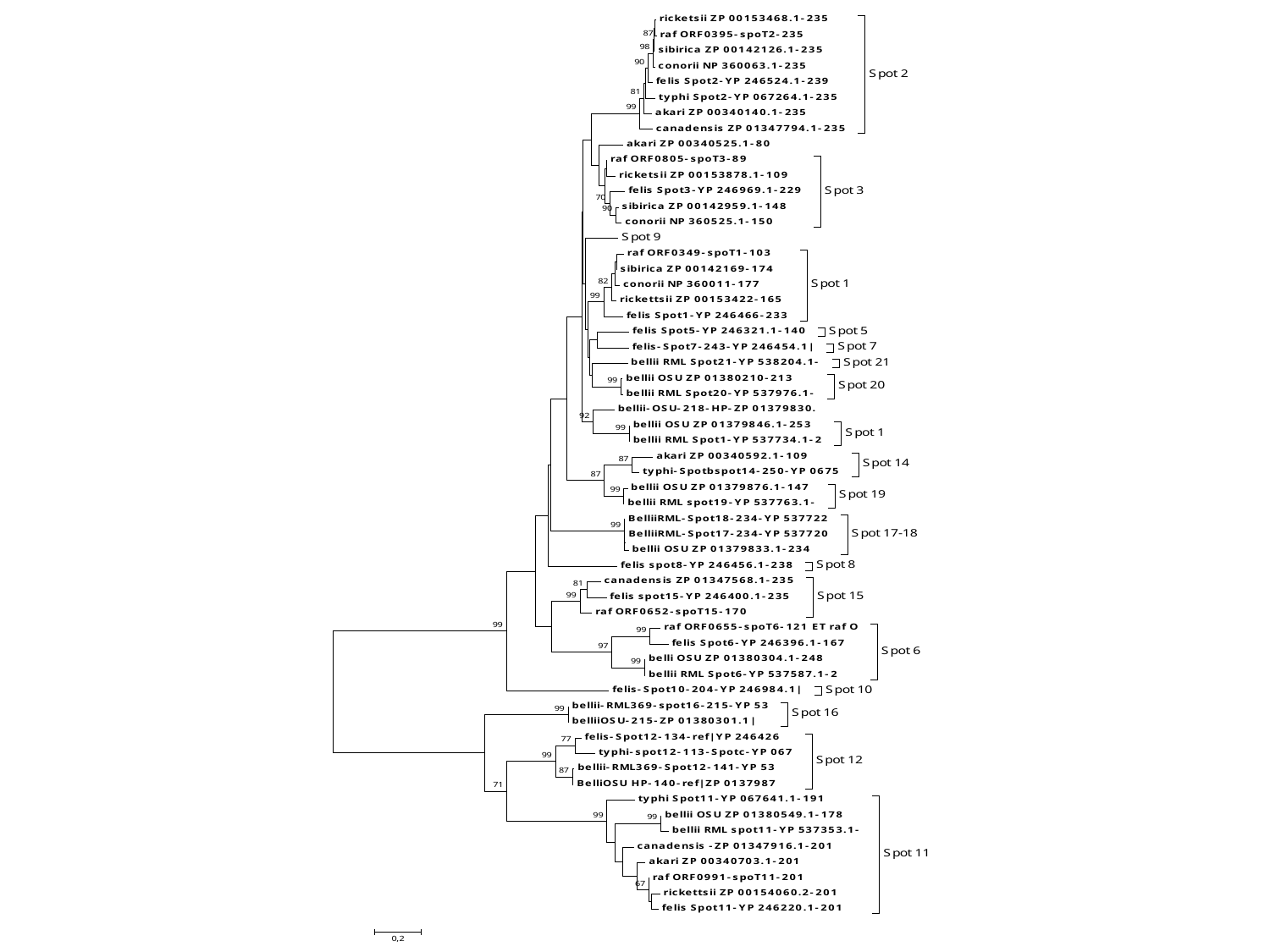

Supplement: Additional file 7 — Phylogenetic tree showing the organization of spoT genes in Rickettsia species. Phylogenetic relationships were inferred from aligned sequences using the Mega3.1 software with the Neighbor-Joining method. Bootstrap values are indicated at the nodes. The Figure is a phylogenetic tree showing the organization of spoT genes in Rickettsia species. [file 1471-2164-10-166-S7.ppt]

## Slide 1
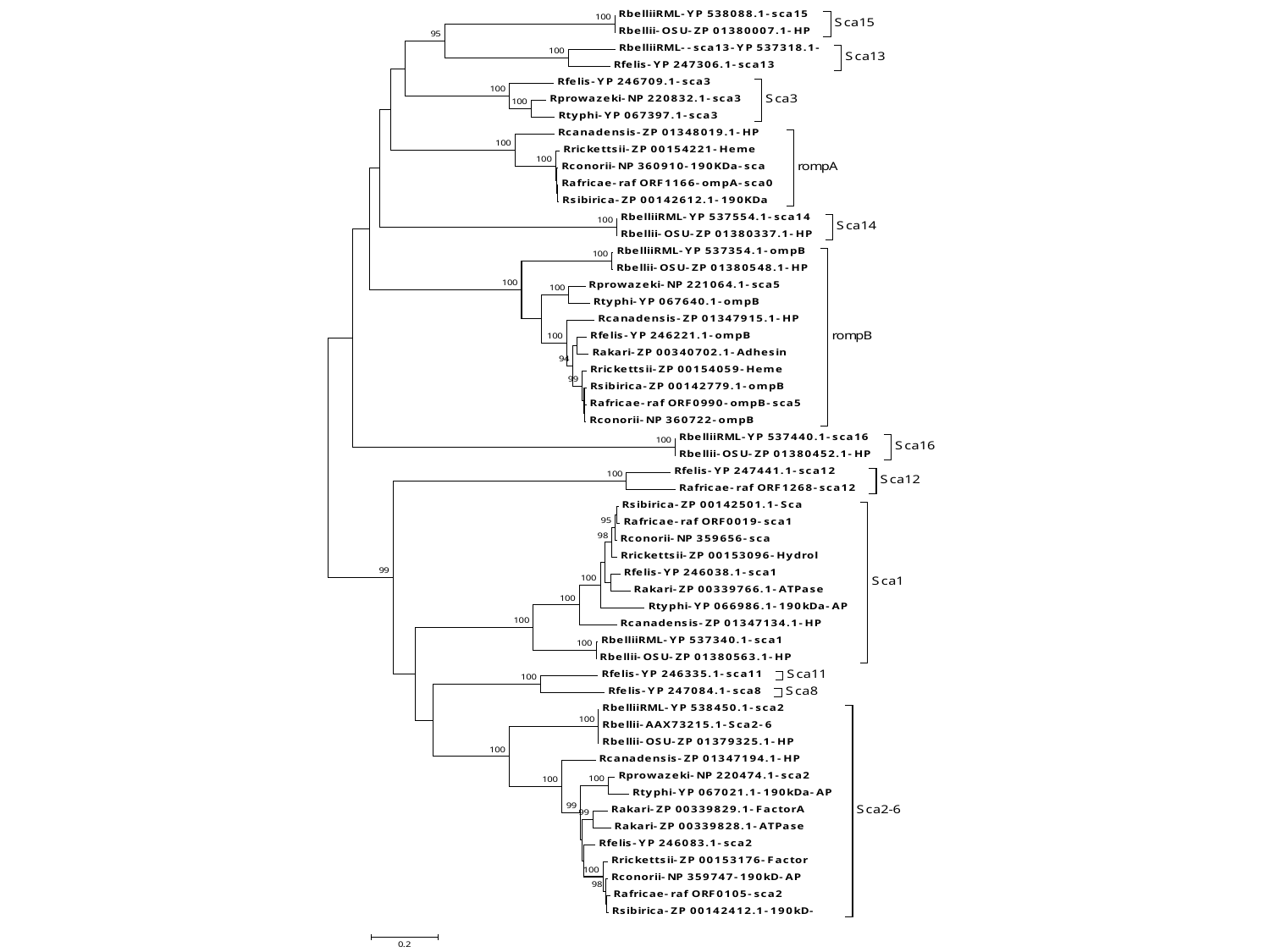

Supplement: Additional file 9 — Phylogenetic tree showing the organization of sca genes in Rickettsia species. Phylogenetic relationships were inferred from aligned sequences using the Mega3.1 software with the Neighbor-Joining method. Bootstrap values are indicated at the nodes. The Figure is a phylogenetic tree showing the organization of sca genes in Rickettsia species. [file 1471-2164-10-166-S9.ppt]

## Slide 1
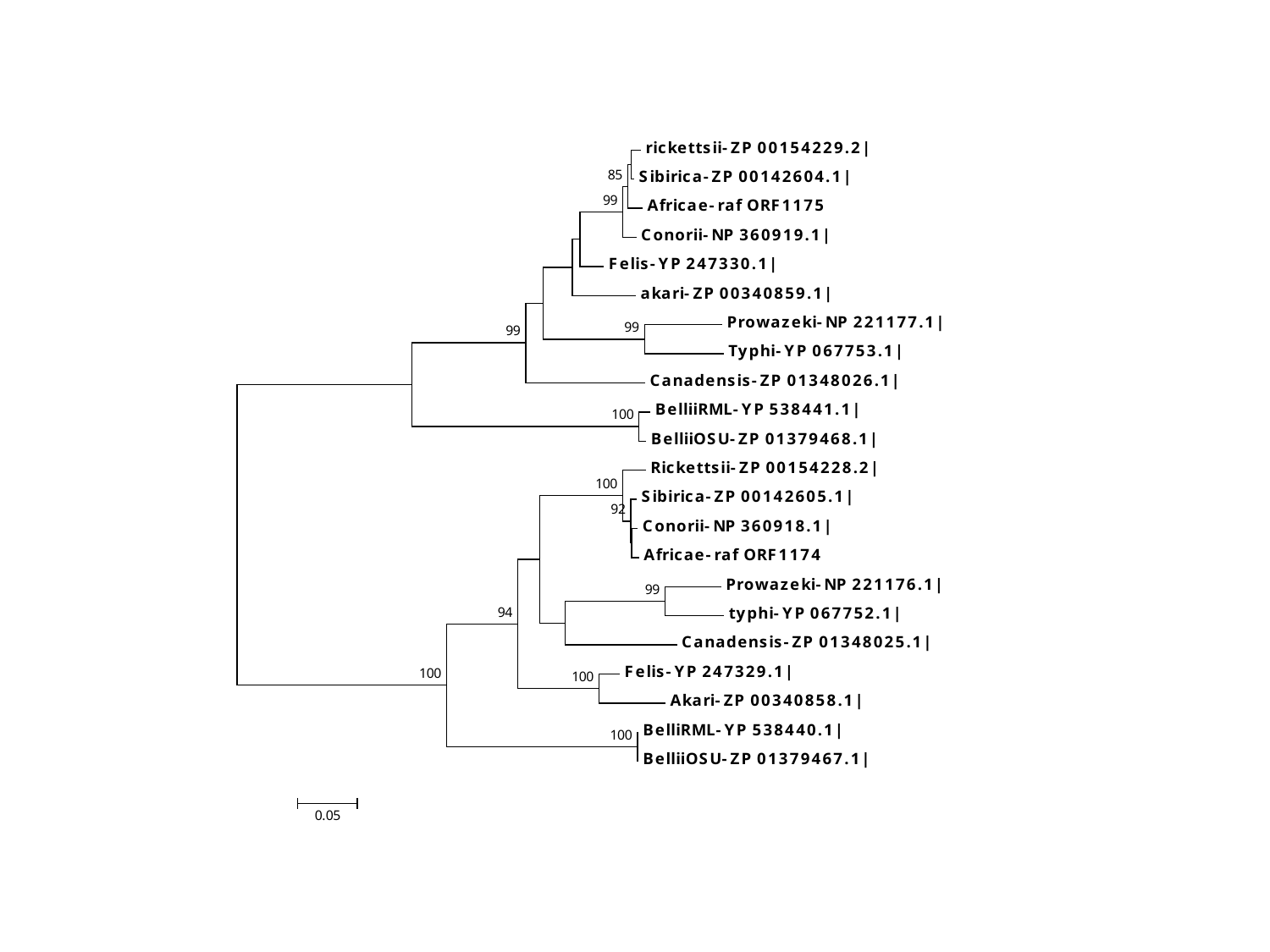

Supplement: Additional file 10 — Phylogenetic tree showing the organization of adr genes in Rickettsia species. Phylogenetic relationships were inferred from aligned sequences using the Mega3.1 software with the Neighbor-Joining method. Bootstrap values are indicated at the nodes. The Figure is a phylogenetic tree showing the organization of adr genes in Rickettsia species. [file 1471-2164-10-166-S10.ppt]

## Slide 1
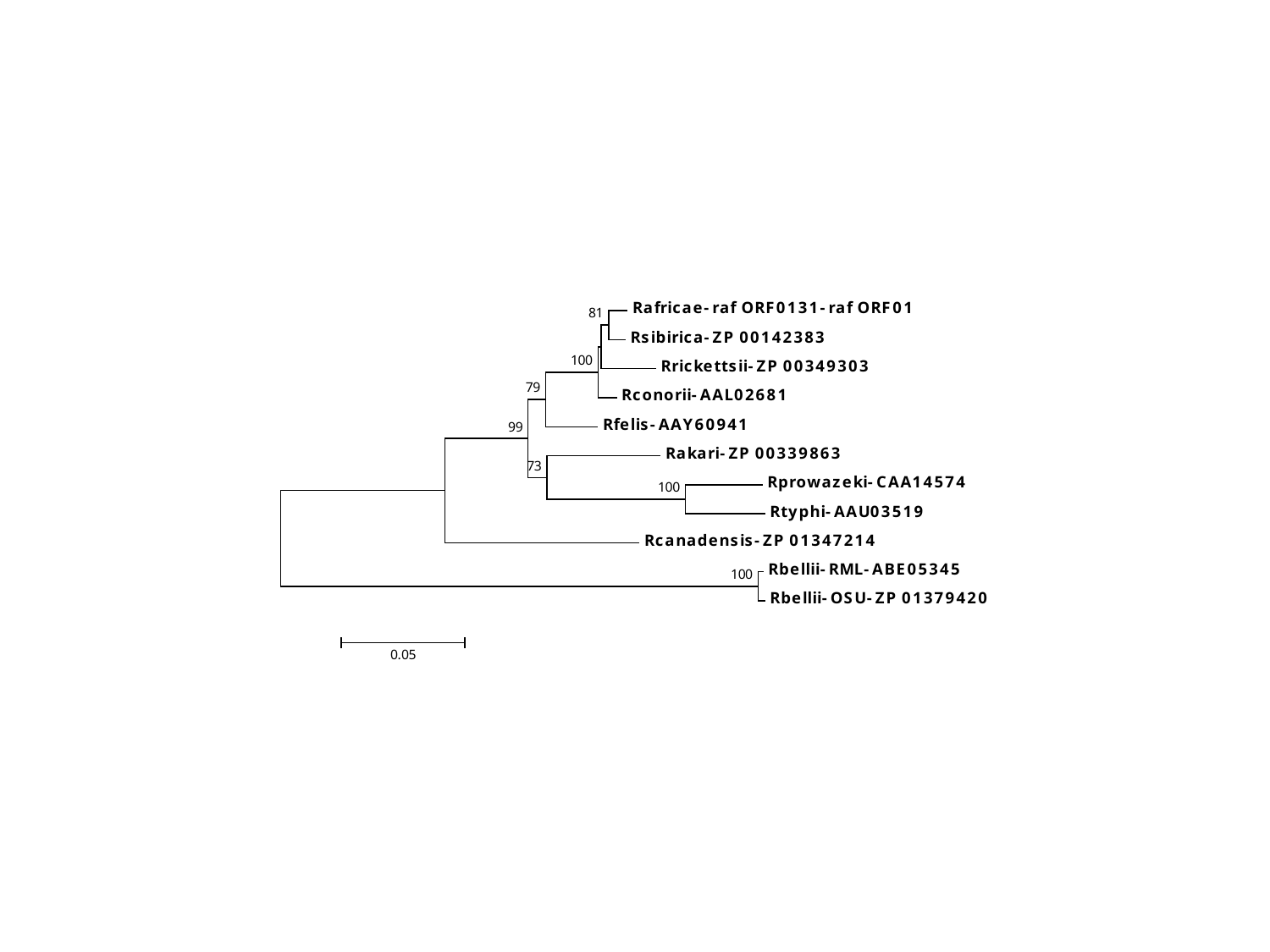

Supplement: Additional file 12 — Phylogenetic tree showing the organization of virB6-2 genes in Rickettsia species. Phylogenetic relationships were inferred from aligned sequences using the Mega3.1 software with the Neighbor-Joining method. Bootstrap values are indicated at the nodes. The Figure is a phylogenetic tree showing the organization of virB6-2 genes in Rickettsia species. [file 1471-2164-10-166-S12.ppt]

## Slide 1
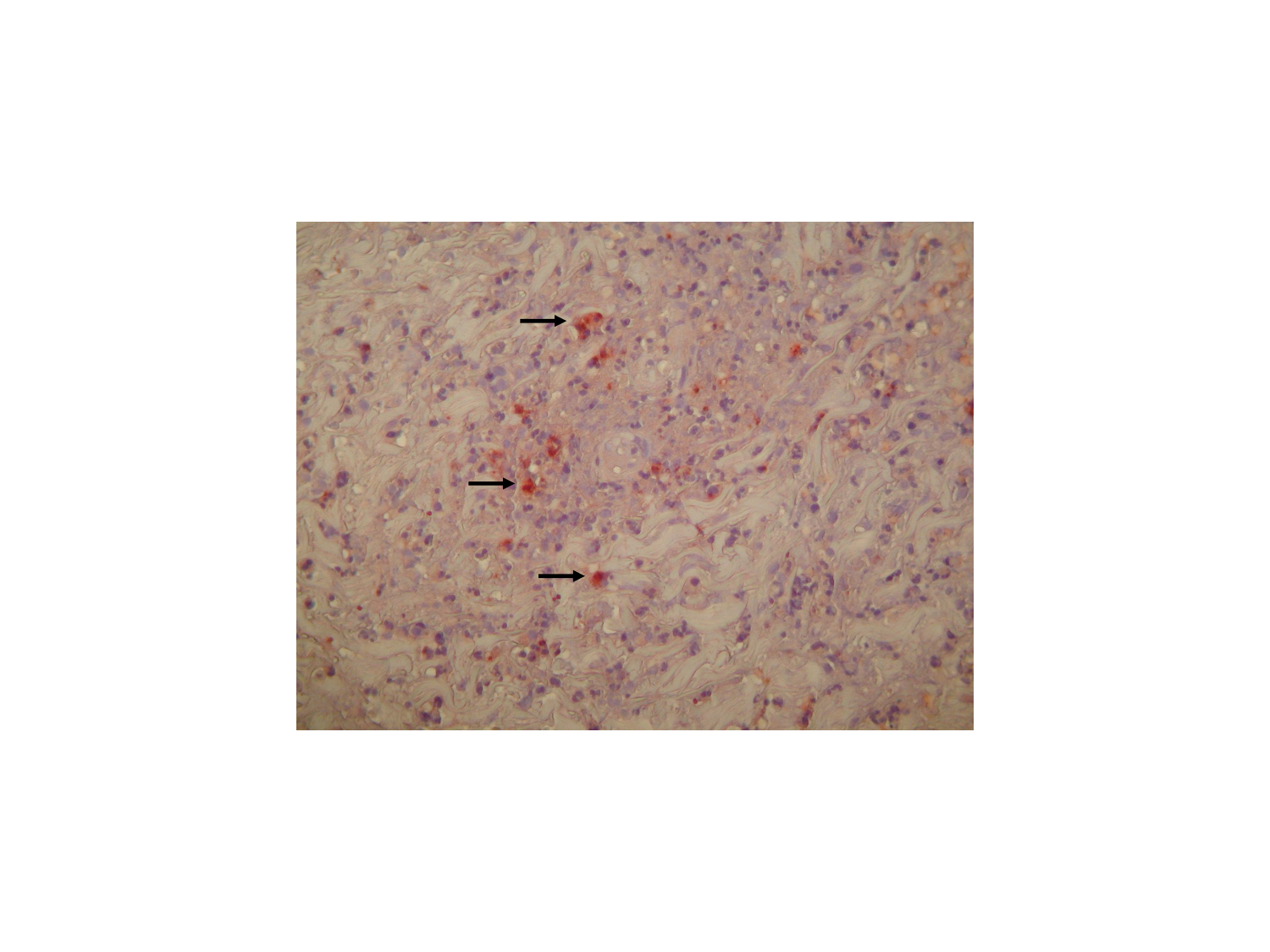

Supplement: Additional file 14 — Immunohistochemical detection of R. africae (arrows) in the inoculation eschar of a patient with ATBF (monoclonal rabbit anti-R. africae antibody used at a dilution of 1:1,000 and hematoxylin counterstain; original magnification ×250). The Figure shows the presence of R. africae in the inoculation eschar of a patient with ATBF, revealed by immunohistochemistry. [file 1471-2164-10-166-S14.ppt]
