# Supplementary material for: Analysis of the Rickettsia africae genome reveals that virulence acquisition in Rickettsia species may be explained by genome reduction
Source: BMC Genomics. 2009 Apr 20;10:166. doi: 10.1186/1471-2164-10-166 (PMC2694212; doi:10.1186/1471-2164-10-166)
Supplement: Additional file 5 — PCR-detection of R. africae and in Amblyomma ticks. Results are indicated as number of ticks positive/number tested. The Table includes the results from PCR detection of the R. africae chromosome and plasmid in ticks. [file 1471-2164-10-166-S5.doc]

| **Country** | ***R. africae* chromosome** | *R. africae* pRA plasmid |
| --- | --- | --- |
| Sudan | 7/9 | 3/3 |
| Madagascar | 4/4 | 3/3 |
| Somalia | 0/2 | * |
| Mali | 8/8 | 3/3 |
| Niger | 7/7 | 1/6 |
| Central African Republic | 21/24 | 1/6 |
| Ivory Coast | 52/59 | 2/3 |
| Djibouti | 0/1 | * |
| Guadeloupe | 36/37 | 3/4 |
| Martinique | 2/2 | 2/2 |
| St Kitts and Nevis | 2/2 | 2/2 |

* not tested
