# Supplementary material for: Analysis of the Rickettsia africae genome reveals that virulence acquisition in Rickettsia species may be explained by genome reduction
Source: BMC Genomics. 2009 Apr 20;10:166. doi: 10.1186/1471-2164-10-166 (PMC2694212; doi:10.1186/1471-2164-10-166)
Supplement: Additional file 6 — Rickettsia africae strains used in this study. The table lists all R. africae strains used in this study. [file 1471-2164-10-166-S6.doc]

| **Strain name** | **Strain source** | **CSUR^a^ accession number** |
| --- | --- | --- |
| ESF-5 ^T^ | Tick | CSUR R15 |
| URRAFMFe12 | Eschar biopsy | CSUR R16 |
| URRAFPFe15 | Eschar biopsy | CSUR R17 |
| URRAFCFMFEe16 | Eschar biopsy | CSUR R18 |
| URRAFCFe20 | Eschar biopsy | CSUR R19 |
| URRAFCFe21 | Eschar biopsy | CSUR R20 |
| URRAFM23 | Eschar biopsy | CSUR R21 |
| URRAFBDFEe24 | Eschar biopsy | CSUR R22 |
| URRAFPFEe26A | Eschar biopsy | CSUR R23 |
| URRAFPFEe26B | Eschar biopsy | CSUR R24 |
| URRAFMFEe34 | Eschar biopsy | CSUR R25 |
| URRAFPFEe38 | Eschar biopsy | CSUR R26 |
| URRAFMFEe42 | Eschar biopsy | CSUR R27 |
| URRAFMFEe43 | Eschar biopsy | CSUR R28 |
| URRAFMeFEe63 | Eschar biopsy | CSUR R29 |
| URRAFM81 | Tick | CSUR R30 |
| URRAFGRFEe84 | Eschar biopsy | CSUR R31 |
| URRAFGRFEe85 | Eschar biopsy | CSUR R32 |
| URRAF87 | Eschar biopsy | CSUR R33 |
| URRAFGRFEe89 | Eschar biopsy | CSUR R34 |
| URRAFORFEe90 | Eschar biopsy | CSUR R35 |
| URRAFOL93 | Eschar biopsy | CSUR R36 |

^a^ CSUR = Collection de Souches de l’Unité des Rickettsies (http://ifr48.timone.univ-mrs.fr/portail2/index.php?option=com_content&task=view&id=96&Itemid=52)
