# Supplementary material for: Analysis of the Rickettsia africae genome reveals that virulence acquisition in Rickettsia species may be explained by genome reduction
Source: BMC Genomics. 2009 Apr 20;10:166. doi: 10.1186/1471-2164-10-166 (PMC2694212; doi:10.1186/1471-2164-10-166)
Supplement: Additional file 8 — R. africae ORFs compared to other available Rickettsia genomes. The table details the distribution of R. africae ORF in other rickettsial genomes. [file 1471-2164-10-166-S8.doc]

| **Category** | **ORF** | **Gene name** | **Functional annotation** | **Raf^*^** | **Rco** | **Rsi** | **Rma** | **Rri** | **Rfe** | **Rak** | **Rpr** | **Rty** | **Rca** | **Rbe** |
| --- | --- | --- | --- | --- | --- | --- | --- | --- | --- | --- | --- | --- | --- | --- |
| **Regulation** | Raf_ORF0349 | *spoT1* | Guanosine polyphosphate pyrophosphohydrolase/synthetase | f | + | + | + | + | + | - | - | - | - | - |
|  | Raf_ORF0395 | *spoT2*  *spoTa* | Guanosine Guanosine polyphosphate pyrophosphohydrolase/synthetase | + | + | + | + | + | + | + | - | + | + | - |
|  | Raf_ORF0805 | *spoT3* | Guanosine polyphosphate pyrophosphohydrolase/synthetase | f | f | f | + | f | + | f | - | - | - | - |
|  | Raf_ORF1006  Raf_ORF1007 | *spoT4-1*  *spoT4-2* | Guanosine Guanosine polyphosphate pyrophosphohydrolase/synthetase | +  f | +  f | +  - | +  - | +  - | f  + | -  - | -  - | -  - | +  - | -  - |
|  | Raf_ORF0655  Raf_ORF0656 | *spoT6* | Guanosine polyphosphate pyrophosphohydrolase/synthetase | s | - | - | + | - | f | - | - | - | - | + |
|  | Raf_ORF0991 | *spoT11*  *spoTd* | Guanosine Guanosine polyphosphate pyrophosphohydrolase/synthetase | + | - | - | + | + | + | + | - | + | + | + |
|  | Raf_ORF0653  Raf_ORF0654 | *spoT13* | Guanosine polyphosphate pyrophosphohydrolase/synthetase containing ankyrin repeat | s | - | - | + | - | + | - | - | - | - | - |
|  | Raf_ORF0652 | *spoT15* | Guanosine polyphosphate pyrophosphohydrolase/synthetase | f | - | - | - | - | + | - | - | - | + | - |
|  | Raf_ORF0103 | *proP1* | Proline/betaine transporter | + | + | + | + | + | + | + | + | + | + | + |
|  | Raf_ORF0396 | *proP2* | Proline/betaine transporter | + | + | + | + | + | + | + | + | + | + | - |
|  | Raf_ORF0477 | *proP3* | Proline/betaine transporter | + | + | + | + | + | + | + | + | + | + | + |
|  | Raf_ORF0953 | *proP4* | Proline/betaine transporter | + | + | + | + | + | + | + | + | + | + | + |
|  | Raf_ORF1065 | *proP5* | Proline/betaine transporter | + | + | + | + | + | + | + | + | + | + | + |
|  | Raf_ORF1208  Raf_ORF1209  Raf_ORF1210 | *proP6* | Proline/betaine transporter (SPLIT GENE) | s | s | s | s | s | + | s | + | + | s | + |
|  | Raf_ORF1253 | *proP7* | Proline/betaine transporter | + | + | + | + | + | + | + | + | + | + | + |
|  | Raf_ORF0757 | *proP8_2* | Proline/betaine transporter (FRAGMENT) | f | f | f | f | - | - | - | - | - | - | + |
|  | Raf_ORF0806 | *proP9* | Proline/betaine transporter | + | + | + | + | + | - | + | - | + | + | + |
|  | Raf_ORF0807  Raf_ORF0808 | *proP9_2* | Proline/betaine transporter (SPLIT GENE) | s | + | + | + | + | s | s | - | + | + | + |
|  | Raf_ORF1008  Raf_ORF1009  Raf_ORF1010 | *proP10* | Proline/betaine transporter (SPLIT GENE) | s | s | f | f | - | + | - | - | - | - | + |
| **Virulence** | Raf_ORF1166 | *sca0*  *=*  *ompA* | Cell surface antigen protein | + | + | + | + | + | s | s | - | - | + | - |
|  | Raf_ORF0019 | *sca1* | Cell surface antigen protein | + | + | + | + | + | + | + | s | + | + | f |
|  | Raf_ORF0105 | *sca2* | Cell surface antigen protein | + | + | + | + | + | + | + | - | - | - | f |
|  | Raf_ORF0585 | *sca3* | Cell surface antigen protein | f | s | s | f | s | + | s | + | + | - | s |
|  | Raf_ORF0612 | *sca4* | Cell surface antigen protein | + | + | + | + | + | + | + | s | + | + | + |
|  | Raf_ORF0990 | *sca5*  *=*  *ompB* | Cell surface antigen protein | + | + | + | + | + | + | + | + | + | + | + |
|  | Raf_ORF0238  Raf_ORF0239  Raf_ORF0240 | *sca8* | Cell surface antigen protein | s | s | s | s | s | + | s | - | - | - | s |
|  | Raf_ORF1155 | *sca9* | Cell surface antigen protein | f | s | s | s | f | + | - | - | - | r | s |
|  | Raf_ORF0043Raf_ORF0044 | *sca10-1*  *sca10-2*  *sca10-3* | Cell surface antigen protein | **-**  f  + | -  f  + | -  f  + | -  f  + | -  f  s | +  s  - | r  s  - | -  -  - | -  -  - | -  -  - | -  -  - |
|  | *R. felis*  NC_007109  (=RF_0319) | *sca11* | Cell surface antigen protein | - | - | - | - | - | + | - | - | - | - | - |
|  | Raf_ORF1268 | *sca12* | Cell surface antigen protein | **+** | - | - | - | - | f | - | - | - | - | - |
|  | Raf_ORF1145,  1146, 1147,  1148, 1149,  1150, 1151,  1152, 1154 | *sca13* | Cell surface antigen protein | s | s | s | s | s | + | - | - | - | - | s |
|  | *R. conorii*  [NP_360918](http://www.ncbi.nlm.nih.gov/entrez/query.fcgi?cmd=Retrieve&db=Protein&list_uids=15893204&dopt=GenPept)  (=RC1281) | *adr* | Rickettsia adhesin | + | + | + | + | + | + | + | + | + | + | + |
|  | *R. prowazeki*  NP_221177  (=RP828) | *adr* | Rickettsia adhesin | + | + | + | + | + | + | + | + | + | + | + |
|  | Raf_ORF0439 | *asmA* | Outer membrane assembly protein | + | + | + | + | + | + | + | + | + | s | + |
|  | Raf_ORF1098  Raf_ORF1099  Raf_ORF1100 | *metk* | S-adenosylmethionine synthetase | s | s | s | s | s | s | + | + | + | - | - |
|  | Raf_ORF0128 | *virB3* | Type IV secretion system | + | + | + | + | + | + | + | + | + | + | + |
|  | Raf_ORF0129 | *virB4-1* | Type IV secretion system | + | + | + | + | + | + | + | + | + | + | + |
|  | Raf_ORF0130 | *virB6-1* | Type IV secretion system | + | + | + | + | + | + | + | + | + | + | + |
|  | Raf_ORF0131  Raf_ORF0132 | *VirB6-2* | Type IV secretion system | s | + | + | + | + | + | + | + | + | + | + |
|  | Raf_ORF0133 | *virB6-3* | Type IV secretion system | + | + | + | + | + | + | + | + | + | + | + |
|  | Raf_ORF0134 | *virB6-4* | Type IV secretion system | + | + | + | + | + | + | + | + | + | + | + |
|  | Raf_ORF0135 | *virB6-5* | Type IV secretion system | + | + | + | + | + | + | + | + | + | + | + |
|  | Raf_ORF0232 | *virB2* | Type IV secretion system | + | + | + | + | + | + | + | + | + | + | + |
|  | Raf_ORF0358 | *virB9-1* | Type IV secretion system | + | + | + | + | + | + | + | + | + | + | + |
|  | Raf_ORF0359 | *virB8-1* | Type IV secretion system | + | + | + | + | + | + | + | + | + | + | + |
|  | Raf_ORF0361 | *virB8-2* | Type IV secretion system | + | + | + | + | + | + | + | + | + | + | + |
|  | Raf_ORF0362 | *virB9-2* | Type IV secretion system | + | + | + | + | + | + | + | + | + | + | + |
|  | Raf_ORF0363 | *virB10* | Type IV secretion system | + | + | + | + | + | + | + | + | + | + | + |
|  | Raf_ORF0364 | *virB11* | Type IV secretion system | + | + | + | + | + | + | + | + | + | + | + |
|  | Raf_ORF0365 | *virD4* | Type IV secretion system | + | + | + | + | + | + | + | + | + | + | + |
|  | Raf_ORF1109 | *virB4-2* | Type IV secretion system | + | + | + | + | + | + | + | + | + | + | + |
|  | Raf_ORF1002 | - | Putative virulence protein | f | f | f | f | f | + | - | - | - | - | - |
|  | Raf_ORF1161 | *PLD* | Phospholipase D superfamily protein | + | + | + | + | + | + | + | + | + | + | + |
|  | Raf_ORF0835 | *pat* | Patatin-like phospholipase | + | + | + | + | + | - | - | + | + | - | s |
|  | Raf_ORF0824 | *rickA* | Actin polymerization protein | + | + | + | + | + | + | + | - | - | + | + |
|  | Raf_ORF0958 | - | LPS biosynthesis protein | f | + | + | + | + | + | f | + | - | - | + |
|  | Raf_ORF0959 | - | LPS biosynthesis protein | + | + | + | + | + | + | r | + | + | - | - |
|  | Raf_ORF0270  Raf_ORF0271 | - | Antitoxin of toxin-antitoxin (TA) system StbD | s | s | s | s | + | + | + | - | - | - | + |
|  | Raf_ORF1226 | - | Antitoxin of toxin-antitoxin (TA) system StbD | + | - | - | + | + | + | - | - | - | - | - |
|  | Raf_ORF0260  Raf_ORF0261 | - | Capsular polysaccharide biosynthesis | s | + | + | + | + | s | s | - | - | f | + |
|  | Raf_ORF0922 | *ecoT* | Ecotin precursor (FRAGMENT) | s | f | f | f | f | + | - | - | - | f | f |
|  | Raf_ORF0701 | *putP* | Na+/Proline symporter | + | + | + | + | f | + | - | + | + | - | + |
|  | Raf_ORF0783 | *panF* | Na+/Proline symporter | + | + | + | + | + | + | + | + | + | + | f |
|  | Raf_ORF1241 | *nhaA* | Na+/H+ antiporter NhaA | + | s | s | + | + | + | s | - | - | - | s |
|  | Raf_ORF0592  Raf_ORF0593  Raf_ORF0594 | *fimD* | P pilus assembly protein, chaperone PapC | s | s | s | s | s | s | - | - | - | + | + |
|  | Raf_ORF0782 | - | Ankyrin repeat | + | s | - | + | - | - | - | - | - | - | - |
|  | Raf_ORF0264 | - | Ankyrin repeat | f | f | f | + | + | - | - | - | - | - | - |
|  | Raf_ORF0424 | - | Plasmid maintenance system antidote protein | f | f | f | + | f | f | f | - | - | - | - |
|  | Raf_ORF0659 | *dam2* | Site-specific DNA adenine methylase [EC:2.1.1.72] | + | - | - | - | - | + | - | - | - | f | + |
|  | Raf_ORF0650 | *traD*F | Putative conjugative transfer protein TraD | f | - | - | + | - | f | - | - | - | - | + |
|  | Raf_ORF0884 | - | Unknown | + | - | - | - | - | - | - | - | - | - | - |
|  | Raf_ORF0720 | - | Unknown | + | + | - | - | f | - | - | - | - | - | - |
|  | Raf_ORF0793 | - | Unknown | + | f | + | s | - | - | - | - | - | - | - |
|  | Raf_ORF0036 | - | Unknown | + | - | - | s | + | - | f | - | - | - | - |
|  | Raf_ORF0192 | - | Unknown | + | - | + | + | + | - | - | - | - | - | - |
|  | Raf_ORF0754 | - | Unknown | + | f | + | + | + | - | - | - | - | - | - |

***** Raf: *R. africae*; Rco: *R. conorii*; Rsi: *R. sibirica*; Rma: *R. massiliae*; Rri: *R. rickettsii*; Rfe: *R. felis*; Rak: *R. akari*; Rpr: *R. prowazekii*; Rty: *R. typhi*; Rca: *R. canadensis*; Rbe: *R. bellii.*
