# Supplementary material for: Analysis of the Rickettsia africae genome reveals that virulence acquisition in Rickettsia species may be explained by genome reduction
Source: BMC Genomics. 2009 Apr 20;10:166. doi: 10.1186/1471-2164-10-166 (PMC2694212; doi:10.1186/1471-2164-10-166)
Supplement: Additional file 11 — Features of RickA repeat proline-rich motif in R. africae and other SFG rickettsiae. The motif " [EDGKQG]- [NS]-N- [IV]- [PSLTR](0,28)" was used to extract these repeats using a PatternMatchingtool. The table details RickA repeat proline-rich motifs in R. africae and other SFG rickettsiae. [file 1471-2164-10-166-S11.doc]

| **Species** | **Start** | **End** | **Proline-rich motif** | **Length** |
| --- | --- | --- | --- | --- |
| *R. africae* | 316 | 327 | ENNITPPSPLS | 11 |
|  | 327 | 357 | ENNIPPSPPPPPPPPPPPPPPPSPPPPPPPPP | 32 |
| *R. rickettsii* | 313 | 328 | ENNIPPPPPPPPPLP | 15 |
|  | 328 | 341 | DSNIPPPPPPPLP | 13 |
|  | 341 | 355 | GNNIPPPPPPPPPP | 14 |
| *R. conorii* | 316 | 327 | ENNITPPSPLP | 11 |
|  | 327 | 344 | ENNIPSPPPPPPPSPLP | 17 |
|  | 344 | 361 | ENNIPSSPPPPPPPPLP | 17 |
|  | 361 | 379 | ENNIPSPPPPPPPPPPPP | 18 |
| *R. sibirica* | 313 | 324 | ENNITPPSPLP | 11 |
|  | 324 | 339 | ENNIPSPPPPPPPLP | 15 |
|  | 339 | 354 | ENNIPSPPPPPPPLP | 15 |
|  | 354 | 369 | ENNIPSPPPPPPPLP | 15 |
|  | 369 | 385 | ENNIPSPPPPPPPPPP | 16 |
| *R. akari* | 313 | 324 | ENNITRPPLLS | 11 |
|  | 324 | 335 | QNNIPSPPPLS | 11 |
|  | 335 | 349 | KNNIPPPPPPPPPP | 14 |
| *R. felis* | 316 | 327 | ENNVTPPPPLT | 11 |
|  | 327 | 341 | KNNIPPPPPPPPLS | 14 |
|  | 341 | 352 | KNNILPPPPPP | 11 |
