# Supplementary material for: Analysis of the Rickettsia africae genome reveals that virulence acquisition in Rickettsia species may be explained by genome reduction
Source: BMC Genomics. 2009 Apr 20;10:166. doi: 10.1186/1471-2164-10-166 (PMC2694212; doi:10.1186/1471-2164-10-166)
Supplement: Additional file 13 — Comparison of epidemiological and clinical characteristics of Rickettsia species. The table includes data about the epidemiological and clinical characteristics of Rickettsia species. [file 1471-2164-10-166-S13.doc]

| **Rickettsia** | **Usual arthropod hosts** | **Prevalence in arthropods in nature (%) *** | **Impairement of vector fitness** | **Transovarial transmission** | **Severity in humans** |
| --- | --- | --- | --- | --- | --- |
| *R. africae* | Ticks | Up to 100% | No | Yes | low |
| *R. rickettsii* | Ticks | Usually < 1% | Yes | Yes ^†^ | high |
| *R. conorii* | Ticks | Usually < 1% | Yes | Yes ^†^ | high |
| *R. slovaca* | Ticks | Up to 40% | NA | Yes | low |
| *R. sibirica* | Ticks | 10-85% | NA | Yes | high |
| *R. massiliae* | Ticks | Up to 12% | No | Yes | Unknown |
| *R. felis* | Fleas | Up to 12% | No | Yes | mild |
| *R. akari* | Mites | Unknown | NA | Yes | Mild to severe |
| *R. bellii* | Ticks ^¶^ | Up to 12% | NA | Yes | Unknown |
| *R. canadensis* | Ticks | Up to 40% | NA | Yes | Unknown |
| *R. prowazekii* | Human body lice | Related to the epidemic situation | Yes | No | high |
| *R. typhi* | Rat fleas | Usually < 5% |  | Yes | mild |

^*^ limits include bias related to sampling methods

^¶^ ticks from various genera from both ixodid and argasid tick families

^†^ with an apparent negative effect on tick fitness

NA: no data available

rescence signals were detected.
