# Supplementary material for: Analysis of the Rickettsia africae genome reveals that virulence acquisition in Rickettsia species may be explained by genome reduction
Source: BMC Genomics. 2009 Apr 20;10:166. doi: 10.1186/1471-2164-10-166 (PMC2694212; doi:10.1186/1471-2164-10-166)
Supplement: Additional file 15 — Supplementary material and methods. The data provided include detailed material and methods that were used for the genome sequencing and sequence analysis of R. africae. [file 1471-2164-10-166-S15.doc]

Bacteria were purified by treatment with 1% trypsin in K36 buffer for 1 h at 37°C, then centrifuged and digested by DNase I for 1 h at 37°C to reduce eukaryotic DNA contamination. The sample was loaded on two successive renograffin gradients (26-38-45), and the bands of purified bacteria were washed in K36 and treated again with DNase I. After inactivation with 50 mM EDTA, the bacteria were resuspended in TE (pH 7.6), aliquotted into 150 μl tubes, and stored at −80 °C. Depending on the initial concentration, one or two tubes were diluted in 1 ml TNE (10 mM Tris [Ph 7.5], 150 mM NaC1, 2 mM EDTA) and incubated for 5 h at 37 °C in the presence of lysozyme (2 mg/ml). Lysis was performed for 2 h at 37 °C by adding 1% SDS and RNAseI (25μg/ml), followed by an overnight treatment with 1mg/ml proteinase K at 37 °C. After three phenol-chloroform extractions and alcohol precipitation, the DNA was resuspended in 30 μl TE, and its concentration was estimated by agarose gel electrophoresis as previously reported [15].

Pulsed-field gel electrophoresis

The concentrated bacterial suspension was immobilized in 1% (vol/vol) Incert agarose gel blocks (BMA, Rockland, MD, USA). The agarose blocks were digested with proteinase K (1mg/ml) in 1% lauroylsarcosine and 0.5 M EDTA (pH 8) for 24 h at 50°C. Fresh proteinase K was then added and the incubation was continued for 24 h. Blocks were then washed twice in TE for 30 min at room temperature. Proteinase K inactivation was performed by incubation in a 4% phenylmethylsulfonyl fluoride (MBI Fermentas, Burlington, Canada) solution for 1 h at 50°C. This inactivation step was carried out twice. Blocks were then washed two to three times in TE and stored in 0.5 M EDTA (pH 8) at 4°C. Before restriction enzyme digestion, agarose blocks were equilibrated twice with TE for 15 min. Digestion was carried out for 4 h, then fresh enzyme was added, and the incubation was continued overnight. The digested agarose blocks and molecular-weight markers (Low Range PFG Marker, Lambda Ladder PFG Marker [New England Biolabs, Beverly, MA, USA]) were equilibrated in 0.5X TBE (50 mM Tris, 50 mM boric acid, 1 mM EDTA).

Each agarose block was laid in a 1% PFGE agarose (Sigma-Aldrich, St. Louis, MI, USA) solution in 0.5X TBE. Pulsed-field gel electrophoresis was carried out on a CHEF-DR II device (Bio-Rad, Hercules, CA, USA) under different electrophoresis conditions. The 1% agarose gel was run at 200 V using ramped pulse times from 1 to 5 s for 10 h to observe the pattern of small DNA fragments (2-48 kb). For bigger fragments, two consecutive migration conditions were used: (i) a ramping time from 3 to 10 s at 200 V for 12 h, for 48- to 242-kb fragments, then (ii) a ramping time from 20 to 40 s at 180 V for 15 h for 145- to 610-kb fragments.

Shotgun sequencing of *R. africae* genome.

Plasmids were cloned into the electrocompetent Escherichia coli strain DH10B (Invitrogen). Each library was validated on 96 clones after DNA extraction and loading on agarose gel to estimate the real insert size, its homogeneity, and the fraction of empty vector (maximum tolerated: 5%). Then, the 96 clones were sequenced to determine the percentage of contamination by eukaryotic DNA from host cells. Plasmid end-sequencing was carried out using the Big Dye terminator chemistry on an automated ABI 3730 sequencer (PE-Applied Biosystems, Foster City, CA, USA). The shotgun libraries of 3, 5, and 10 kb generated 5,416, 5,481, and 5,030 reads, respectively. A total of 16,227 shotgun reads (10 x coverage) were analyzed and assembled using the Phred, Phrap and Consed software suite [Ewing B 1998. Base-calling of automated sequencer traces using phred. I. Genome Res. 8:175-85]. Sequences were retained as valid when a Phred score higher than 20 was associated with at least 75% of the nucleotides. Finishing was performed to improve the low-quality regions and to fill sequencing and cloning gaps. PCR was directly performed on genomic DNA with specific primers and different Taq polymerases depending on the critical region of interest. All finishing sequencing reactions were carried out using the Big Dye chemistry on an automated ABI 3130 sequencer (PE-Applied Biosystems). The final genome sequence assembly was validated by comparing the restriction patterns obtained by pulsed-field gel electrophoresis and those deduced from the consensus sequence. We analyzed single digests of *R. africae* DNA with the following restriction enzymes: ApaI, Afel, FspI, NruI, SbfI, and SgrAI. One circular plasmid molecule of 12 kbp was identified from assembled sequences. Its structure was controlled by specific primer amplification.

Annotation.

We predicted protein-coding genes (ORFs) using SelfID as previously described [15]. Functional assignments for the ORFs was based on the database searches using BLAST [68] against UniProt [69], NCBI/CDD [70], and SMART [71] databases. In most cases, we applied an E-value threshold of 0.001 for the database searches to retrieve homologues. Detailed analyses using multiple sequence alignments and phylogenetic reconstructions were carried out to assign putative functions to the ORFs, when needed. Orthologous gene relationships between *R. africae* and other Rickettsia species were approximated using the best reciprocal BLAST match criterion. The numbers of transposases, ankyrin/tetratricopeptide repeat-containing genes, and integrases were computed using RPS-BLAST with NCBI/CDD entries related to those domains with a 10^−5^ E-value threshold. tRNA genes were identified using tRNAscan-SE [72]. Repeated DNA sequences were identified using RepeatFinder [81]. To identify Rickettsia palindromic elements, we used hidden Markov models [73] based on the previously identified Rickettsia palindromic element sequences. ClustalW [74], T-coffee [75], and MUSCLE [76] were used to construct multiple sequence alignments. Toxin-antitoxin genes were identified using the Rasta-Bacteria software (http://genoweb.univ-rennes1.fr/duals/RASTA-Bacteria).

**Clonal origin of *R. africae***

We examined *R. africae* within 155 *Amblyomma* sp. ticks from various geographical origins (Table4) using the PCR amplification of the *traD* gene using the *R. africae*-specific primer pair traD-F (5’- caatgcttgatctatttggtag-3’) and traD-R (5’- cttccttttctctaagctatgc-3’), and the probe traD-probe (5’-FAM- ttatggtgctaactccatgcgtgatg-TAMRA-3’).

Evaluation of the prevalence of the *R. africae* plasmid

The presence of the plasmid was estimated using the primer pair 1267F (5’-ccagccattaccgtaatcac-3’) and 1267R (5’-tagtgccttatactcaagttc-3’) and the probe 1267-probe (5’-FAM-gcagaaagtgattaaggcgatcagctg-TAMRA-3’) that is able to detect ORF 1267 encoding a protein of unknown function specific for the plasmid. The presence of the plasmid was examined in 22 strains obtained from patients who contracted the disease in South Africa and conserved in the CSUR (Table 1), PCR-positive eschar biopsies from another 48 patients who developed ATBF following a trip to South Africa, and 32 *Amblyomma* sp. ticks found positive for *R. africae* using the above-described PCR assay (Table 5).

**Evaluation of genetic diversity**

To evaluate the genetic diversity of *R. africae*, we used the multi-spacer typing (MST) method as previously described [51]. The three intergenic spacers used, *i. e.*, *dksA-xerC, mppA-purC* and *rpmE-*tRNAfMet, first selected by comparison of the *R. prowazekii* and *R. conorii* genomes [51], contained no ORF in *R. africae*. This method has been described as the most discriminatory genotyping tool at the intraspecies level in *Rickettsia* sp. [51]. We applied this method to the aforementioned 22 human *R. africae* strains, 48 eschar biopsies, and 32 *Amblyomma* sp. ticks from Sudan (3), Madagascar (3), Mali (3), Niger (6), Central African Republic (6), Ivory Coast (3), Guadeloupe (4), Martinique (2), and S^t^ Kitts and Nevis (2). The obtained sequences were compared to those available in GenBank, and the MST genotypes determined as previously described [51].

**Study of transcription of genes conserved by *R. africae* and degradated or lost by other SFG rickettsiae**

Cell and bacterial culture

*R. africae* were grown both in Vero and XTC cells for 48 h using T25 cm² flasks. Two different temperatures were used for each cells line that is 32 and 37°C in Vero cells and 28 and 32°C for XTC cells. Cells were harvested using glass beads to remove the monolayer cells. Then infected cells were centrifuged at 5,000xg for 10 min at 4°C. For each culture specimen, the supernatant was removed and the pellet resuspended and incubated in TE buffer containing 10mg/ml lysozyme for 10 min at room temperature. RNA samples were extracted and purified using RNeasy-Mini kit (Qiagen) with the RNase free DNase set (Qiagen) step performed for 30 min and stored at -80°C. The yield and quality of RNA were analyzed using the RNA 6000 Nano Labchip kit and 2100 Bioanalyzer (Agilent).

For the amplification of the raf_ORF0036, raf_ORF0064, raf_ORF 0391, raf_ ORF0412, raf_ORF0414, raf_ORF0415, raf_ORF0424, raf_ORF0445, raf_ORF0652, raf_ORF0659, raf_ORF0660, raf_ORF0758, raf_ORF0782, raf_ORF0793, raf_ORF0876, raf_ORF0884, raf_ORF0973, and raf_ORF1002, we used the primer pairs and probes raf_ORF0036F (5’-ttcttcaccgtgtagtcaac-3’), raf_ORF0036R (5’-cgacaagccggtcacat-3’), and raf_ORF0036probe (5’-FAM-ctaacatattgctagaatcttcgattcatac-TAMRA-3’); raf_ORF0064F (5’-cagacaaacgagaacaagtg-3’), raf_ORF0064R (5’-cagacaaacgagaacaagtg-3’), and raf_ORF0064probe (5’-FAM-aagttttggggtttatggcggttccg-TAMRA-3’); raf_ORF0391F (5’-ggttaactaactttcagttag-3’), raf_ORF0391R (5’-tagctatctacagaacctag-3’), and raf_ORF0391probe (5’-FAM-cttggcgagaaaatatttcaatttatcaaag-TAMRA-3’); raf_ORF0412F (5’-attgatgttgacgatacgc-3’), raf_ORF0412R (5’-agaaggaatattgccgaatg-3’), and raf_ORF0412probe (5’-FAM-cagtaattggcgtttgcagcgaaaag-TAMRA-3’); raf_ORF0414F (5’-gtggagtgagggatttaccg-3’), raf_ORF0414R (5’-gccaaaacaaatccacgata-3’), and raf_ORF0414probe (5’-FAM-tggcaagattatgcagtacaaagattgcca-TAMRA-3’); raf_ORF0415F (5’-ctaaagctgcgacaatttcac-3’), raf_ORF0415R (5’-ctcctaatgtaaaagcaccg-3’), and raf_ORF0415probe (5’-FAM-cggtgcaaaaactgtatgttccgttc-TAMRA-3’); raf_ORF0424F (5’-atggaacctatggggattag-3’), raf_ORF0424R (5’-tattggtcttgcatattccc-3’), and raf_ORF0424probe (5’-FAM-gctgatacaatccttcgtttgggtaag-TAMRA-3’); raf_ORF0445F (5’-tagtcggtagtatgtatgcg-3’), raf_ORF0445R (5’-tcttggaggaaactaagagc-3’), and raf_ORF0445probe (5’-FAM-agtgttgatgaggcttgcagatagtg-TAMRA-3’); raf_ORF0652F (5’-gtgtattggaggtaaagaaag-3’), raf_ORF0652R (5’-ctagttactagtatatctgttc-3’), and raf_ORF0652probe (5’-FAM-ggtagtcagatgcgtcaatcaggag-TAMRA-3’); raf_ORF0659F (5’-caatgggtgggtggtaaaag-3’), raf_ORF0659R (5’-aaccgcatcaggatttgtct-3’), and raf_ORF0659probe (5’-FAM-tgaaccatttcttggtggtggagcttt-TAMRA-3’); raf_ORF0660F (5’-gccgaacaatacccagaaca-3’), raf_ORF0660R (5’-cccaataaagcagcaagagc-3’), and raf_ORF0660probe (5’-FAM-caagcccagttaaaacgtaaaatcgtcgg-TAMRA-3’); raf_ORF0758F (5’-tatccttatatgtttggagcg-3’), raf_ORF0758R (5’-tactgaagcaacaatgatagc-3’), and raf_ORF0758probe (5’-FAM-tggctattaaccgtaccgatggtagc-TAMRA-3’); raf_ORF0782F (5’-tcggccattcaagaagtacc-3’), raf_ORF0782R (5’-tgttttagttgagcggtttgaa-3’), and raf_ORF0782probe (5’-FAM-tcgaacagtttcacatcacttacggagca-TAMRA-3’); raf_ORF0793F (5’-aatggcttaggtagtatagac-3’), raf_ORF0793R (5’-cgtatttatatcttggaaactc-3’), and raf_ORF0793probe (5’-FAM-cagaccttttaaattgattgtagacgagg-TAMRA-3’); raf_ORF0876F (5’-aacataagcacggatgcttc-3’), raf_ORF0876R (5’-aacttgtaagcattatgccg-3’), and raf_ORF0876probe (5’-FAM-ggcgagatatctttactaatgggaatg-TAMRA-3’); raf_ORF0884F (5’-ctttcgtttgttgctgctca-3’), raf_ORF0884R (5’-cctgccacagctccatttat-3’), and raf_ORF0884probe (5’-FAM-gatgttgccatggcagtagctgatacaaa-TAMRA-3’); raf_ORF0973F (5’-ttgaatgcaacggcatatgc-3’), raf_ORF0973R (5’-caccagaagccaacaactac-3’), and raf_ORF0973probe (5’-FAM-cggcataatattagtaaatatgcttggc-TAMRA-3’); raf_ORF1002F (5’-actgccgctgatggtaaaac-3’), raf_ORF1002R (5’-tgcaatctttcgtaaaagcag-3’), and raf_ORF1002probe (5’-FAM-gagggatcgctttcagaaaatgggc-TAMRA-3’), respectively. As a positive control, we amplified a fragment of the *rpoB* gene using the primer pair rpoBF (5- cgaacctgctaatatcgaag -3’) – rpoBR (5- gttaaaaccgtaacttcttcag -3’) and the probe afrpobprobe (5’-FAM-cggaaagatatgatctgtcagaagtc-TAMRA-3’).

The fold change (FC) in expression of the target gene relative to *rpoB* was determined as follows: FC = 2^-ΔΔCt^ where ΔΔCt = (CtTarget -CtActin)sample - (CtTarget - CtActin)reference. Ct values were defined as the cycle numbers at which the fluorescence signals were detected.
